# Antibacterial Polyketides from the Deep-Sea Cold-Seep-Derived Fungus *Talaromyces* sp. CS-258

**DOI:** 10.3390/md22050204

**Published:** 2024-04-28

**Authors:** Zhenger Wu, Xiao-Ming Li, Sui-Qun Yang, Bin-Gui Wang, Xin Li

**Affiliations:** 1 CAS and Shandong Province Key Laboratory of Experimental Marine Biology, Institute of Oceanology, Chinese Academy of Sciences, Nanhai Road 7, Qingdao 266071, China; wuzhenger@qdio.ac.cn (Z.W.); lixmqd@qdio.ac.cn (X.-M.L.); suiqunyang@163.com (S.-Q.Y.); 2 University of Chinese Academy of Sciences, Yuquan Road 19A, Beijing 100049, China; 3 Laboratory for Marine Biology and Biotechnology, Qingdao Marine Science and Technology Center, Wenhai Road 1, Qingdao 266237, China

**Keywords:** antibacterial activity, fungal polyketides, cold-seep-derived fungus, *Talaromyces* sp.

## Abstract

Thirty-two fungal polyketide derivatives, including eleven new compounds, namely (3*R*,5′*R*)-5-hydroxytalaroflavone (**1**), talaroisochromenols A–C (**3**, **5**, and **11**), (8*R*,9*R*,10a*R*)-5-hydroxyaltenuene (**13**), (8*R*,9*R*,10a*S*)-5-hydroxyaltenuene (**14**), (8*R*,9*S*,10a*R*)-5-hydroxyaltenuene (**15**), nemanecins D and E (**25** and **26**), 2,5-dimethyl-8-iodochromone (**27**), and talarofurolactone A (**29**), together with one new naturally occurring but previously synthesized metabolite, 6-hydroxy-4-methoxycoumarin (**28**), were isolated and identified from the deep-sea cold-seep-derived fungus *Talaromyces* sp. CS-258. Among them, racemic ((±)-**11**) or epimeric (**13**–**15**, **25**, and **26**) mixtures were successfully separated by chiral or gradient elution HPLC. Meanwhile, compound **27** represents a rarely reported naturally occurring iodinated compound. Their planar structures as well as absolute configurations were determined by extensive analysis via NMR, MS, single-crystal X-ray diffraction, Mosher’s method, and ECD or NMR calculation (with DP4^+^ probability analysis). Possible biosynthetic routes of some isolated compounds, which are related to chromone or isochromone biosynthetic pathways, were put forward. The biological analysis results revealed that compounds **7**, **9, 10, 18**–**22**, **24**, **30**, and **31** showed broad-spectrum antibacterial activities against several human and aquatic pathogens with MIC ranges of 0.5–64 μg/mL.

## 1. Introduction

Polyketides are a big family of secondary metabolites generally produced from a common origin of acetyl-CoA polymerization by connecting acetic acid units *via* condensation reactions and intermediated by a non-reducing group of iterative polyketide synthases (PKSs) [1,2]. Fungal polyketides have attracted considerable attention due to their unique molecular scaffolds and diverse pharmacological activities [3,4,5]. Among them, a series of fungal polyketides, such as griseofulvin, brefeldin, and hypomycetin, have been selected as promising candidates for new antibiotic lead compounds and possess great commercial values [1,3,5].

Deep-sea cold-seep-derived fungi have formed a unique physiological process and metabolic mechanism during their adaptation to the typical chemosynthetic-driven ecosystems with hydrocarbon-rich fluid, strikingly emerging as a promising source for their prolific production of structurally diverse secondary metabolites with various biological properties in very recent years [6,7,8]. Increasing efforts have been made on deep-sea cold-seep fungi to seek and develop biologically active pharmaceutical molecules with antibiotic [7,9,10], antioxidant [11], phytotoxic [12], anti-inflammatory [13], cytotoxic [14], proangiogenic [15,16], and enzyme inhibitory [17] properties.

**Figure 1:**
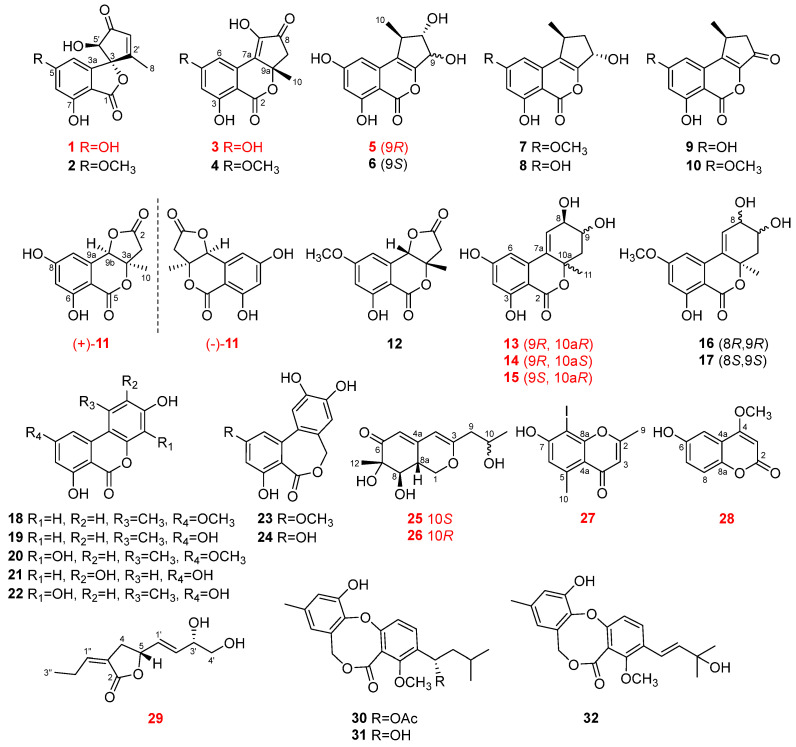
Chemical structures of compounds **1**–**32**.

In the course of forwarding our program on discovering bioactive metabolites from deep-sea cold seep-derived fungi [6,15,16], a fungal strain *Talaromyces* sp. CS-258, which was isolated from a mussel sample collected in a cold seep area in the northeast of the South China Sea at a depth of −1200 m, displayed diverse classes of polyketide derivatives and exhibited antimicrobial activity in a preliminary screening. Intensive chemical investigations on the fermentation broth of the fungus resulted in the isolation and identification of 32 polyketides (Figure 1), including 11 new chromone- or isochromone-derived analogs (compounds **1**, **3**, **5**, **11**, **13**–**15**, **25**–**27**, and **29**) and 1 new naturally occurring coumarin (**28**) that was previously obtained by chemical synthesis [18]. The isolated compounds were assayed for their antimicrobial activities against pathogenic microbes, which demonstrated that eleven polyketides, including **7, 9**, **10, 18**–**22**, **24**, **30**, and **31**, possessed broad-spectrum antibiotic activities against human and aquatic pathogenic bacteria with MIC values ranging from 0.5 to 64 μg/mL. Herein, the details of isolation, structural elucidation, plausible biosynthetic pathways, and bioactivity assays of these compounds are described. The structure–activity relationships (SARs) for these antibacterial polyketides have been briefly discussed in this paper.

## 2. Results and Discussion

### 2.1. Structure Elucidation

**Figure 2:**
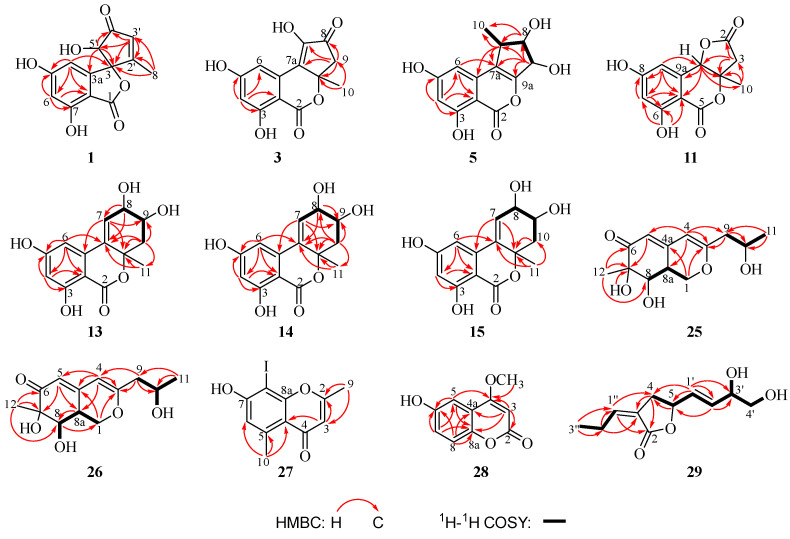
The ^1^H–^1^H COSY and key HMBC correlations of compounds **1**, **3**, **5**, **11**, **13**–**15**, and **25**–**29**.

**Figure 3:**
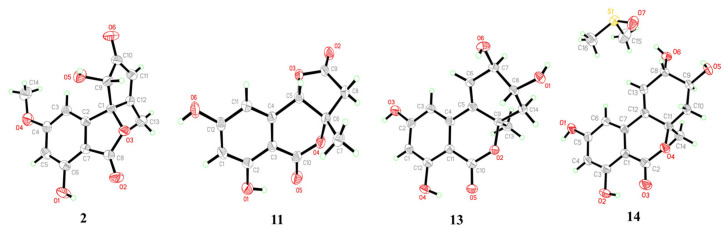
X-ray crystallographic structures of compounds **2**, **11**, **13**, and **14**.

**Figure 4:**
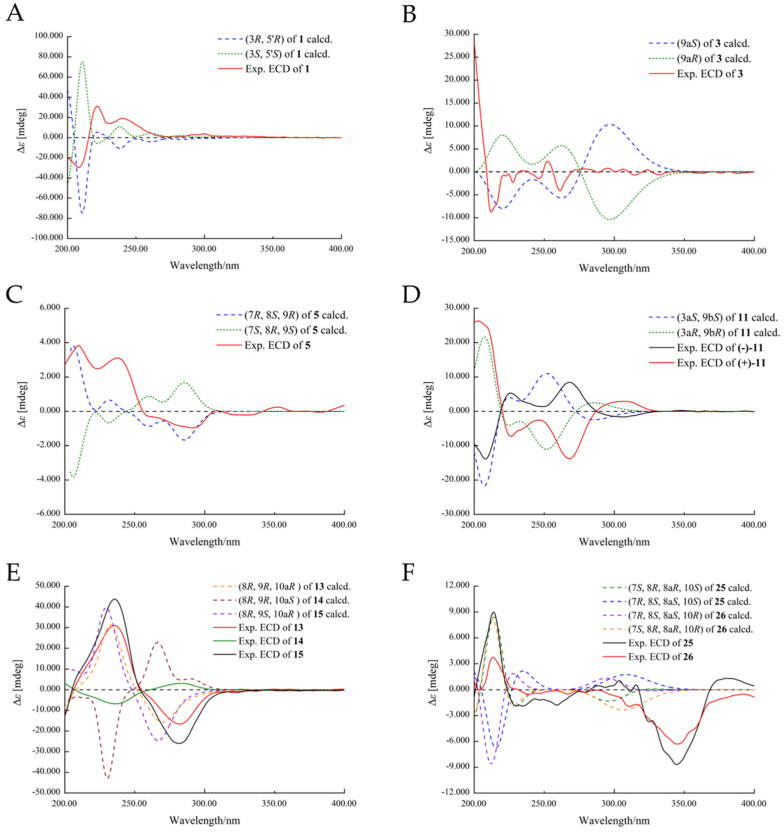
The experimental and calculated ECD spectra of **1**, **3**, **5**, **11**, **13**–**15**, **25**, and **26**.

The molecular formula of compound **1** was given as C_13_H_10_O_6_ from HRESIMS data with nine degrees of unsaturation. A comprehensive analysis of its ^1^H, ^13^C, and DEPT NMR spectra (Table 1) revealed great similarity to talaroflavone (**2**), a previously described compound isolated from the sponge-derived fungus *Alternaria* sp. F49 [19]. However, the signals of a methoxy group at *δ*_C/H_ 55.8/3.75 at C-5 in the NMR spectra of **2** were absent in those of **1**. In comparison to **2**, obvious upfield shifts for C-5 in **1** were detected. These differences suggested that compound **1** was a 5-demethylation derivative of **2**, which was further supported by the HMBC correlations of H-4/H-6 to C-5 (Figure 2). From a biosynthetic point of view, the stereoscopic configuration of **1** was assumed to be the same as that of **2**. After slow evaporation of the MeOH solvent, single crystals of **2** were obtained. The relative configuration of **2** was thus assigned by the result of a single-crystal X-ray diffraction experiment using Cu Kα radiation as 3S*, 5′S* (Figure 3). Accordingly, the relative configuration of **1** was also established as 3S*, 5′S*, the same as **2**. The absolute configuration of **1** was further studied by the time-dependent density functional (TDDFT) ECD calculation. The calculated ECD curve of (3*R*,5′*R*)-**1** agreed well with the experimental ECD curve of **1** (Figure 4), which ascertained compound **1** as (3*R*,5′*R*)-5-hydroxytalaroflavone.

The protonated molecular ion peak at *m/z* 261.0398 [M − H]^–^ in HRESIMS revealed the molecular formula of C_13_H_10_O_6_ for **3** with nine degrees of unsaturation. As shown in Table 1, the ^1^H NMR spectrum exhibited signals for two singlet aromatic protons (*δ*_H_ 3.26 and 2.94), one doublet methylene (*δ*_H_ 3.12 and 2.88), and one singlet methyl (*δ*_H_ 1.70). The ^13^C and DEPT NMR spectra data of **3** indicated the presence of 13 carbon signals, which were sorted into one methyl, one methylene, two methines, and nine quaternary carbons. Detailed analysis of the NMR data revealed that the structure of **3** was similar to that of compound **4**, an altenusin analog bearing a 6/6/5 tricyclic ring skeleton obtained from the mangrove endophytic fungus *Alternaria* sp. SK6YW3L [20]. However, resonances for a methoxy group of **4** were absent in the NMR spectra of **3**, which suggested that **3** was a 5-demethylated derivative of **4**. The above deduction was supported by HMBC correlation from H-4 to C-5 (Figure 2). The absolute configuration of **3** was established as 9a*S* based on the quantum chemical calculation of ECD (Figure 4) and nominated as talaroisochromenol A.

**Figure 5:**
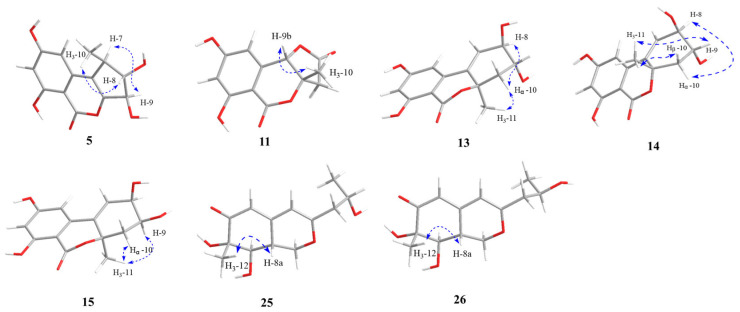
The NOESY correlations of compounds **5**, **11**, **13**–**15**, **25**, and **26**.

Compound **5** was afforded as a yellow oil, and the molecular formula of C_13_H_12_O_6_ for **5** was assigned by its positive HRESIMS. The NMR spectra of **5** were identical to those of **6**, a known altenusin derivative with a 6/6/5 tricyclic ring [20], except for the obvious differences in the chemical shifts of CH-8, CH-9, and CH_3_-10. The above observation suggested that **5** was a new epimer of **6** (epimeric at the C-9 position), which was further proved by the NOESY correlations from H-7 to H-9 and from H_3_-10 to H-8 (Figure 5). Hence, the absolute configuration of **5** was assigned as 7*R*, 8*S*, 9*R*, which was further confirmed by comparison of its calculated ECD for 7*R*, 8*S*, 9*R*-**5** with the measured ECD spectra (Figure 4). Thus, compound **5** was acknowledged as a new compound, namely talaroisochromenol B.

Compound **11** possessed a molecular formula of C_12_H_10_O_6_ as established from its HRESIMS spectrum, accounting for eight degrees of unsaturation. In the ^1^H NMR spectra, resonances for two phenolic hydroxyl groups (*δ*_H_ 11.08 and 10.99), two *meta*-substituted aromatic protons (*δ*_H_ 6.62, d, *J* = 2.2 Hz; *δ*_H_ 6.46, d, *J* = 2.2 Hz), one singlet oxymethine (*δ*_H_ 5.49), a chemically nonequivalent methylene (*δ*_H_ 3.26 and 2.94), and one singlet methyl (*δ*_H_ 1.51) were observed. The investigation of ^13^C NMR and HSQC spectral data displayed attribution signals of two lactone carbonyl groups, six aromatic carbons (including two methines and four quaternary carbons), one oxygenated quaternary carbon, one oxygenated methine, one methylene, and one methyl. These data demonstrated great similarity to those of **12**, a known polyketide yielded from an endolichenic fungus *Ulocladium* sp. [21], except for the absence of a methoxy group at C-8 (Table 2). Compared to **12**, the obvious upfield shift of C-8 in **11** was observed. Thus, **11** was determined as an 8-hydroxylated derivative of **12**.

The relative configuration of **11** was determined by the NOESY correlation between H-9b and H_3_-10 as 3aS*, 9aS* (Figure 5). The planar structure and relative configuration of **11** were further verified by an X-ray diffraction experiment (Figure 3). However, compound **11** was acquired as a racemate with an optical rotation value near zero, and no obvious Cotton effect was observed from the ECD spectrum. The chiral HPLC separation of **11** on a Chiralcel IG column (hexane/isopropanol = 80:20, flow rate of 1 mL/min) succeeded in separating two isomers (Figure S2). Finally, as shown in Figure 4, the ECD computation allowed the assignment of absolute configurations for two isomers as (+)-**11**-(3a*R*, 9a*R*) and (−)-**11**-(3a*S*, 9a*S*), respectively. Thus, **11** was named talaroisochromenol C.

Compounds **13**–**15** were originally isolated as yellow oils and owned the same molecular formula of C_14_H_14_O_6_ based on their positive HRESIMS data. The UV spectra of **13**–**15** showed similar characteristic absorptions for an isocoumarin chromophore at *λ*_max_ 242, 282, and 323 nm [22]. Subsequent interpretation of their NMR data (Table 3) revealed that compounds **13**–**15** were characterized as three new stereoisomers of a known analog 5-hydroxyaltenuene, which were isolated from an endophytic fungus *Penicillium* sp.FJ-1 of *Ceriops tagal* [22].

**Figure 6:**
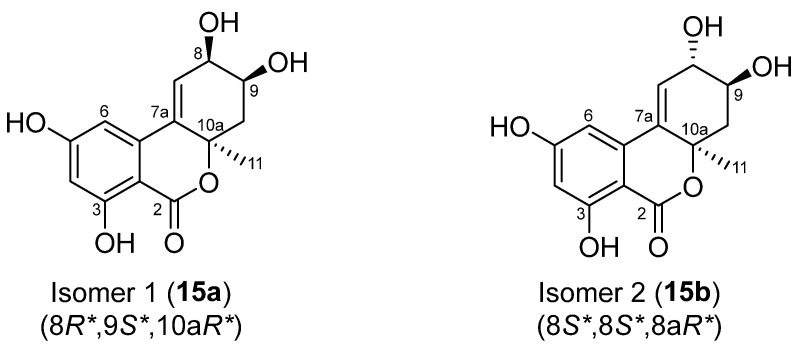
Two possible isomers of compound **15** for DP4+ probability analysis.

The relative configurations of **13**–**15** were assigned by NOESY, X-ray single-crystal diffraction experiment, or NMR calculations with DP4+ probability analysis. As shown in Figure 5, NOESY correlations from H*α*-10 to H-8 and H_3_-11 implied the *α*-orientation of CH_3_-11 and *β*-orientation of OH-8 in **13**. Meanwhile, NOESY correlations from H_3_-11 to H-9/H*β*-10 and from H-8 to H*α*-10 suggested a *syn* orientation of CH_3_-11 and H-9 and an opposite orientation of H-9 and H-8 in **14**. The relative configurations of **13** and **14** were further confirmed by single-crystal X-ray diffraction experimental data as (8R*,9R*, 10aR*)-**13** and (8S*,9S*,10aR*)-**14** (Figure 3). Moreover, NOESY correlations of **15** from H_3_-11 to H-9 and Hα-10 established its relative configuration of 9S*, 10aR*. The experimental ^1^H and ^13^C NMR data of **15** were compared with the calculated NMR data of **15a** and **15b** (two possible isomers of **15**, Figure 6) and matched well with those calculated for the isomer **15a** (8R*,9S*,10aR*) with a DP4+ probability of 100% (Table S16). The absolute configurations of **13**–**15** were determined by comparing the experimental ECD data with the calculated ones (Figure 4), suggesting **13**–**15** to be (8*R*,9*R*,10a*R*)-, (8*R*,9*R*, 10a*S*)- and (8*R*,9*S*,10a*R*)-5-hydroxyaltenuenes, respectively. Notably, the orientation of the methyl group at C-11 significantly determined the ECD Cotton effects observed for 5-hydroxyaltenuene. This was supported by the observation that compounds **13** and **15**, with 10a*R* absolute configurations, displayed positive Cotton effects at 236 nm and negative ones at 282 nm, in contrast to **14** with a 10a*S* absolute configuration. This was the first isolation of three new epimers of 5-hydroxyaltenuene from the fungus *Talaromyces* sp.

Compounds **25** and **26** were both obtained as yellow oily substances with similar chromatographic properties in the semipreparative HPLC separation [*t_R_* (**25**) = 14 min, *t_R_* (**26**) = 12 min]. The HRESIMS analysis displayed pseudomolecular ion peaks at *m/z* 255.1228 [M + H]^+^ (calcd for 255.1227) of **25** and at *m/z* 255.1226 [M + H]^+^ (calcd for 255.1227) of **26**, assigning their molecular formula both as C_13_H_18_O_5_ holding five degrees of unsaturation. Their UV spectra exhibited absorption maxima at 209 and 320 nm, revealing structural analogies between **25** and **26**. A detailed inspection of ^1^H and ^13^C NMR spectra of **25** and **26** (Table 4) prominently indicated practically identical signals, suggesting that they shared the same planar structural scaffold. Both ^1^H NMR spectral data of **25** and **26** exhibited characteristic resonances ascribed to two olefinic protons (represented for two trisubstituted double bands), three sp^3^-hybridized methines (including two oxygenated), two sp^3^-hybridized methylenes (including one chemically nonequivalent *O*-substituted), two methyl groups (as a singlet and a doublet), and three exchangeable protons. Analyses of their ^13^C, DEPT, and HSQC spectrum confirmed the presence of a carbonyl group, two pairs of olefin carbons, two oxygenated methines, one sp^3^-hybridized methine, two methylene groups, two methyls, and one quaternary carbon.

The ^1^H-^1^H COSY and HMBC correlations of **25** and **26** shown in Figure 2 established the same planar structure, which was similar to nemanecins A–C, three azaphilone analogs isolated from the culture broth of the fungus *Nemania* sp. BCC 30850 [23], indicating that **25** and **26** are a pair of stereoisomers. However, the signal of a singlet methyl in the ^1^H and ^13^C NMR spectra of nemanecins A–C were absent in those of **25** and **26**. Instead, signals for a 2-hydroxypropyl group were observed in the NMR spectra of **25** and **26**. The above inference was further confirmed by the key ^1^H-^1^H COSY correlation from H-10 to H-9 and H-11 as well as the observed HMBC correlations from H-9 to C-3, C-4, and C-10 and from H-11 to C-9 and C-10 (Figure 2). Thus, the planar structures of **25** and **26** were assigned.

The relative configurations of compounds **25** and **26** of C-7, C-8, and C-8a were both assigned as 7S*,8R*, and 8aR* based on the NOESY analysis and scalar coupling constant data (Figure 5). Both of them displayed a NOESY correlation from H_3_-10 to H-8a, which implied that H_3_-10 and H-8a were *co*-facial and arbitrarily assigned in *β*-orientation. The large coupling constant between H-8a and H-8 (*J* = 10.1 Hz) confirmed the *trans*-relationship of these protons.

**Figure 7:**
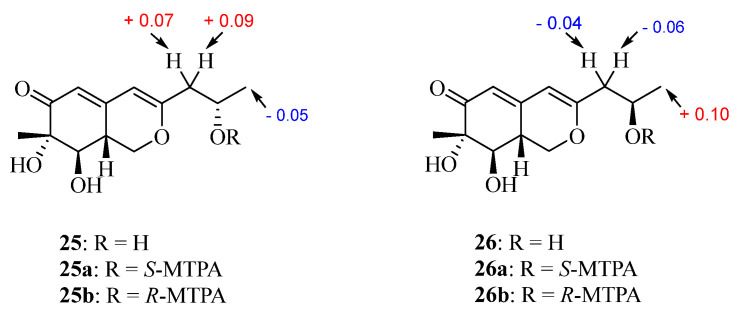
The Δ*δ* values [Δ*δ* = *δ_S_* − *δ_R_*] for the MTPA esters **25** and **26**.

The modified Mosher’s method using an NMR tube [24,25] was used to determine the absolute configuration of C-10 in **25** and **26**, leading to the assignments of *S*- for **25** and *R*-absolute configuration for **26** based on Δ*δ* values (Figure 7). ECD calculations were performed to further solve the absolution configurations of C-7, C-8, and C-8a in **25** and **26**. The experimental ECD spectra of **25** and **26** both exhibited a strong positive Cotton effect at 214 nm and a negative one at 345 nm, which was in good agreement with the calculated ECD curve of (7*S*,8*R*,8a*R*,10*S*)-**25** and (7*S*,8*R*,8a*R*,10*R*)-**26** (Figure 5). The results of ECD calculations indicated that the configuration of C-10 made no contribution to the ECD Cotton effects. Thus, the structures of compounds **25** and **26** were finally identified and named nemanecins D and E, respectively.

An analysis of the HRESIMS spectrum for *m/z* 316.9668 [M + H]^+^ (calcd for 316.9669) determined the molecular formula of compound **27** (yellow oil) as C_11_H_10_O_3_I requiring seven degrees of unsaturation. Its ^1^H and ^13^CNMR data (Table 5) were in good accord with the NMR spectral information of the known chromone derivative 2,5-dimethychromone [26], except that the signal of the methine of C-8 resonating at *δ*_C_ 100.4 in the NMR spectra of 2,5-dimethychromone was replaced by a deprotonated carbon resonating at *δ*_C_ 75.3 in those of **27**. The chemical shift of C-8 at *δ*_C_ 75.3 suggested the substitution by an iodine atom according to the previously reported compounds 2-iodo-5-methoxyphenol (*δ*_C_ 74.4) [27] and (*aS*)-6-iodofonsecinone A (*δ*_C_ 79.3) [28]. Thus, the structural assignment of **27** was assigned as 2,5-dimethy-8-iodochromone as shown in Figure 1, which was further confirmed by the HMBC correlations from H_3_-9 to C-2/C-3, H-3 to C-2/C-4a, and H-10 to C-4a/C-5/C-6 (Figure 2).

Compound **28** was obtained as a yellow oil, and its ^1^H and ^13^C NMR spectra data (Table 5) exactly matched those of the known compound 6-hydroxy-4-methoxycoumarin synthesized in the previous literature [18]. It is now isolated for the first time from a natural source. We supplemented its relevant NMR, MS, and UV data here.

**Figure 8:**
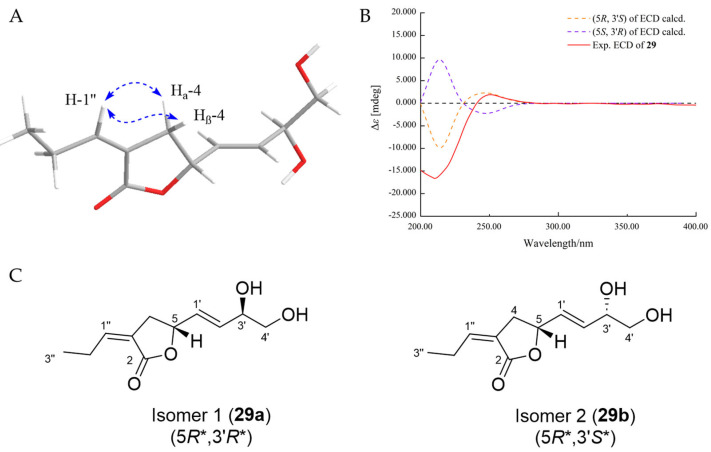
The NOESY correlations (**A**), experimental and calculated ECD spectra (**B**), and two possible isomers for DP4+ probability analysis (**C**) of **29**.

The HRESIMS data established the molecular formula of compound **29** as C_11_H_16_O_4_ with four degrees of unsaturation. As shown in Table 5, the ^1^H and ^13^C NMR spectrum displayed signals corresponding to two quaternary carbons (one lactone and one olefinic), five methines (three olefinic and one oxygenated), three methylenes (one oxygenated), and one methyl. The ^1^H-^1^H COSY correlations from H-4 to H-5 and HMBC correlations from H_2_-4 to C-2 and C-3 as well as H-5 to C-4 established a dihydrofuran-2(3*H*)-one moiety. The assignment for a spin coupling system of [=CHCH_2_CH_3_] was identified by ^1^H-^1^H COSY correlations from H-2” to H-1” and H-3”. Furthermore, a 3,4-dihydroxybut-1-en-1-yl group was assigned by ^1^H-^1^H COSY correlations from H-2′ to H-1 and H-3′, from H-3′ to H-4′ and 3′-OH, and from H-4′ to 4′-OH. The moiety of [=CHCH_2_CH_3_] spin system was attached to C-3 outside the lactone ring with regard to the HMBC correlations from H-1″ to C-2 and C-4 and from H-2″ to C-3. In addition, the 3,4-dihydroxybut-1-en-1-yl group was bonded to C-5, as proven by the ^1^H-^1^H COSY correlation from H-1′ to H-5 and HMBC correlations from H-1′ to C-4 and C-5 and from H-2′ to C-5. The *E*-configuration of double bonds Δ^1′, 2′^ was assigned based on the large coupling constants of *J* = 15.5 Hz, while the *cis* relation of Δ^3, 1″^ was ascertained by NOESY correlation between H_2_-4 and H-1″ (Figure 8A).

The relative configuration of **29** was determined by NMR calculation with DP4^+^ analysis. As a result, the experimental NMR data of **29** corresponded to the computed NMR data for (5R*,3′S*)-isomer **29b** (99.12% probability, Table S19 in the supplementary material). The absolute configuration of **29** was assigned by ECD calculation. The calculated ECD curves of (5*R*,3′*S*)-**29** coincided with the measured curve, which demonstrated the absolute configuration of **29** as 5*R*, 3′*S* (Figure 8B). Thus, the structure of **29** was established, and it was named talarofuranone A trivially_._

**Figure 9:**
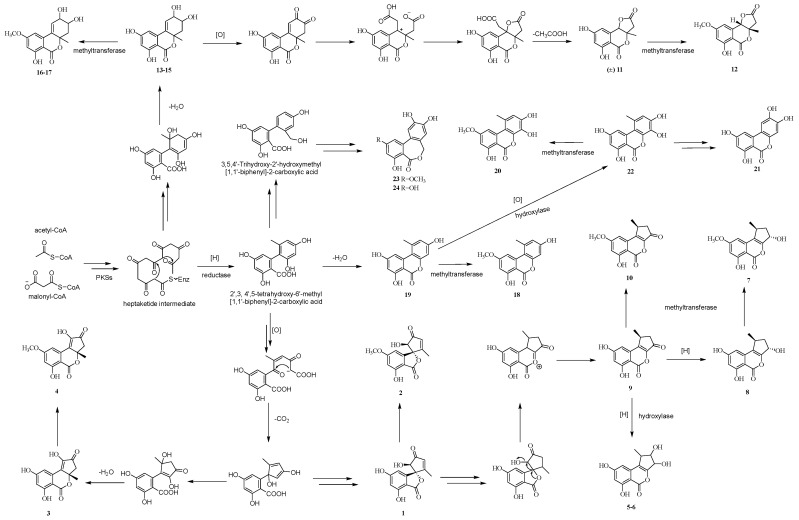
The possible biosynthetic routes of compounds **1**–**24**.

In addition, based on the comparison of their NMR and optical rotation data with those reported in the literature, the structures of the other sixteen polyketides were identified as phialophoriol (**7**) [29], (1*S*,3*S*)-2,3-dihydro-3,6,8-trihydroxy-1-methylcyclopenta[*c*][2]-benzopyran-5(1*H*)-one (**8**) [20], (*S*)-1,2-dihydro-6,8-dihydroxy-1-methylcyclo-penta[*c*][2]-benzopyran-3,5-dione (**9**) [20], (*S*)-1-deoxyrubralactone (**10**) [30], (8*R*,9*R*,10a*R*)-altenuene (**16**) [31], (8*S*,9*S*,10a*R*)-altenuene (**17**) [31], alternariol 9-*O*-methyl ether (**18**) [32,33], alternariol (**19**) [33,34], 3′-hydroxyalternariol-5-*O*-methyl ether (**20**) [34], 3-*O*-demethylaltenuisol (**21**) [35], 4-hydroxyalternariol (**22**) [36,37], alterlactone (**23**) [34], alteryulactone (**24**) [38], purpactin A (**30**) [39], penicillide (**31**) [39,40], and dehydroisopenicillide (**32**) [41]. Among them, compounds **1**–**24** are altenusin or alternariol derivatives with a similar polyketide origin. Based on the previously reported references, the possible biosynthetic routes of these polyketides are proposed here (Figure 9) [20,21,29,42,43,44,45]. All of them are derived from the heptaketide intermediate through the iterative condensation of acetyl-CoA and malonyl-CoA *via* PKSs, followed by a multi-step reaction of aldol-type cyclization, lactonization, methylation, hydroxylation, reduction, transmethylation, etc.

### 2.2. Biological Activity

Partially isolated compounds were evaluated for their in vitro antimicrobial activities against some pathogenic bacteria and fungi. Eleven bacterial strains including four human pathogens (methicillin-resistant *Staphylococcus aureus*, *Pseudomonas aeruginosa*, *Escherichia coli*, and *Klebsiella pneumonia*) and seven aquatic bacteria (*Vibrio alginolyticus*, *Aeromonas hydrophilia*, *Micrococcus luteus*, *Vibrio anguillarum*, *Vibrio parahaemolyticus*, *Vibrio vulnificus*, and *Vibrio harveyi*) together with six plant-pathogenic fungi (*Ceratobasidium cornigerum*, *Penicillium digitatum*, *Physalospora piricola*, *Valsa mali*, *Colletotrichum gloeosporioides*, and *Fusarium oxysporum*) were used as the tested strains. As shown in Table 6, Tables S1 and S2, eleven compounds, **7**, **9**–**10**, **18**–**22**, **24**, **30**, and **31**, exhibited comparable broad-spectrum antibiotic effects with MIC values ranging from 0.5 to 64 μg/mL. 

Notably, compound **10** displayed significant inhibitory activities against four bacteria, namely *E. coli*, *A. hydrophilia*, *V. parahaemolyticus*, and *V. harveyi*, with MIC values in the range of 0.5–1 μg/mL, better than or equivalent to the positive control chloramphenicol (MIC = 0.25–2 μg/mL). Compound **18** showed considerable antibacterial activities against human pathogen *E. coli* and aquatic pathogenic bacteria *V. parahaemolyticus* that were comparable to those of the positive control, chloramphenicol. Moreover, compounds **20**, **22**, and **24** displayed inhibitory effects on the growth of *P. aeruginosa* with *V. vulnificus*, *A. hydrophilia* with *V. parahaemolyticus*, and *A. hydrophilia*, respectively, with MIC values like those of chloramphenicol from 0.5 to 4 μg/mL. Unfortunately, all measured compounds did not show any significant antifungal activity at a concentration of 64 µg/mL.

The structure–activity relationship (SAR) for the above polyketides is discussed here. The addition of a ketonic carbonyl or methoxy group in the structures of methylcyclopenta[*c*][2]-benzopyran could improve their antibacterial effects (**10** vs. **7**–**9**). Then, a comparison of the inhibitory activities of compounds **18**–**24** indicated that their activities were influenced by the number and position of methyl, hydroxyl, and methoxy groups, as well as the expansion of the lactone ring. Furthermore, a correlation was observed between the acetylation of the side chain and antibacterial efficacy for penicillide-type compounds (**30** vs. **31**).

## 3. Materials and Methods

### 3.1. General Experimental Procedures

The general experimental procedures in this study were similar to those previously reported [6,14,15,46]. 

### 3.2. Fungal Material

The fungus strain CS-258 was isolated from the marine mussel sample collected from a cold seep in the northeast of the South China Sea in May 2020, and its strain identification was conducted by a BLAST search in GenBank. This strain sequence data had been uploaded to GenBank to obtain an accession number of No. PP065775. The strain was deposited at the Key Laboratory of Experimental Marine Biology, Institute of Oceanology, Chinese Academy of Sciences (IOCAS).

### 3.3. Fermentation, Extraction, and Isolation

The fresh mycelium of *Talaromyces* sp. CS-258 was cultured on potato dextrose broth medium for 3 days and then incubated in autoclaved 1 L Erlenmeyer flasks with rice culture medium (70 g rice, 0.2 g corn steep liquor, 0.5 g yeast extract, 0.3 g peptone, 0.6 g gourmet powder, and 100 mL seawater collected from the Huiquan Gulf of the Yellow Sea near the campus of IOCAS) for 30 days at room temperature. After incubation, the EtOAc crude extract was obtained by exhaustive concentration with MeOH and successive extraction with EtOAc/H_2_O four times. 

The crude extract was fractionated by silica gel column vacuum liquid chromatography (VLC) elution with increasing polarity gradient of petroleum ether (PE)/EtOAc and CH_2_Cl_2_/MeOH to yield nine fractions (Fr.A–Fr.I). Fr.E was further split by column chromatography (CC) using Sephadex LH-20 to produce three subfractions from E-1 to E-3. Fr.E-1 was rechromatographed over silica gel elution with a slow CH_2_Cl_2_/MeOH gradient from 200:1 to 20:1 to yield three mixtures of E.1.1–E.1.3. Then, mixture E.1.1 was followed by semipreparative HPLC separation (Elite ODS-BP column, 5 μm, 10 × 250 mm, MeOH-H_2_O, 75:25, 3 mL/min) to afford compounds **12** (6.0 mg, *t*_R_ 8 min) and **10** (10.2 mg, *t*_R_ 10 min). Mixtures of E.1.2 and E.1.3 were also subjected to semipreparative HPLC purification to yield **7** (3.3 mg, *t*_R_ 20 min, 70% MeOH-H_2_O), **30** (2.0 mg, *t*_R_ 18 min, 75% MeOH-H_2_O), and **31** (5.1 mg, *t*_R_ 28 min, 65% MeOH-H_2_O). Compound **4** (21.1 mg) was obtained via recrystallization from Fr.E-2. Another semipreparative HPLC collection with 75% MeOH-H_2_O was applied for Fr.E-3 to give compounds **18** (4.2 mg, *t*_R_ 22 min), **19** (3.2 mg, *t*_R_ 16 min), and **20** (4.0 mg, *t*_R_ 18 min).

Fr.F was subjected to reversed-phase CC using Lobar LiChroprep RP-18 from 10% to 90% MeOH-H_2_O to produce three subfractions, Fr.F-1 to F-3. Fr.F-1 was sequentially recrystallized to obtain compound **2** (100.0 mg). Fr.F-2 was subjected to Sephadex LH-20 CC to afford compound **24** (65.9 mg) and two mixed components. Then, the former component was purified by HPLC separation with MeOH-H_2_O (52:48) and identified as **8** (13.7 mg, *t*_R_ 25 min). The latter was subjected to preparative TLC (developing solvents: CH_2_Cl_2_−MeOH, 20:1) to obtain **27** (3.1 mg). Treated with successive CC on Sephadex LH-20 and HPLC with 58% MeOH-H_2_O, Fr.F-3 was found to produce compounds **9** (16.5 mg, recrystallization), **21** (15.3 mg, *t*_R_ 16 min), **22** (9.5 mg, *t*_R_ 18 min), and **23** (4.9 mg, recrystallization) totally. 

Fr.G was subjected to further CC over Lobar LiChroprep RP-18 in the MeOH−H_2_O solvent system and thus gave 6 subfractions, Fr.G-1 to Fr.G-6. Subsequently, compounds **25** (4.0 mg, *t*_R_ 14 min) and **26** (2.5 mg, *t*_R_ 12 min) were provided from Fr.G-1 by Sephadex LH-20 (MeOH) and semipreparative HPLC elution with 42% MeOH−H_2_O; meanwhile, compound **1** (11.0 mg, *t*_R_ 16 min) was obtained from another mixture of Fr.G-1 via HPLC in 30% MeOH−H_2_O. Fr.G-2 was subjected to a series of CC on Sephadex LH-20 and the HPLC separation system of 42% MeOH/H_2_O and 45% MeOH/H_2_O to afford **11** (6.7 mg, *t*_R_ 18 min) and **5** (24.6 mg, *t*_R_ 15 min). Fr.G-3 was further purified by Sephadex LH-20 and then by preparative TLC (developing solvents: CH_2_Cl_2_−MeOH, 15:1) and by HPLC with 80% MeOH/H_2_O to obtain compounds **13** (90.0 mg, *t*_R_ 27 min), **14** (14.6 mg, *t*_R_ 35 min), and **15** (4.8 mg, *t*_R_ 25 min). The remaining subfractions, Fr.G-4 to Fr.G-6, were partitioned using nearly the same method through CC over Sephadex LH-20, silica gel, and semipreparative HPLC to produce compounds **16** (5.5 mg, from Fr.G-4, *t*_R_ 10 min, 52% MeOH/H_2_O), **17** (5.0 mg, from Fr.G-6, *t*_R_ 28 min, 51% MeOH/H_2_O), and **6** (6.0 mg, from Fr.G-5).

After CC on reversed-phase RP-18 and Sephadex LH-20, Fr.I was fractionated and purified to give compound **29** (3.2 mg, by silica gel column and following Sephadex LH-20), **28** (2.0 mg, by preparative TLC), **3** (13.0 mg, by semipreparative HPLC collection of 46% MeOH-H_2_O at *t*_R_ 35 min), and **32** (2.4 mg, by preparative TLC and Sephadex LH-20).

### 3.4. Spectroscopic Data

*(3R,5*′*R)-5-hydroxytalaroflavone* (**1**): brown oil; ${\left[\alpha \right]}_{D}^{25}\;$+42.9 (*c* 0.44, MeOH); UV (MeOH, 0.20 mg/mL) *λ*_max_ (log *ε*) 216 (3.39), 259 (2.94), 297 (2.58) nm; ECD (0.20 mg/mL, MeOH) *λ*_max_ (Δ*ε*) 208 (−11.78), 222 (+12.43), 240 (+7.56) nm; ^1^H and ^13^C NMR data, see Table 1; (+)-HRESIMS *m/z* 263.055012 [M + H]^+^ (calcd for C_13_H_11_O_6_, 263.055014).

*Talaroisochromenol A* (**3**): brown oil; ${\left[\alpha \right]}_{D}^{25}\;$+105.9 (*c* 0.06, MeOH); UV (MeOH, 0.20 mg/mL) *λ*_max_ (log *ε*) 209 (3.13), 261 (2.92), 310 (2.64) nm; ECD (0.20 mg/mL, MeOH) *λ*_max_ (*Δε*) 213 (−3.47), 261 (−1.62) nm; ^1^H and ^13^C NMR data, see Table 1; (+)-HRESIMS *m/z* 261.0398 [M + H]^+^ (calcd for C_13_H_9_O_6_, 261.0405).

*Talaroisochromenol B* (**5**): yellow oil; ${\left[\alpha \right]}_{D}^{25}\;$+33.3 (*c* 0.24, MeOH); UV (MeOH, 0.20 mg/mL) 248 (3.08), 330 (2.18) nm; ECD (0.20 mg/mL, MeOH) *λ*_max_ (Δ*ε*) 210 (+1.54), 238 (+1.24), 291 (−0.38) nm; ^1^H and ^13^C NMR data, see Table 1; (+)-HRESIMS *m/z* 265.0714 [M + H]^+^ (calcd for C_13_H_13_O_6_, 265.0707).

*Talaroisochromenol C* (**11**): white solid; ${\left[\alpha \right]}_{D}^{25}\;$+4.3 (*c* 0.24, MeOH); UV (MeOH, 0.25 mg/mL) *λ*_max_ (log *ε*) 213 (3.20), 229 (2.96), 265 (2.68), 305 (2.56) nm; ^1^H and ^13^C NMR data, see Table 2; (+)-HRESIMS *m/z* 251.0556 [M + H]^+^ (calcd for C_12_H_11_O_6_, 251.0550).

(+)-(**11**): ${\left[\alpha \right]}_{D}^{25}\;$+60.0 (*c* 0.20, MeOH); ECD (0.30 mg/mL, MeOH) *λ*_max_ (Δ*ε*) 206 (+6.83), 226 (–1.93), 268 (−3.68) nm.

(−)-(**11**): ${\left[\alpha \right]}_{D}^{25}\;$−45.0 (*c* 0.20, MeOH); ECD (0.25 mg/mL, MeOH) *λ*_max_ (Δ*ε*) 208 (−4.41), 226 (+1.70), 268 (+2.69) nm.

*(8R,9R,10aR)-5-hydroxyaltenuene* (**13**): brown oil; ${\left[\alpha \right]}_{D}^{25}\;$−23.7 (*c* 0.30, MeOH); UV (MeOH, 0.25 mg/mL) *λ*_max_ (log *ε*) 242 (3.24), 282 (2.73), 323 (2.47) nm; ECD (0.25 mg/mL, MeOH) *λ*_max_ (Δ*ε*) 236 (+10.43), 282 (−5.59) nm; ^1^H and ^13^C NMR data, see Table 3; (+)-HRESIMS *m/z* 279.0870 [M + H]^+^ (calcd for C_14_H_15_O_6_, 279.0863).

*(8R,9R,10aS)-5-hydroxyaltenuene* (**14**): brown oil; ${\left[\alpha \right]}_{D}^{25}\;$+43.6 (*c* 0.18, MeOH); UV (MeOH, 0.20 mg/mL) *λ*_max_ (log *ε*) 244 (3.34), 282 (2.81), 325 (2.49) nm; ECD (0.20 mg/mL, MeOH) *λ*_max_ (Δ*ε*) 237 (−2.85), 284 (+1.31) nm; ^1^H and ^13^C NMR data, see Table 3; (+)-HRESIMS *m/z* 279.0869 [M + H]^+^ (calcd for C_14_H_15_O_6_, 279.0863).

*(8R,9S,10aR)-5-hydroxyaltenuene* (**15**): brown oil; ${\left[\alpha \right]}_{D}^{25}\;$−85.7 (*c* 0.21, MeOH); UV (MeOH, 0.30 mg/mL) *λ*_max_ (log *ε*) 244 (3.17), 282 (2.64), 325 (2.32) nm; ECD (0.30 mg/mL, MeOH) *λ*_max_ (Δ*ε*) 236 (+12.30), 282 (−7.29) nm; ^1^H and ^13^C NMR data, see Table 3; (+)-HRESIMS *m/z* 279.0861 [M + H]^+^ (calcd for C_14_H_15_O_6_, 279.0863).

*Nemanecin D* (**25**): yellow oil; ${\left[\alpha \right]}_{D}^{25}\;$−11.5 (*c* 0.26, MeOH); UV (MeOH, 0.40 mg/mL) *λ*_max_ (log *ε*) 209 (2.98), 320 (3.10) nm; ECD (0.40 mg/mL, MeOH) *λ*_max_ (Δ*ε*) 214 (+1.73), 345 (−1.67) nm; ^1^H and ^13^C NMR data, see Table 4; (+)-HRESIMS *m/z* 255.1228 [M + H]^+^ (calcd for C_13_H_19_O_5_, 255.1227).

(*S*)-MTPA Ester (**25a**): ^1^H NMR (Figure S65, selected signals, pyridine-*d*_5_, 500 MHz) *δ*_H_: 5.60 (1H, m, H-10), 2.63 (1H, dd, H-9*_α_*), 2.60 (1H, dd, H-9*_β_*), 1.31 (3H, d, H-11); ^1^H-^1^H COSY and HRMS spectra, see Figures S66 and S67.

(*R*)-MTPA Ester (**25b**): ^1^H NMR (Figure S68, selected signals, pyridine-*d*_5_, 500 MHz) *δ*_H_: 5.51 (1H, m, H-10), 2.54 (2H, m, H-9), 1.36 (3H, d, H-11); ^1^H-^1^H COSY and HRMS spectra, see Figures S69 and S70.

*Nemanecin E* (**26**): yellow oil; ${\left[\alpha \right]}_{D}^{25}\;$−48.0 (*c* 0.40, MeOH); UV (MeOH, 0.25 mg/mL) *λ*_max_ (log *ε*) 209 (2.89), 319 (2.76) nm; ECD (0.40 mg/mL, MeOH) *λ*_max_ (Δ*ε*) 214 (+0.70), 345 (−1.18) nm; ^1^H and ^13^C NMR data, see Table 4; (+)-HRESIMS *m/z* 255.1226 [M + H]^+^ (calcd for C_13_H_19_O_5_, 255.1227).

(*S*)-MTPA Ester (**26a**): ^1^H NMR (Figure S79, selected signals, pyridine-*d*_5_, 500 MHz) *δ*_H_: 5.58 (1H, m, H-10), 2.62 (1H, dd, H-9*_α_*), 2.50 (1H, dd, H-9*_β_*), 1.38 (3H, d, H-11); ^1^H-^1^H COSY and HRMS spectra, see Figures S80 and S81.

(*R*)-MTPA Ester (**26b**): ^1^H NMR (Figure S82, selected signals, pyridine-*d*_5_, 500 MHz) *δ*_H_: 5.53 (1H, m, H-10), 2.68 (1H, dd, H-13*_α_*), 2.54 (1H, dd, H-9*_β_*), 1.28 (3H, d, H-15); ^1^H-^1^H COSY and HRMS spectra, see Figures S83 and S84.

*2,5-dimethy-8-iodochromone* (**27**): yellow oil; UV (MeOH, 0.05 mg/mL) *λ*_max_ (log *ε*) 210 (3.56), 247 (3.52), 296 (3.19) nm; ^1^H and ^13^C NMR data, see Table 5; (+)-HRESIMS *m/z* 316.9668 [M + H]^+^ (calcd for C_11_H_10_O_3_I, 316.9669).

*6-hydroxy-4-methoxycoumarin* (**28**): yellow oil; UV (MeOH, 0.05 mg/mL) *λ*_max_ (log *ε*) 222 (3.33), 270 (2.96), 331 (2.67) nm; ^1^H and ^13^C NMR data, see Table 5; (+)-HRESIMS *m/z* 193.0493 [M + H]^+^ (calcd for C_10_H_9_O_4_, 193.0495).

*Talarofurolactone A* (**29**): yellow oil; ${\left[\alpha \right]}_{D}^{25}$−110.3 (*c* 0.15, MeOH); UV (MeOH, 0.25 mg/mL) *λ*_max_ (log *ε*) 220 (3.01) nm; ECD (0.25 mg/mL, MeOH) *λ*_max_ (Δ*ε*) 211 (−4.27) nm; ^1^H and ^13^C NMR data, see Table 5; (+)-HRESIMS *m/z* 213.1122 [M + H]^+^ (calcd for C_11_H_17_O_4_, 213.1121), *m/z* 230.1389 [M + NH_4_]^+^ (calcd for C_11_H_20_O_4_N, 230.1387), *m/z* 235.0942 [M + Na]^+^ (calcd for C_11_H_16_O_4_Na, 235.0941).

### 3.5. X-ray Crystallographic Analysis of Compounds ***2***, ***11***, ***13***, and ***14***

Compounds **2**, **11**, **13**, and **14** were dissolved in MeOH and crystallized after slow evaporation in a refrigerator. All X-ray single-crystal diffraction data were recorded at 293(2) K on an Agilent Xcalibur Eos Gemini CCD plate diffractometer equipped with graphite monochromatic CuKα radiation. The data were revised for absorption with the program SADABS [47]. Direct methods of the SHELXTL software and subsequent refinement via full-matrix least-squares difference Fourier techniques were applied to solve the structures accurately [48,49]. Additional refinements for non-hydrogen, H, and O atoms were carried out as previously reported [9,14]. All crystallographic data have been deposited in the Cambridge Crystallographic Data Centre (CCDC) and can be obtained on application to the director free of charge. 

*Crystal data for* ***2****:* m.p. 224.0–248.0 °C, C_14_H_12_O_6_, fw = 276.24, monoclinic, space group *C2/c*, unit cell dimensions *a* = 23.8978(11) Å, *b* = 6.9420(3) Å, *c* = 14.9300(7) Å, V = 2467.83(19) Å^3^, *α* = 90^◦^, *β* = 94.895(2)^◦^, *γ* = 90^◦^, *Z* = 8, *d*_calcd_ = 1.487 mg/m^3^, crystal size 0.16 × 0.15 × 0.14 mm^3^, *μ* = (CuKα) 1.002 mm^−1^, *F*(000) = 1152.0. Independent reflections: 2260 [*R*_int_ = 0.0427, *R*_sigma_= 0.0248]. The final indices gave *R_1_* = 0.0405 and *wR_2_* = 0.1032 [*I* > 2*σ*(*I*)]. CCDC number was deposited as 2327879.

*Crystal data for **1******1****:* m.p. 181.3–184.0 °C, C_12_H_10_O_6_, fw = 250.20, triclinic, space group *P-1*, unit cell dimensions *a* = 7.300(2) Å, *b* = 7.4441(2) Å, *c* = 9.9268(2) Å, V = 516.92(2) Å^3^, *α* = 70.6080(10)^◦^, *β* = 86.7490(10)^◦^, *γ* = 87.6150(10)^◦^, *Z* = 2, *d*_calcd_ = 1.607 mg/m^3^, crystal size 0.16 × 0.15 × 0.14 mm^3^, *μ* = (CuKα) 1.126 mm^−1^, *F*(000) = 260.0. Independent reflections: 1869 [*R*_int_ = 0.0291, *R*_sigma_= 0.0280]. The final indices gave *R_1_* = 0.0347 and *wR_2_* = 0.0996 [*I* > 2*σ*(*I*)]. CCDC number was deposited as 2327875.

*Crystal data for* ***13****:* m.p. 244.1–247.2 °C, C_14_H_14_O_6_, fw = 278.25, monoclinic, space group *P2_1_/n*, unit cell dimensions *a* = 9.0780(2) Å, *b* = 6.90080(10) Å, *c* = 19.2001(4) Å, V = 1202.71(4) Å^3^, *α* = 90^◦^, *β* = 90.6920(10)^◦^, *γ* = 90^◦^, *Z* = 4, *d*_calcd_ = 1.537 mg/m^3^, crystal size 0.18 × 0.16 × 0.15 mm^3^, *μ* = (CuKα) 1.028 mm^−1^, *F*(000) = 584.0. Independent reflections: 2185 [*R*_int_ = 0.0319, *R*_sigma_= 0.0248]. The final indices gave *R_1_* = 0.0328 and *wR_2_* = 0.0873 [*I* > 2*σ*(*I*)]. CCDC number was deposited as 2327876.

*Crystal data for* ***14****:* m.p. 273.0–278.0 °C, C_14_H_14_O_6_ +(CH_3_)_2_SO, fw = 356.38, monoclinic, space group *P2_1_/c*, unit cell dimensions *a* = 8.8828(3) Å, *b* = 20.8250(7) Å, *c* = 8.9113(3) Å, V = 1595.86(9) Å^3^, *α* = 90^◦^, *β* = 104.511(2)^◦^, *γ* = 90^◦^, *Z* = 2, *d*_calcd_ = 1.483 mg/m^3^, crystal size 0.16 × 0.15 × 0.12 mm^3^, *μ* = (CuKα) 2.143 mm^−1^, *F*(000) = 752.0. Independent reflections: 2917 [*R*_int_ = 0.0489, *R*_sigma_ = 0.0318]. The final indices gave *R_1_* = 0.0315 and *wR_2_* = 0.0877 [*I* > 2*σ*(*I*)]. CCDC number was deposited as 2327877.

### 3.6. Antimicrobial Activity Assay

The antimicrobial evaluations against four human pathogens (methicillin-resistant *S. aureus*, *P. aeruginosa*, *E. coli*, and *K. pneumonia*) and seven aquatic bacteria (*V. alginolyticus*, *A. hydrophilia*, *M. luteus*, *V. anguillarum*, *V. parahaemolyticus*, *V. vulnificus*, and *V. harveyi*) as well as six plant-pathogenic fungi (*C. cornigerum*, *P. digitatum*, *P. piricola*, *V. mali*, *C. gloeosporioides* and *F. oxysporum*) were carried out in a 96-well microplate as described in our previous reports [6,46,50,51]. These microbial strains were provided by the Institute of Oceanology, Chinese Academy of Sciences, by purchase or isolation. Chloramphenicol and amphotericin B served as positive controls against bacteria and fungi, respectively, while DMSO was treated as the negative control. All measurements at various concentrations were performed in triplicate.

### 3.7. Details of Computational Methods

The computational methods for ECD calculation and DP4+ probability analysis were similar to those in the previous papers from our group [6,10,51], with some modifications detailed below. Molecular mechanics using the MM^+^ method was carried out for conformational searches in HyperChem software (Version 8.0, Hypercube, Inc., Gainesville, FL, USA). The energy-minimized conformers were generated and further optimized using DFT calculations at the B3LYP/6-31G(d) level in Gaussian 09 software (Version D.01; Gaussian, Inc.: Wallingford, CT, USA). Frequency calculations were carried out at the same level of theory to confirm the absence of imaginary frequencies and to obtain thermal corrections to the Gibbs free energies. These obtained conformers were subjected to ECD calculations using the TDDFT method at the CAM-B3LYP/TZVP, BH&HLYP/TZVP, or PBE0/TZVP level. The solvent effects of MeOH were evaluated at the same DFT level using the self-consistent reaction field (SCRF) method with the polarizable continuum model (PCM). The ECD spectrum was generated by the SpecDis program and finally drawn using Origin Pro 8.5 software [52,53,54,55,56,57]. The NMR shielding tensors were calculated by the DFT method at the mPW1PW91\6-31+G(d) PCM level in DMSO and then weighted according to Boltzmann’s distribution. GIAO (gauge-independent atomic orbital) NMR chemical calculations were performed using an equation described previously. The theoretical shielding tensors (^1^H and ^13^C) and experimental chemical shifts were finally analyzed and compared using DP4+ probability [58,59,60].

### 3.8. The Modified Mosher’s Method

The modified Mosher’s method was conducted in NMR tubes following the details described in the previous papers [25,61].

## 4. Conclusions

In this study, the chemical investigation of cold-seep-derived fungus *Talaromyces* sp. CS-258 has resulted in the isolation and identification of 32 fungal polyketides including 11 new compounds and 1 new naturally isolated metabolite. Notably, a pair of enantiomers ((±)-**11**) and two groups of diastereoisomers (**13**–**15** and **25**/**26**) were obtained, the isolation of which presented challenges due to their difficulty in separation from each other. Furthermore, compound **27** was identified as a rare occurring natural iodo-chromone analog. The plausible biogenetic pathways of compounds **1**–**24**, which belong to altenusin or alternariol families with polyketide origin bearing variable tricyclic ring skeletons, were proposed. The results of antimicrobial activities displayed that 11 polyketides exhibited broad-spectrum antibiotic effects against human and aquatic pathogenic bacteria. Among them, compounds **10** and **18** exhibited potent inhibition against *E. coli*, *A. hydrophilia*, and *V. parahaemolyticus*, while **22** and **24** effectively suppressed the growth of *A. hydrophilia*. These findings highlight the cold-seep-derived fungus *Talaromyces* sp. CS-258 as a promising source for bioactive metabolites, especially for fungal polyketides, with potential applications as antibiotic agents in medicinal development and agriculture.

## Figures and Tables

**Table 1:** ^1^H and ^13^C NMR spectroscopic data of **1**, **3,** and **5** in DMSO-*d_6_* (*δ* in ppm, *J* in Hz).

No.	1		3		5	
1		166.9 C				
2				168.2 C		166.9 C
2a				99.1 C ^1^		97.8 C
3		91.4 C		165.0 C		166.3 C
3a		149.9 C				
4	5.76, d (1.6)	100.8 CH	6.44, s	103.2 CH	6.33, s	101.4 CH
5		164.6 C		163.9 C		163.8 C
6	6.34, s	103.3 CH	6.98, s	106.9 CH	6.37, s	102.1 CH
6a				133.1 C		137.2 C
7a		103.5 C		130.1 C		118.1 C
7		158.7 C		150.8 C ^1^	6.89, dd (6.9, 2.6)	42.0 CH
8	1.76, s	12.9 CH_3_		197.0 C	3.67, m	83.3 CH
9			3.12, d (18.0) 2.88, d (18.0)	46.8 CH_2_	4.47, d (2.4)	78.1 CH
9a				81.4 C		153.1 C
10			1.70, s	27.3 CH_3_	1.29, d (6.9)	18.5 CH_3_
2′		169.2 C				
3′	6.36, s	129.8 CH				
4′		200.1 C				
5′	4.61, s	78.2 CH				

^1^ Observed in HMBC spectrum.

**Table 2:** ^1^H and ^13^C NMR spectroscopic data of **11** in DMSO-*d_6_* (*δ* in ppm, *J* in Hz).

No.	11	
2		173.2 C
3	3.26, d (17.6) 2.94, d (17.6)	42.2 CH_2_
3a		85.9 C
5		166.0 C
5a		97.8 C
6		163.5 C
7	6.46, d (2.2)	104.3 CH
8		165.2 C
9	6.62, d (2.2)	110.9 CH
9a		133.3 C
9b	5.49, s	76.9 CH
10	1.51, s	19.9 CH_3_
6-OH	10.99, s	
8-OH	11.08, s	

**Table 3:** ^1^H and ^13^C NMR spectroscopic data of **13**–**15** in DMSO-*d_6_* (*δ* in ppm, *J* in Hz).

No.	13		14		15	
2		168.3 C		167.8 C		167.7 C
2a		98.8 C		98.3 C		98.1 C
3		163.1 C		163.3 C		163.2 C
4	6.29, s	102.3 CH	6.26, d (2.1)	102.6 CH	6.28, overlap	102.7 CH
5		165.1 C		165.5 C		165.8 C
6	6.53, s	103.4 CH	6.47, d (2.1)	103.3 CH	6.53, d (2.2)	103.8 CH
6a		139.4 C		137.7 C		138.2 C
7a		132.2 C		131.4 C		133.5 C
7	6.14, d (3.1)	130.3 CH	6.05, d (2.5)	130.3 CH	6.28, overlap	127.8 CH
8	3.95, t (4.0)	69.7 CH	4.07, dd (7.9, 2.5)	72.5 CH	4.08, t (4.6)	64.9 CH
9	3.68, dt (7.9, 3.9)	68.8 CH	3.59, ddd (12.0, 7.9, 3.7)	70.2 CH	3.66, dt (12.3, 3.7)	65.4 CH
10	*β* 2.24, dd (14.0, 3.5) *α* 1.93, dd (14.5, 8.1)	38.7 CH_2_	*β* 2.14, dd (12.0, 3.7) *α* 2.01, t (12.3)	43.5 CH_2_	*β* 2.21, t (12.0) *α* 1.90, dd (11.6, 3.3)	38.4 CH_2_
10a		80.9 C		82.0 C		81.6 C
11	1.45, s	27.4 CH_3_	1.48, s	26.2 CH_3_	1.43, s	25.8 CH_3_
3-OH	11.22, s		11.25, s		11.28, s	

**Table 4:** ^1^H and ^13^C NMR spectroscopic data of **25**–**26** in DMSO-*d_6_* (*δ* in ppm, *J* in Hz).

No.	25		26	
1	4.56, dd (10.7, 5.4) 3.69, dd (13.4, 10.7)	68.6 CH_2_	4.56, dd (10.7, 5.4) 3.67, m	68.6 CH_2_
3		165.0 C		164.9 C
4	5.58, s	101.8 CH	5.59, s	101.7 CH
4a		151.0 C		150.9 C
5	5.54, d (2.2)	114.4 CH	5.55, d (2.1)	114.3 CH
6		200.6 C		200.5 C
7		77.1 C		77.0 C
8	3.45, dd (10.1, 4.5)	73.7 CH	3.45, dd (10.1, 4.6)	73.6 CH
8a	2.58, m	37.7 CH	2.59, m	37.7 CH
9	2.25, m	44.2 CH_2_	2.32, dd (14.0, 6.8) 2.17, dd (14.0, 6.2)	44.1 CH_2_
10	3.86, dq (12.1, 6.2)	64.1 CH	3.88, m	64.0 CH
11	1.07, d (6.2)	18.6 CH_3_	1.07, d (6.2)	18.6 CH_3_
12	1.10, s	23.4 CH_3_	1.10, s	23.3 CH_3_
7-OH	4.93, s		4.92, s	
8-OH	5.33, d (4.5)		5.31, d (4.6)	
10-OH	4.69, d (5.0)		4.69, d (5.2)	

**Table 5:** ^1^H and ^13^C NMR spectroscopic data of **27**–**29** in DMSO-*d_6_* (*δ* in ppm, *J* in Hz).

No.	27		28		29	
2		160.9 C		161.9 C		170.5 C
3	5.75, s	109.8 CH	5.85, s	90.3 CH		126.5 C
4		176.9 C		165.7 C	3.09, dd (17.5, 8.6) 2.55, dd (17.0, 3.0)	31.5 CH_2_
4a		108.7 C ^1^		115.6 C		
5		138.4 C	7.10, d (2.8)	106.8 CH	5.03, q (7.1)	77.4 CH
6	6.19, s	120.0 CH		153.7 C		
7		159.1 C	7.06, dd (8.8, 2.9)	120.7 CH		
7a						
8		75.3 C	7.25, d (8.8)	117.5 CH		
8a		- ^2^		146.0 C		
9	2.22, s	19.1 CH_3_				
10	2.48, s	20.4 CH_3_				
1′					5.73, m	128.7 CH
2′					5.83, m	135.4 CH
3′					3.98, brs	71.6 CH
4′					3.30, m	66.2 CH_2_
5′						
1″					6.54, tt (7.6, 3.0)	141.4 CH
2″					2.16, m	23.3 CH_2_
3″					1.02, t (7.5)	12.9 CH_3_
4-OMe			3.99, s	57.0 CH_3_		
6-OH			9.90, brs			
3′-OH					4.92, brs	
4′-OH					4.64, brs	

^1^ Observed in HMBC spectrum, ^2^ missed signal.

**Table 6:** Antibacterial activity of isolated compounds (MIC, μg/mL).

Bacteria	Compounds											
	Chloramphenicol	7	9	10	18	19	20	21	22	24	30	31
MRSA	8	64	-	-	-	64	64	-	-	-	64	-
PA	4	16	-	32	-	16	4	-	-	-	32	32
EC	0.25	32	32	0.5	0.5	8	4	32	32	8	4	64
KP	8	64	64	64	-	64	64	-	64	64	64	64
VAl	1	-	64	-	-	32	64	64	32	-	-	64
AH	0.5	32	32	0.5	4	8	4	-	0.5	0.5	8	-
ML	1	32	32	-	-	8	4	-	-	-	8	-
VAn	1	64	-	-	-	8	64	-	-	-	16	-
VP	1	32	32	1	2	8	4	64	0.5	64	32	32
VV	4	-	32	16	-	8	4	-	-	-	-	-
VH	2	32	16	0.5	32	16	8	-	32	64	8	-

* MRSA: methicillin-resistant *S. aureus*, PA: *P. aeruginosa*, EC: *E. coli*, KP: *K. pneumonia*, VAl: *V. alginolyticus*, AH: *A. hydrophilia*, ML: *M. luteus*, VAn: *V. anguillarum*, VP: *V. parahaemolyticus*, VV: *V. vulnificus*, VH: *V. harveyi*. -: no activity.

## Data Availability

The data are included in the article and the Supplementary Material.

## Supplementary Materials

The following supporting information can be downloaded at https://www.mdpi.com/article/10.3390/md22050204/s1. Figures S1–Figures S120: Chemical structures, HRESIMS, UV, 1D and 2D NMR spectra of new compounds; 1D/2D NMR and HRMS data for the modified Mosher’s product of compounds **25** and **26**, the chiral HPLC separation of compound **11**; antibacterial and antifungal activities of isolated compounds and optimized low-energy conformers for ECD calculations. Tables S1–S23: Energy analysis for optimized conformers of new compounds, the DP4^+^ probability analysis and calculated shielding tensors of each conformer for the candidate isomers of **15** and **29**, and the experimental as well as calculated chemical shifts of **15** and **29**.
