# Supplementary material for: Antibacterial Polyketides from the Deep-Sea Cold-Seep-Derived Fungus Talaromyces sp. CS-258
Source: Mar Drugs. 2024 Apr 28;22(5):204. doi: 10.3390/md22050204 (PMC11122946; doi:10.3390/md22050204)
Supplement: Supplementary file 1 [file marinedrugs-22-00204-s001.zip › marinedrugs-2964917-supplementary.pdf]

Supplementary Information

Antibacterial Polyketides from the Deep-Sea Cold-Seep-Derived Fungus *Talaromyces* sp. CS-258

Zhenger Wu [1,2], Xiao-Ming Li [1,2], Sui-Qun Yang [1,2], Bin-Gui Wang [1,2,3,*] and Xin Li [1,2,3,*]

[1] CAS and Shandong Province Key Laboratory of Experimental Marine Biology, Institute of Oceanology, Chinese Academy of Sciences, Nanhai Road 7, Qingdao 266071, China

[2] University of Chinese Academy of Sciences, Yuquan Road 19A, Beijing 100049, China

[3] Laboratory for Marine Biology and Biotechnology, Qingdao Marine Science and Technology Center, Wenhai Road 1, Qingdao 266237, China

* Correspondence: wangbg@ms.qdio.ac.cn (B.-G.W.); lixin@qdio.ac.cn (X.L.)

Content

Figure S1. Chemical structure of compounds **1**–**32** 7
Figure S2. The Chiral HPLC separation of compound **11** with Chiralcel IG column (hexane: isopropanol= 80:20, flow rate of 1 mL/minutes) 7
Table S1. The antibacterial activity of isolated compounds (MIC, μg/mL) 8
Table S2. The antifungal activity of isolated compounds 8
Figure S3. HRESIMS spectrum of (3*R*,5'*R*)-5-hydroxytalaroflavone (**1**) 9
Figure S4. ^{1}H NMR spectrum (500 MHz, DMSO) of (3*R*,5'*R*)-5-hydroxytalaroflavone (**1**) 9
Figure S5. ^{13}C NMR spectrum (125 MHz, DMSO) of (3*R*,5'*R*)-5-hydroxytalaroflavone (**1**) 10
Figure S6. ^{1}H-^{1}H COSY spectrum of (3*R*,5'*R*)-5-hydroxytalaroflavone (**1**) 10
Figure S7. HSQC spectrum of (3*R*,5'*R*)-5-hydroxytalaroflavone (**1**) 11
Figure S8. HMBC spectrum of (3*R*,5'*R*)-5-hydroxytalaroflavone (**1**) 11
Figure S9. NOESY spectrum of (3*R*,5'*R*)-5-hydroxytalaroflavone (**1**) 12
Figure S10. UV spectrum of (3*R*,5'*R*)-5-hydroxytalaroflavone (**1**) 12
Figure S11. HRESIMS spectrum of talaroisochromenol A (**3**) 13
Figure S12. ^{1}H NMR spectrum (400 MHz, DMSO) of talaroisochromenol A (**3**) 13
Figure S13. ^{13}C NMR spectrum (100 MHz, DMSO) of talaroisochromenol A (**3**) 14
Figure S14. ^{1}H-^{1}H COSY spectrum of talaroisochromenol A (**3**) 14
Figure S15. HSQC spectrum of talaroisochromenol A (**3**) 15
Figure S16. HMBC spectrum of talaroisochromenol A (**3**) 15
Figure S17. UV spectrum of talaroisochromenol A (**3**) 16
Figure S18. HRESIMS spectrum of talaroisochromenol B (**5**) 16
Figure S19. ^{1}H NMR spectrum (500 MHz, DMSO) of talaroisochromenol B (**5**) 17
Figure S20. ^{13}C NMR spectrum (125 MHz, DMSO) of talaroisochromenol B (**5**) 17
Figure S21. ^{1}H-^{1}H COSY spectrum of talaroisochromenol B (**5**) 18
Figure S22. HSQC spectrum of talaroisochromenol B (**5**) 18
Figure S23. HMBC spectrum of talaroisochromenol B (**5**) 19
Figure S24. NOESY spectrum of talaroisochromenol B (**5**) 19
Figure S25. UV spectrum of talaroisochromenol B (**5**) 20
Figure S26. HRESIMS spectrum of talaroisochromenol C (**11**) 20
Figure S27. ^{1}H NMR spectrum (500 MHz, DMSO) of talaroisochromenol C (**11**) 21
Figure S28. ^{13}C NMR spectrum (125 MHz, DMSO) of talaroisochromenol C (**11**) 21
Figure S29. ^{1}H-^{1}H COSY spectrum of talaroisochromenol C (**11**) 22
Figure S30. HSQC spectrum of talaroisochromenol C (**11**) 22

Figure S31. HMBC spectrum of talaroisochromenol C (**11**) 23
Figure S32. NOESY spectrum of talaroisochromenol C (**11**)..23
Figure S33. UV spectrum of talaroisochromenol C (**11**) 24
Figure S34. HRESIMS spectrum of (8*R*,9*R*,10a*R*)-5-hydroxyaltenuene (**13**) 24
Figure S35. ^{1}H NMR spectrum (500 MHz, DMSO) of (8*R*,9*R*,10a*R*)-5-hydroxyaltenuene (**13**) ...25
Figure S36. ^{13}C NMR spectrum (125 MHz, DMSO) of (8*R*,9*R*,10a*R*)-5-hydroxyaltenuene (**13**) ..25
Figure S37. ^{1}H-^{1}H COSY spectrum of (8*R*,9*R*,10a*R*)-5-hydroxyaltenuene (**13**) 26
Figure S38. HSQC spectrum of (8*R*,9*R*,10a*R*)-5-hydroxyaltenuene (**13**).......................................26
Figure S39. HMBC spectrum of (8*R*,9*R*,10a*R*)-5-hydroxyaltenuene (**13**).....................................27
Figure S40. NOESY spectrum of (8*R*,9*R*,10a*R*)-5-hydroxyaltenuene (**13**) 27
Figure S41. UV spectrum of (8*R*,9*R*,10a*R*)-5-hydroxyaltenuene (**13**)...28
Figure S42. HRESIMS spectrum of (8*R*,9*R*,10a*S*)-5-hydroxyaltenuene (**14**)...............................28
Figure S43. ^{1}H NMR spectrum (500 MHz, DMSO) of (8*R*,9*R*,10a*S*)-5-hydroxyaltenuene (**14**)....29
Figure S44. ^{13}C NMR spectrum (125 MHz, DMSO) of (8*R*,9*R*,10a*S*)-5-hydroxyaltenuene (**14**) ..29
Figure S45. ^{1}H-^{1}H COSY spectrum of (8*R*,9*R*,10a*S*)-5-hydroxyaltenuene (**14**)............................30
Figure S46. HSQC spectrum of (8*R*,9*R*,10a*S*)-5-hydroxyaltenuene (**14**) 30
Figure S47. HMBC spectrum of (8*R*,9*R*,10a*S*)-5-hydroxyaltenuene (**14**) 31
Figure S48. NOESY spectrum of (8*R*,9*R*,10a*S*)-5-hydroxyaltenuene (**14**)....................................31
Figure S49. UV spectrum of (8*R*,9*R*,10a*S*)-5-hydroxyaltenuene (**14**) 32
Figure S50. HRESIMS spectrum of (8*R*,9*S*,10a*R*)-5-hydroxyaltenuene (**15**)................................32
Figure S51. ^{1}H NMR spectrum (500 MHz, DMSO) of (8*R*,9*S*,10a*R*)-5-hydroxyaltenuene (**15**)....33
Figure S52. ^{13}C NMR spectrum (125 MHz, DMSO) of (8*R*,9*S*,10a*R*)-5-hydroxyaltenuene (**15**) ..33
Figure S53. ^{1}H-^{1}H COSY spectrum of (8*R*,9*S*,10a*R*)-5-hydroxyaltenuene (**15**)............................34
Figure S54. HSQC spectrum of (8*R*,9*S*,10a*R*)-5-hydroxyaltenuene (**15**) 34
Figure S55. HMBC spectrum of (8*R*,9*S*,10a*R*)-5-hydroxyaltenuene (**15**) 35
Figure S56. NOESY spectrum of (8*R*,9*S*,10a*R*)-5-hydroxyaltenuene (**15**)....................................35
Figure S57. UV spectrum of (8*R*,9*S*,10a*R*)-5-hydroxyaltenuene (**15**) 36
Figure S58. HRESIMS spectrum of nemanecin D (**25**) 36
Figure S59. ^{1}H NMR spectrum (500 MHz, DMSO) of nemanecin D (**25**)...................................37
Figure S60. ^{13}C NMR spectrum (125 MHz, DMSO) of nemanecin D (**25**)..................................37
Figure S61. ^{1}H-^{1}H COSY spectrum of nemanecin D (**25**) 38
Figure S62. HSQC spectrum of nemanecin D (**25**)...38
Figure S63. HMBC spectrum of nemanecin D (**25**)...39
Figure S64. NOESY spectrum of nemanecin D (**25**) 39
Figure S65. ^{1}H NMR spectrum of (*S*)-MTPA ester (**25a**)...40

Figure S66. ^{1}H-^{1}H COSY spectrum of (*S*)-MTPA ester (**25a**)..40
Figure S67. HRMS spectrum of (*S*)-MTPA ester (**25a**)...41
Figure S68. ^{1}H NMR spectrum of (*R*)-MTPA ester (**25b**)...41
Figure S69. ^{1}H-^{1}H COSY spectrum of (*R*)-MTPA ester (**25b**)..42
Figure S70. HRMS spectrum of (*R*)-MTPA ester (**25b**)..42
Figure S71. UV spectrum of nemanecin D (**25**)...43
Figure S72. HRESIMS spectrum of nemanecin E (**26**) 43
Figure S73. ^{1}H NMR spectrum (500 MHz, DMSO) of nemanecin E (**26**) 44
Figure S74. ^{13}C NMR spectrum (125 MHz, DMSO) of nemanecin E (**26**) 44
Figure S75. ^{1}H-^{1}H COSY spectrum of nemanecin E (**26**) 45
Figure S76. HSQC spectrum of nemanecin E (**26**) 45
Figure S77. HMBC spectrum of nemanecin E (**26**)...46
Figure S78. NOESY spectrum of nemanecin E (**26**) 46
Figure S79. ^{1}H NMR spectrum of (*S*)-MTPA Ester (**26a**)..47
Figure S80. ^{1}H-^{1}H COSY spectrum of (*S*)-MTPA Ester (**26a**)...47
Figure S81. HRMS spectrum of (*S*)-MTPA Ester (**26a**)..48
Figure S82. ^{1}H NMR spectrum of (*R*)-MTPA Ester (**26b**) 48
Figure S83. ^{1}H-^{1}H COSY spectrum of (*R*)-MTPA Ester (**26b**) 49
Figure S84. HRMS spectrum of (*R*)-MTPA Ester (**26b**) 49
Figure S85. UV spectrum of nemanecin E (**26**)...50
Figure S86. HRESIMS spectrum of 2,5-dimethy-8-iodochromone (**27**) 50
Figure S87. ^{1}H NMR spectrum (500 MHz, DMSO) of 2,5-dimethy-8-iodochromone (**27**)...........51
Figure S88. ^{13}C NMR spectrum (125 MHz, DMSO) of 2,5-dimethy-8-iodochromone (**27**)..........51
Figure S89. ^{1}H-^{1}H COSY spectrum of 2,5-dimethy-8-iodochromone (**27**) 52
Figure S90. HSQC spectrum of 2,5-dimethy-8-iodochromone (**27**)..52
Figure S91. HMBC spectrum of 2,5-dimethy-8-iodochromone (**27**)..53
Figure S92. UV spectrum of 2,5-dimethy-8-iodochromone (**27**)...53
Figure S93. HRESIMS spectrum of 6-hydroxy-4-methoxycoumarin (**28**)...................................54
Figure S94. ^{1}H NMR spectrum (400 MHz, DMSO) of 6-hydroxy-4-methoxycoumarin (**28**)........54
Figure S95. ^{13}C NMR spectrum (100 MHz, DMSO) of 6-hydroxy-4-methoxycoumarin (**28**).......55
Figure S96. ^{1}H-^{1}H COSY spectrum of 6-hydroxy-4-methoxycoumarin (**28**)...............................55
Figure S97. HSQC spectrum of 6-hydroxy-4-methoxycoumarin (**28**)...56
Figure S98. HMBC spectrum of 6-hydroxy-4-methoxycoumarin (**28**)...56
Figure S100. HRESIMS spectrum of talarofurolactone A (**29**) 57
Figure S101. ^{1}H NMR spectrum (500 MHz, DMSO) of talarofurolactone A (**29**).........................58

Figure S102. ^{13}C NMR spectrum (125 MHz, DMSO) of talarofurolactone A (**29**)........................58
Figure S103. ^{1}H-^{1}H COSY spectrum of talarofurolactone A (**29**)..59
Figure S104. HSQC spectrum of talarofurolactone A (**29**)..59
Figure S105. HMBC spectrum of talarofurolactone A (**20**)...60
Figure S106. NOESY spectrum of talarofurolactone A (**29**)...60
Figure S107. UV spectrum of talarofurolactone A (**29**)..61
Table S3. Energy analysis for the conformers of (3*R*,5'*R*)-5-hydroxytalaroflavone (**1**) 61
Figure S108. B3LYP/6-31G(d) optimized low-energy conformers of (3*R*,5'*R*)-5-hydroxytalaroflavone (**1**)..61
Table S4. Energy analysis for the conformers of (9a*S*)-talaroisochromenol A (**3**)........................61
Figure S109. B3LYP/6-31G(d) optimized low-energy conformers of (9a*S*)-talaroisochromenol A (**3**)..62
Table S5. Energy analysis for the conformers of (7*R*,8*S*,9*R*)-talaroisochromenol B (**5**) 62
Figure S110. B3LYP/6-31G(d) optimized low-energy conformers of (7*R*,8*S*,9*R*)-talaroisochromenol B (**5**) 63
Table S6. Energy analysis for the conformers of (3a*S*, 9b*S*)-talaroisochromenol C (**11**) 63
Figure S111. B3LYP/6-31G(d) optimized low-energy conformers of (3a*S*, 9b*S*)-talaroisochromenol C (**11**) 63
Table S7. Energy analysis for the conformers of (8*R*,9*R*,10a*R*)-5-hydroxyaltenuene (**13**)............63
Figure S112. B3LYP/6-31G(d) optimized low-energy conformers of (8*R*,9*R*,10a*R*)-5-hydroxyaltenuene (**13**) 64
Table S8. Energy analysis for the conformers of (8*S*,9*S*,10a*R*)-5-hydroxyaltenuene (*ent*-**14**) 64
Figure S113. B3LYP/6-31G(d) optimized low-energy conformers of (8*S*,9*S*,10a*R*)-5-hydroxyaltenuene (*ent*-**14**) 64
Table S9. Energy analysis for the conformers of (8*R*,9*S*,10a*R*)-5-hydroxyaltenuene (**15**) 65
Figure S114. B3LYP/6-31G(d) optimized low-energy conformers of (8*R*,9*S*,10a*R*)-5-hydroxyaltenuene (**15**) 65
Table S10. Energy analysis for the conformers of (7*S*,8*R*,8a*R*,10*S*)-nemanecin D (**25a**)...............65
Figure S115. B3LYP/6-31G(d) optimized low-energy conformers of (7*S*,8*R*,8a*R*,10*S*)-nemanecin D (**25a**)...66
Table S11. Energy analysis for the conformers of (7*R*,8*S*,8a*S*,10*S*)-nemanecin D (**25b**) 66
Figure S116. B3LYP/6-31G(d) optimized low-energy conformers of (7*R*,8*S*,8a*S*,10*S*)-nemanecin D (**25b**)...67
Table S12. Energy analysis for the conformers of (7*R*,8*S*,8a*S*,10*R*)-nemanecin E (**26a**) 67

Figure S117. B3LYP/6-31G(d) optimized low-energy conformers of (7*R*,8*S*,8a*S*,10*R*)-nemanecin E (**26a**) 68
Table S13**.** Energy analysis for conformers of (7*S*,8*R*,8a*R*,10*R*)-nemanecin E (**26b**) 68
Figure S118. B3LYP/6-31G(d) optimized low-energy conformers of (7*S*,8*R*,8a*R*,10*R*)-nemanecin E (**26b**) 69
Table S14**.** Energy analysis for the conformers of (5*R*,3'*S*)-talarofurolactone A (**29a**) 69
Figure S119. B3LYP/6-31G(d) optimized low-energy conformers of (5*R*,3'*S*)-talarofurolactone A (**29a**)..70
Table S15**.** Energy analysis for the conformers of (5*S*,3'*R*)-talarofurolactone A (**29b**) 70
Figure S120. B3LYP/6-31G(d) optimized low-energy conformers of (5*S*,3'*R*)-talarofurolactone A (**29b**)..71
Table S16**.** DP4$^+$ probability analysis of **15** (mPW1PW91/6-31+G (d, p) level)............................71
Table S17**.** The experimental and calculated chemical shifts (DP4+) of **15** 72
Table S18**.** The calculated shielding tensors of each conformer for isomer 1 (8*R**,9*S**,10a*R**-**15**) 73
Table S19**.** The calculated shielding tensors of each conformer for isomer 2 (8*S**,9*S**,10a*R**-**15**)73
Table S20**.** DP4$^+$ probability analysis of **29** (mPW1PW91/6-31+G(d, p) level).............................74
Table S21**.** The experimental and calculated chemical shifts (DP4+) of **29** 75
Table S22**.** The calculated shielding tensors of each conformer for isomer 1 (5*R**,3'*R**-**29**).........76
Table S23**.** The calculated shielding tensors of each conformer for isomer 2 (5*R**,3'*S**-**29**). 76

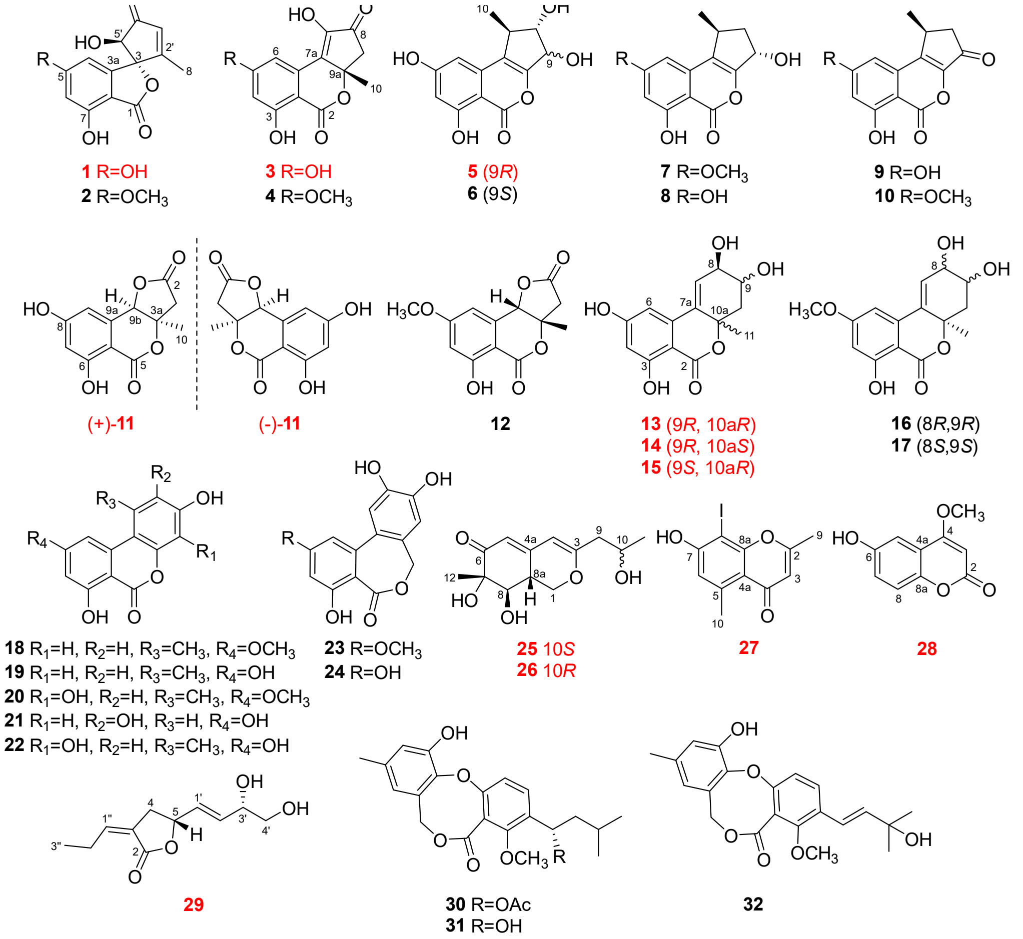

Figure S1. Chemical structure of compounds **1**–**32**

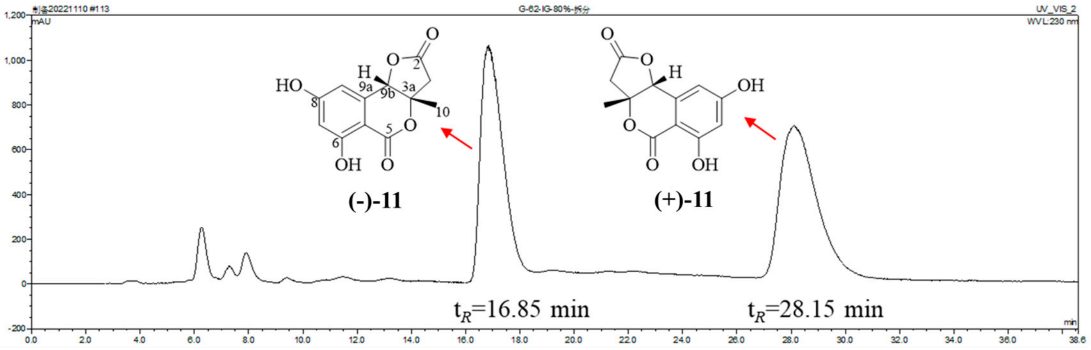

Figure S2. The Chiral HPLC separation of compound **11** with Chiralcel IG column (hexane: isopropanol= 80:20, flow rate of 1 mL/minutes)

Table S1. The antibacterial activity of isolated compounds (MIC, μg/mL)

Bacteria	Compounds										
	chloramphenicol	**1**	**2**	**4**	**5**	**7**	**8**	**9**	**10**	**11**	**12**	**13**
MRSA	8	-	-	-	-	64	-	-	-	-	-	-
PA	4	-	-	-	-	16	-	-	32	-	-	-
EC	0.25	-	-	-	-	32	-	32	0.5	-	-	-
KP	8	-	-	-	-	64	-	64	64	-	-	-
VAl	1	-	-	-	-	-	-	64	-	-	-	-
AH	0.5	-	-	-	-	32	-	32	0.5	-	-	-
ML	1	-	-	-	-	32	-	32	-	-	-	-
VAn	1	-	-	-	-	64	-	-	-	-	-	-
VP	1	-	-	-	-	32	-	32	1	-	-	-
VV	4	-	-	-	-	-	-	32	16	-	-	-
VH	2	-	-	-	-	32	-	16	0.5	-	-	-

Bacteria	Compounds													
	chloramphenicol	**14**	**15**	**16**	**17**	**18**	**19**	**20**	**21**	**22**	**23**	**24**	**30**	**31**
MRSA	8	-	-	-	-	-	64	64	-	-	-	-	64	-
PA	4	-	-	-	-	-	16	4	-	-	-	-	32	32
EC	0.25	-	-	-	-	0.5	8	4	32	32	-	8	4	64
KP	8	-	-	-	-	-	64	64	-	64	-	64	64	64
VAl	1	-	-	-	-	-	32	64	64	32	-	-	-	64
AH	0.5	-	-	-	-	4	8	4	-	0.5	-	0.5	8	-
ML	1	-	-	-	-	-	8	4	-	-	-	-	8	-
VAn	1	-	-	-	-	-	8	64	-	-	-	-	16	-
VP	1	-	-	-	-	2	8	4	64	0.5	-	64	32	32
VV	4	-	-	-	-	-	8	4	-	-	-	-	-	-
VH	2	-	-	-	-	32	16	8	-	32	-	64	8	-

Table S2. The antifungal activity of isolated compounds

Fungi	Compounds												
	amphotericin B	**1**	**2**	**4**	**5**	**7**	**8**	**9**	**10**	**11**	**12**	**13**	**14**
C. cornigerum	< 64	-	-	-	-	-	-	-	-	-	-	-	-
P. digitatum	< 64	-	-	-	-	-	-	-	-	-	-	-	-
P. piricola	< 64	-	-	-	-	-	-	-	-	-	-	-	-
V. mali	< 64	-	-	-	-	-	-	-	-	-	-	-	-
C. gloeosporioides	< 64	-	-	-	-	-	-	-	-	-	-	-	-
F. oxysporum	< 64	-	-	-	-	-	-	-	-	-	-	-	-

Fungi	Compounds											
	amphotericin B	**15**	**16**	**17**	**18**	**19**	**20**	**21**	**22**	**23**	**24**	**30**	**31**
C. cornigerum	< 64	-	-	-	-	-	-	-	-	-	-	-	-
P. digitatum	< 64	-	-	-	-	-	-	-	-	-	-	-	-
P. piricola	< 64	-	-	-	-	-	-	-	-	-	-	-	-
V. mali	< 64	-	-	-	-	-	-	-	-	-	-	-	-
C. gloeosporioides	< 64	-	-	-	-	-	-	-	-	-	-	-	-
F. oxysporum	< 64	-	-	-	-	-	-	-	-	-	-	-	-

-: no activity.

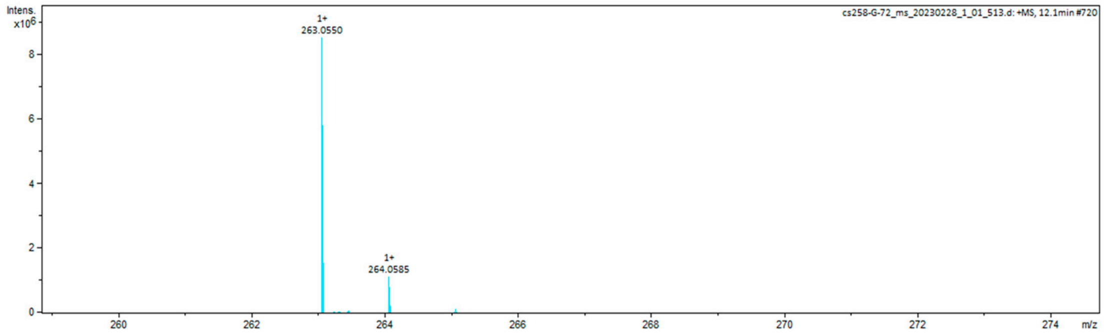

Meas. m/z	z	Ion Formula	Score	m/z	err [mDa]	err [ppm]	mSigma	rdb	e⁻Conf	N-Rule	Adduct	Sum Formula
263.055012	1	$C_{13}H_{11}O_6$	100.00	263.055014	0.0	0.0	8.9	8.5	even	ok	M+H	$C_{13}H_{10}O_6$

Figure S3. HRESIMS spectrum of (3*R*,5'*R*)-5-hydroxytalaroflavone (**1**)

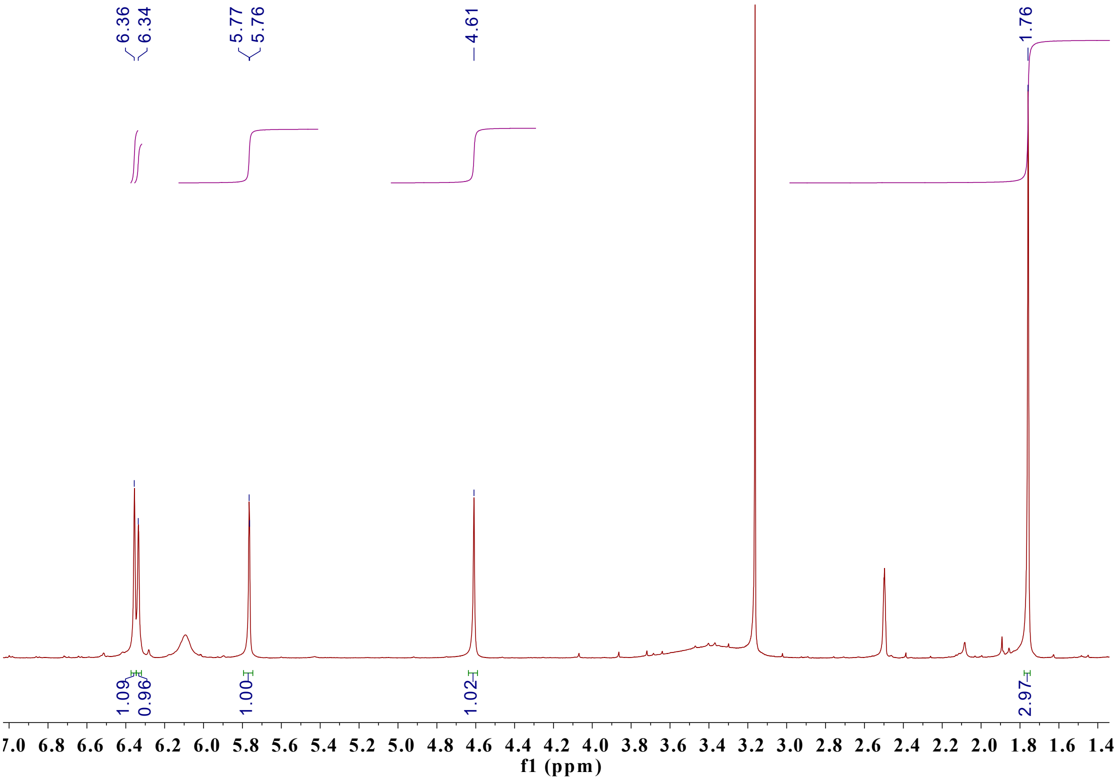

Figure S4. ^{1}H NMR spectrum (500 MHz, DMSO) of (3*R*,5'*R*)-5-hydroxytalaroflavone (**1**)

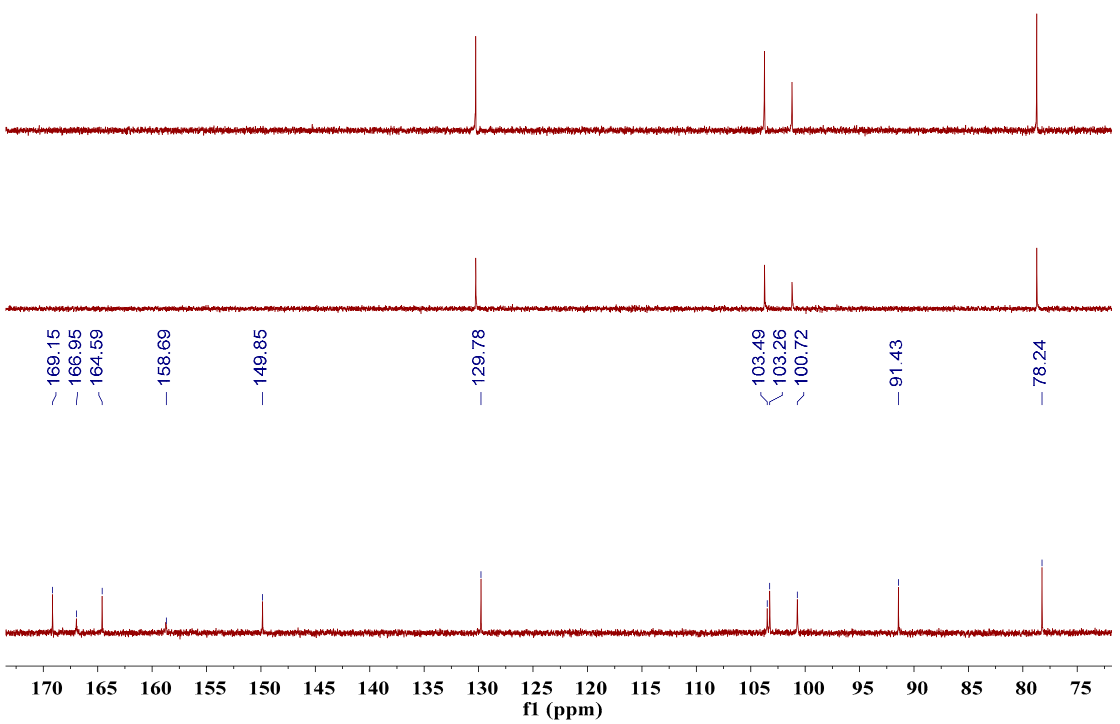

Figure S5. ^{13}C NMR spectrum (125 MHz, DMSO) of (3*R*,5'*R*)-5-hydroxytalaroflavone (**1**)

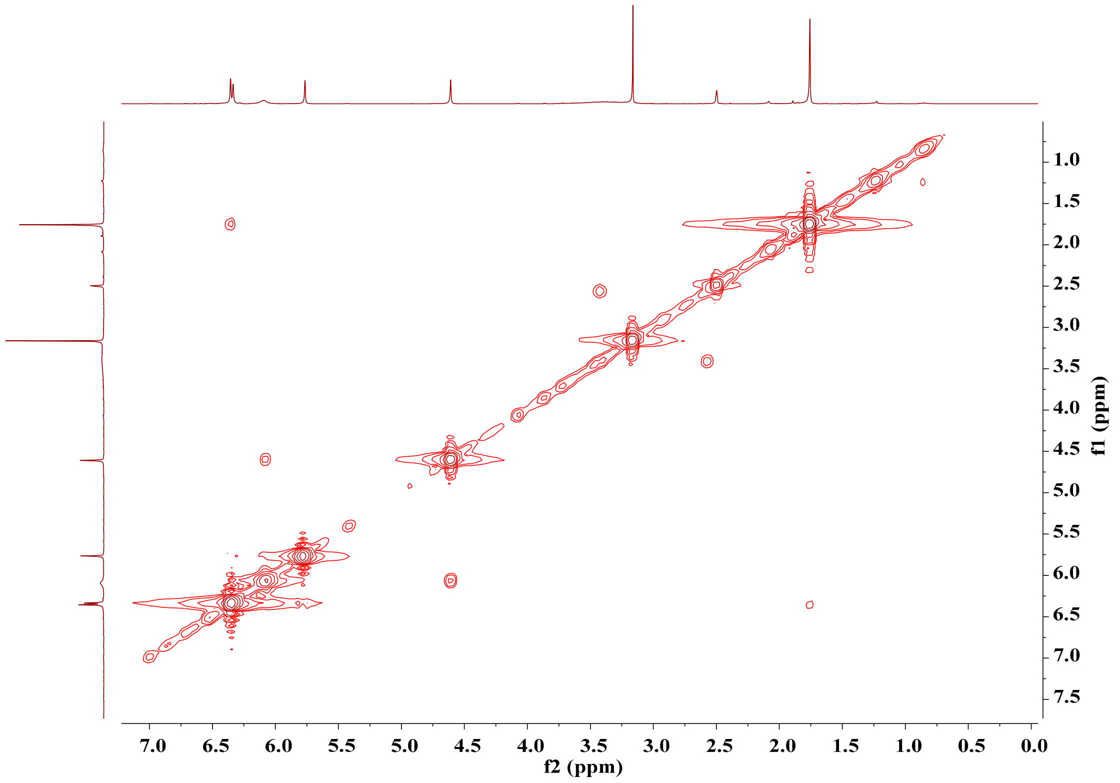

Figure S6. ^{1}H-^{1}H COSY spectrum of (3*R*,5'*R*)-5-hydroxytalaroflavone (**1**)

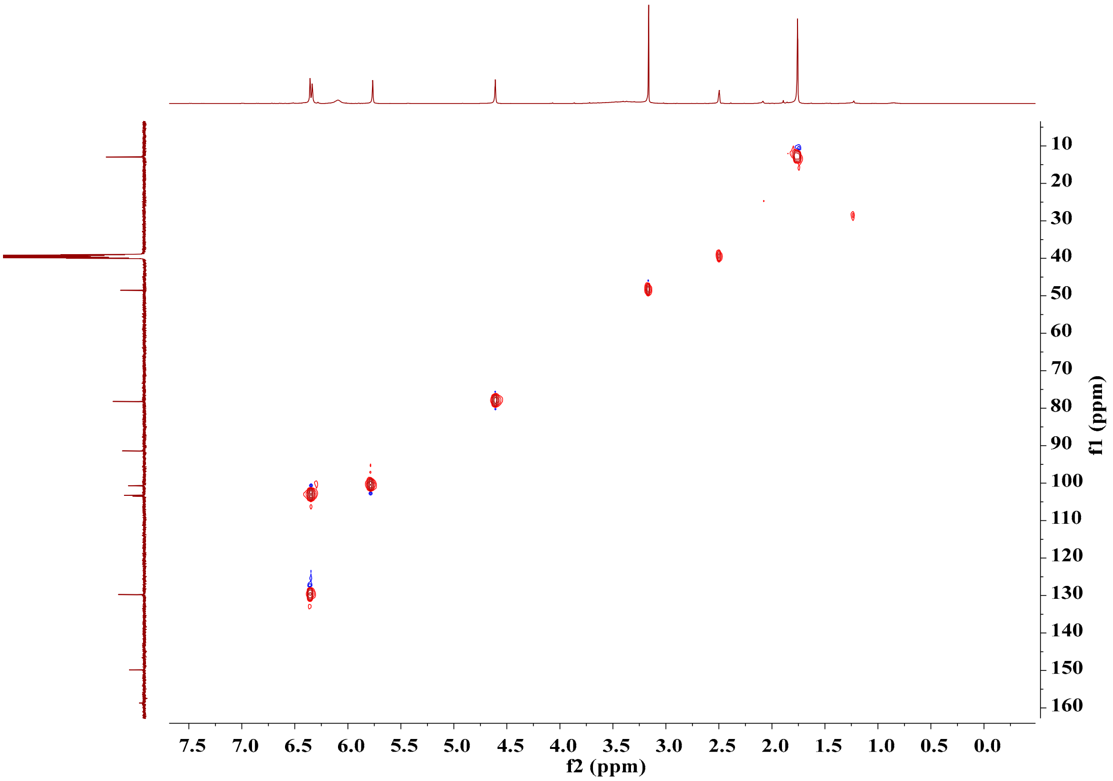

Figure S7. HSQC spectrum of (3*R*,5'*R*)-5-hydroxytalaroflavone (**1**)

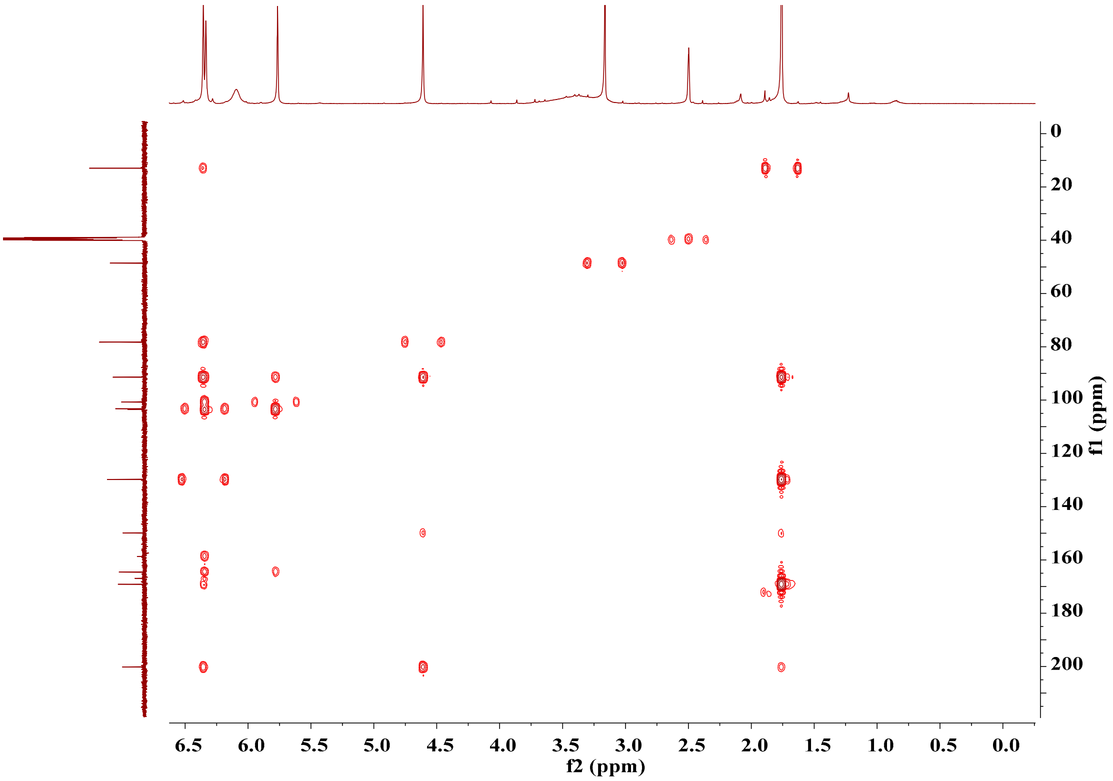

Figure S8. HMBC spectrum of (3*R*,5'*R*)-5-hydroxytalaroflavone (**1**)

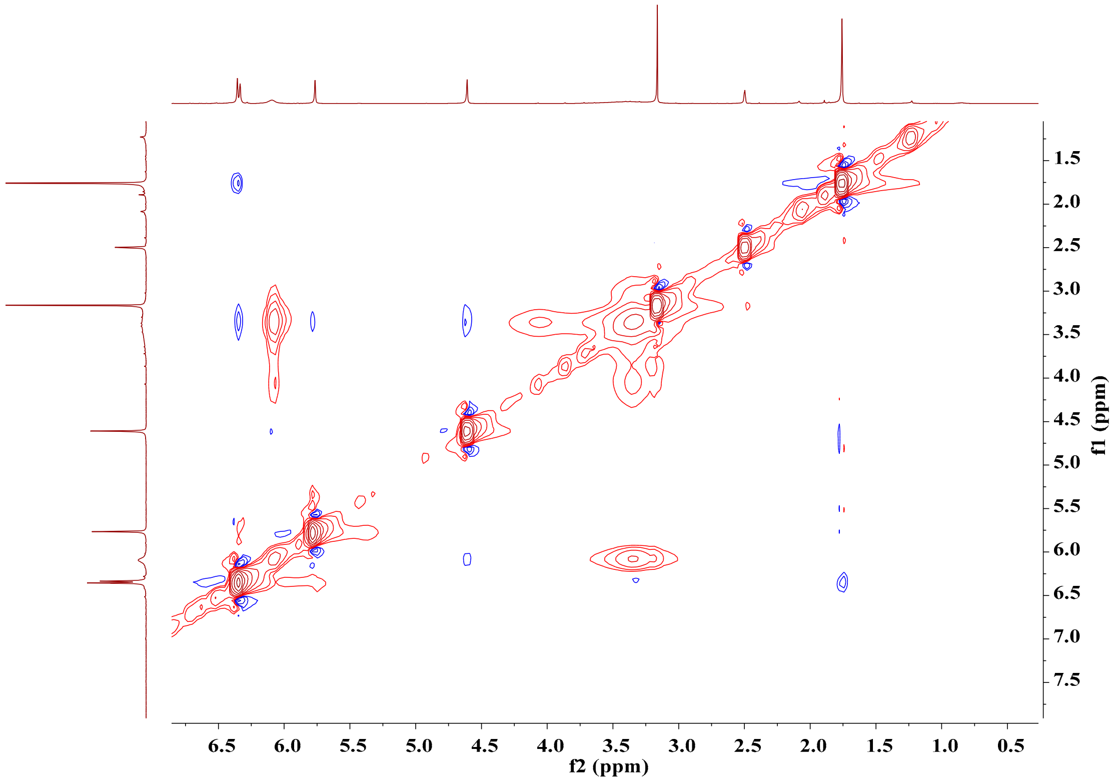

Figure S9. NOESY spectrum of (3*R*,5'*R*)-5-hydroxytalaroflavone (**1**)

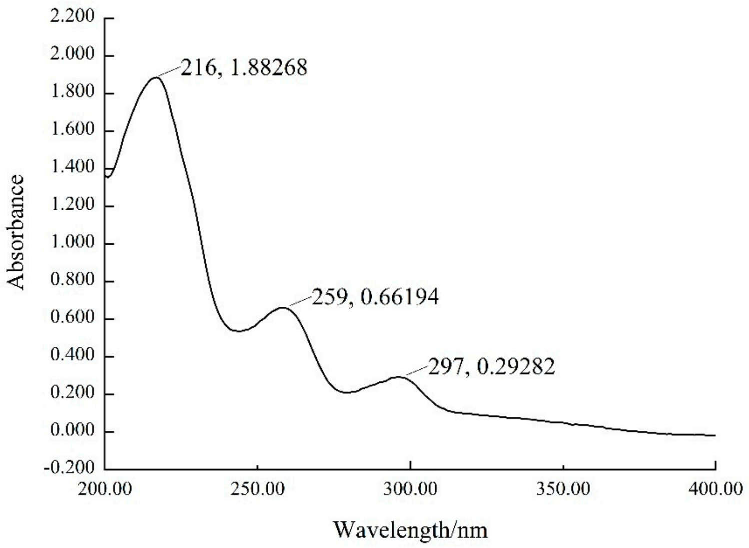

Figure S10. UV spectrum of (3*R*,5'*R*)-5-hydroxytalaroflavone (**1**)

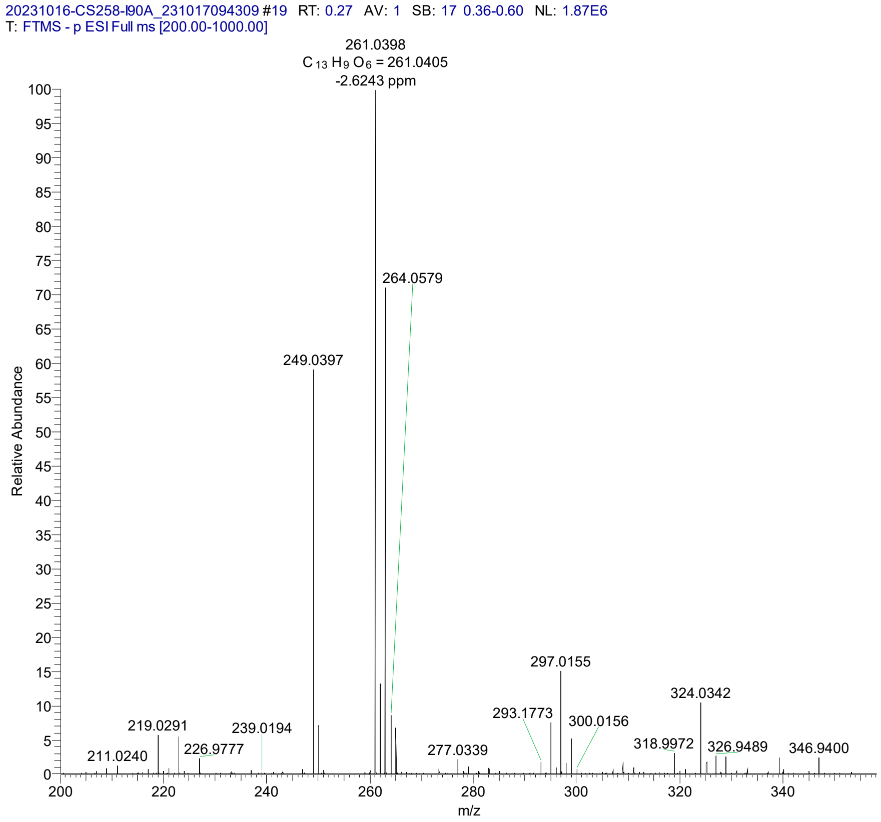

Figure S11. HRESIMS spectrum of talaroisochromenol A (**3**)

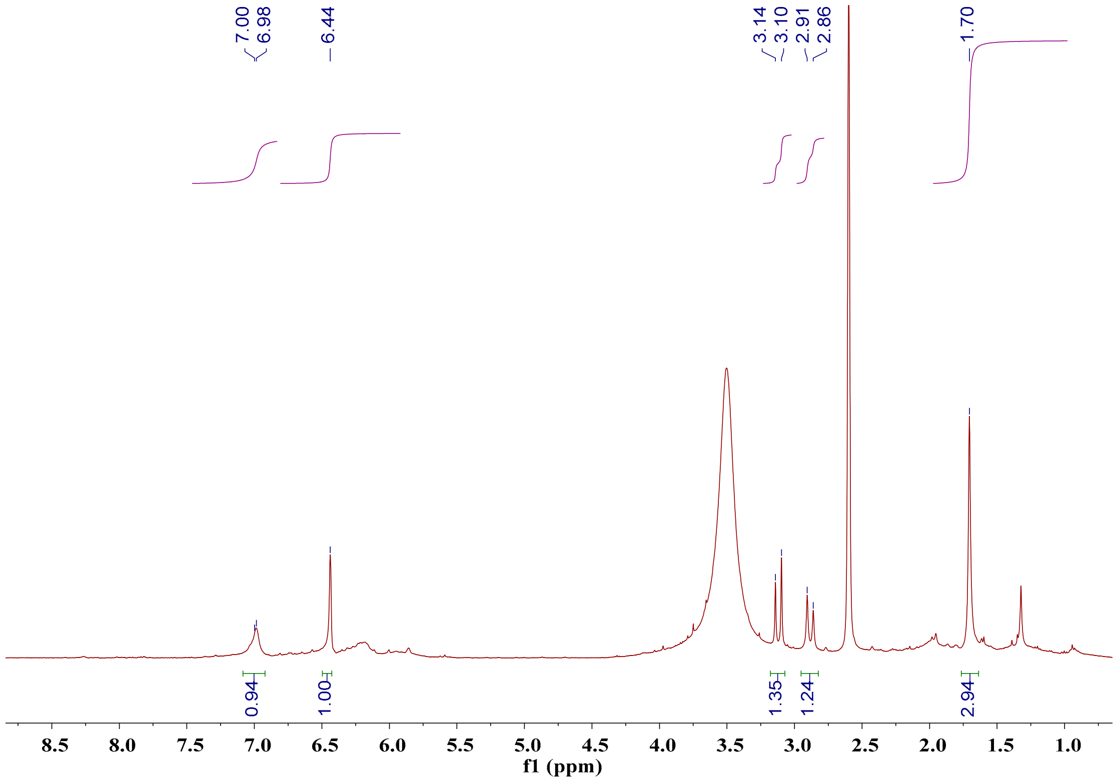

Figure S12. ^{1}H NMR spectrum (400 MHz, DMSO) of talaroisochromenol A (**3**)

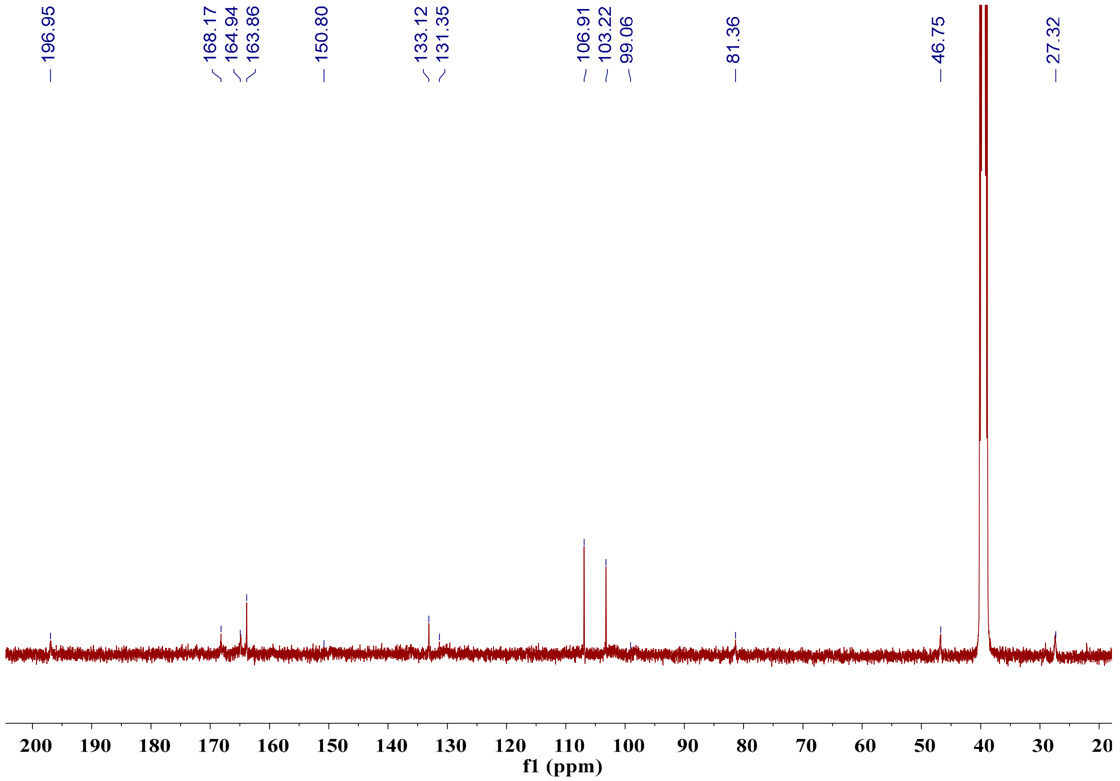

Figure S13. ^{13}C NMR spectrum (100 MHz, DMSO) of talaroisochromenol A (**3**)

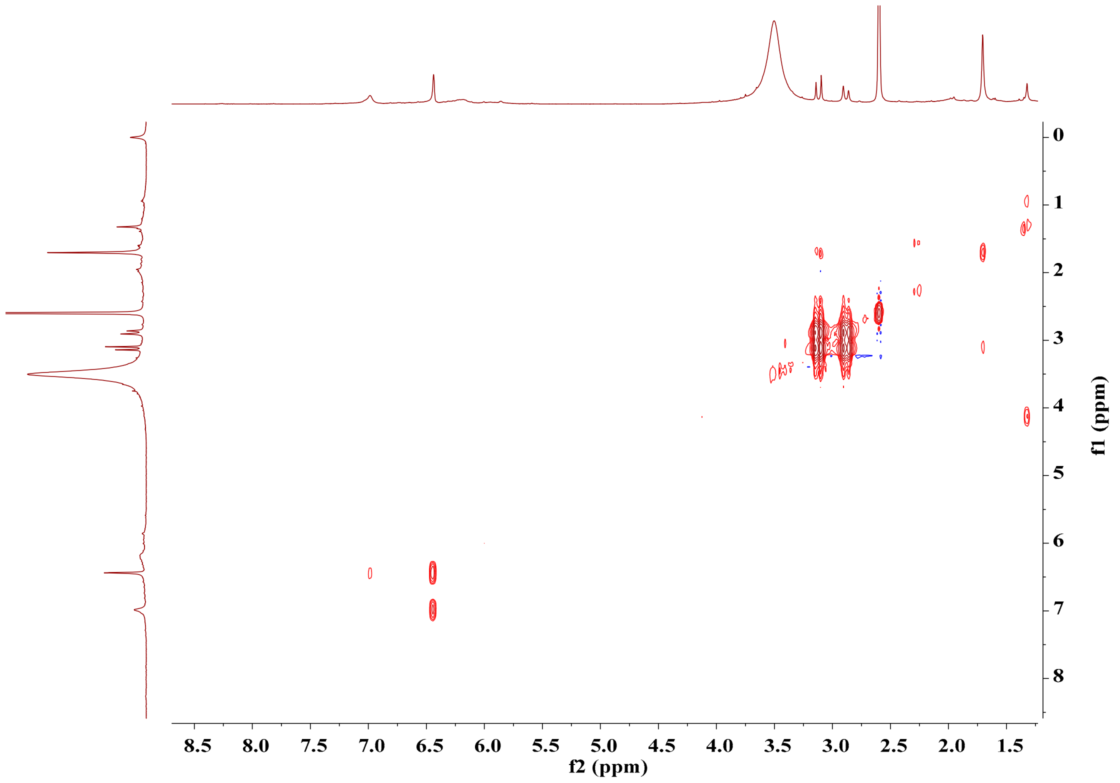

Figure S14. ^{1}H-^{1}H COSY spectrum of talaroisochromenol A (**3**)

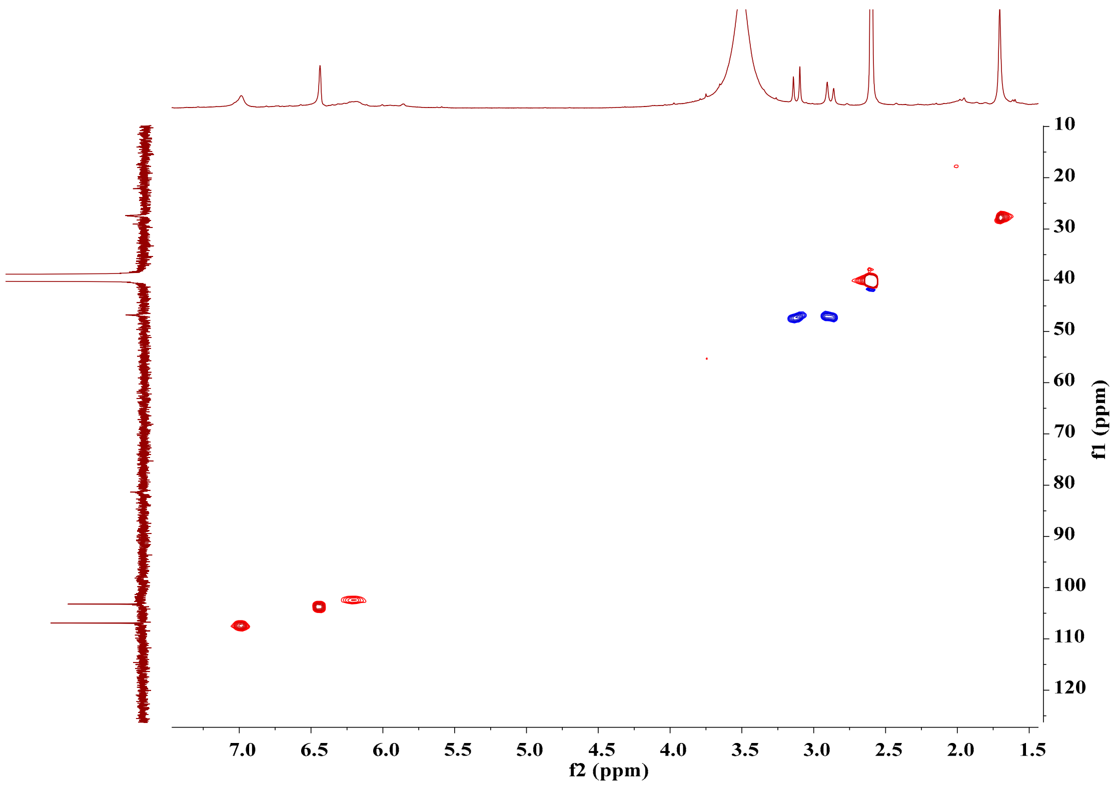

Figure S15. HSQC spectrum of talaroisochromenol A (**3**)

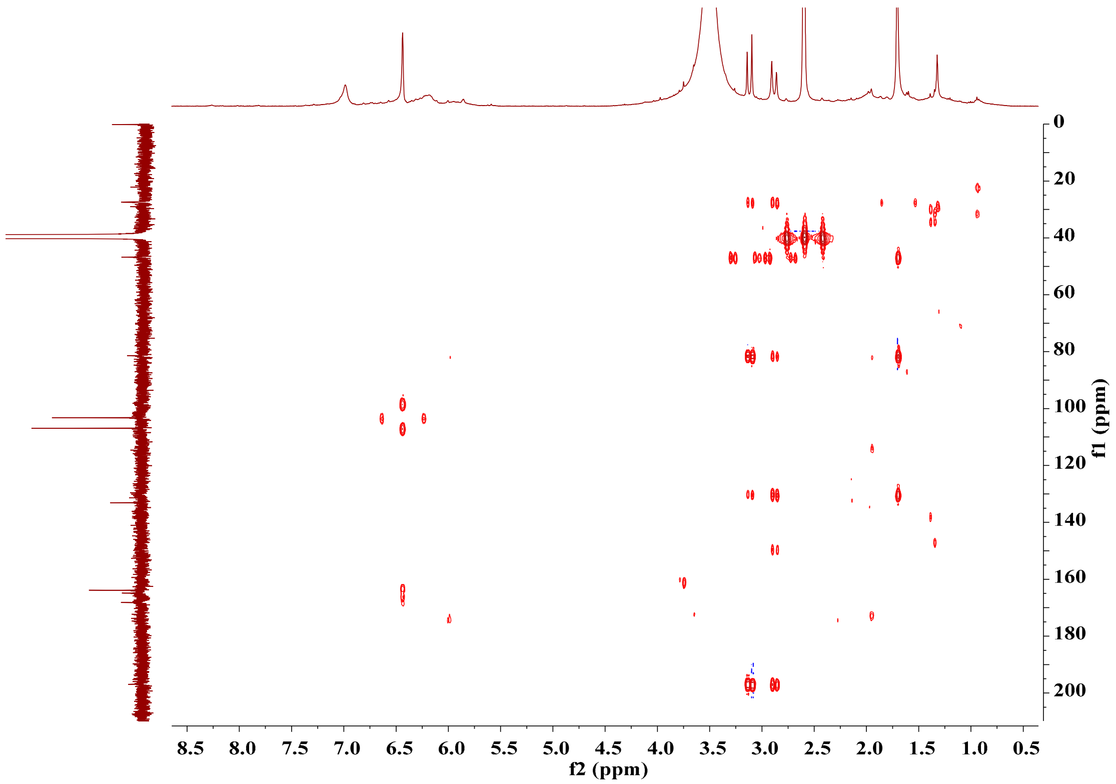

Figure S16. HMBC spectrum of talaroisochromenol A (**3**)

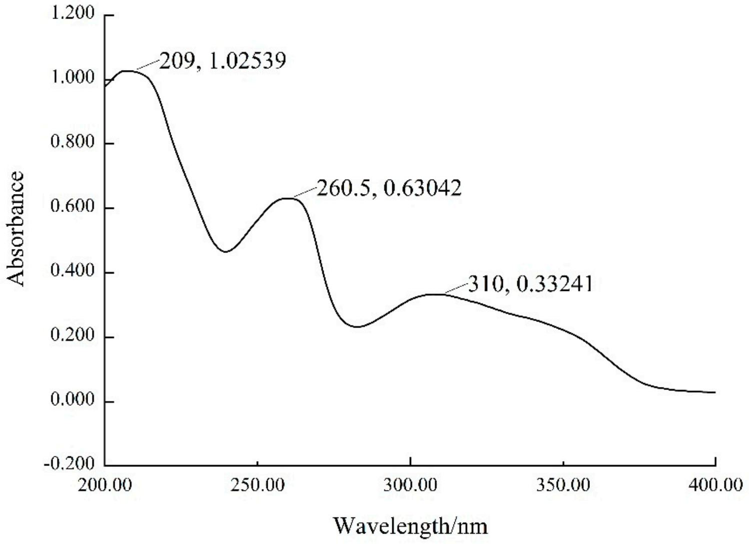

Figure S17. UV spectrum of talaroisochromenol A (**3**)

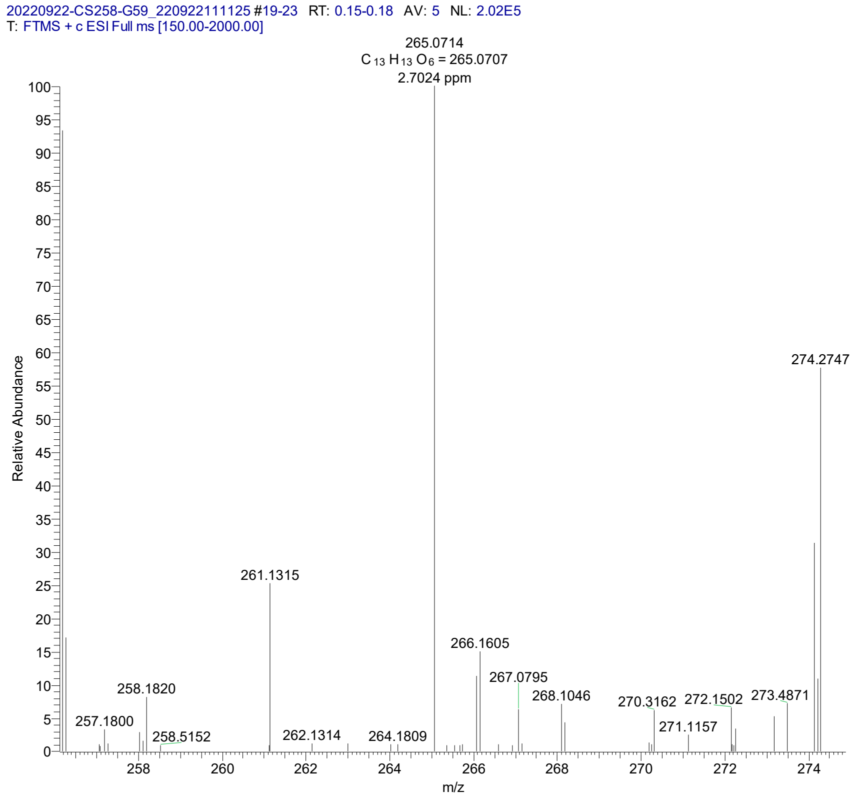

Figure S18. HRESIMS spectrum of talaroisochromenol B (**5**)

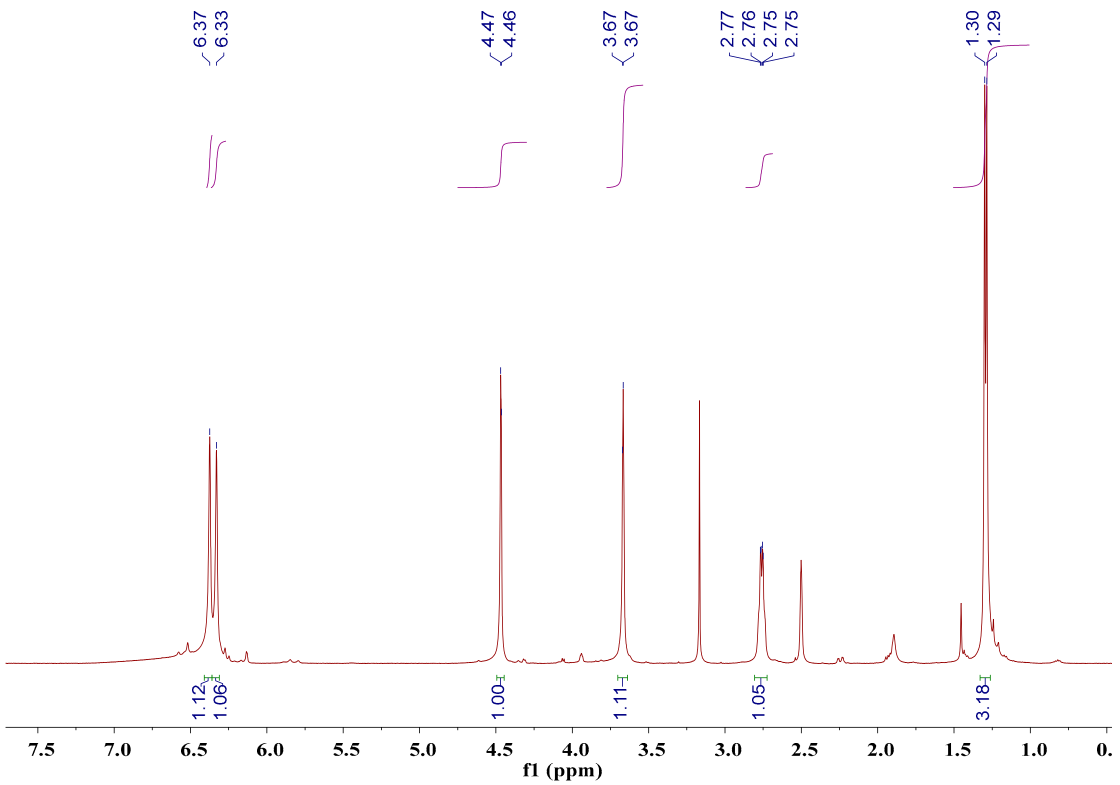

Figure S19. ^{1}H NMR spectrum (500 MHz, DMSO) of talaroisochromenol B (**5**)

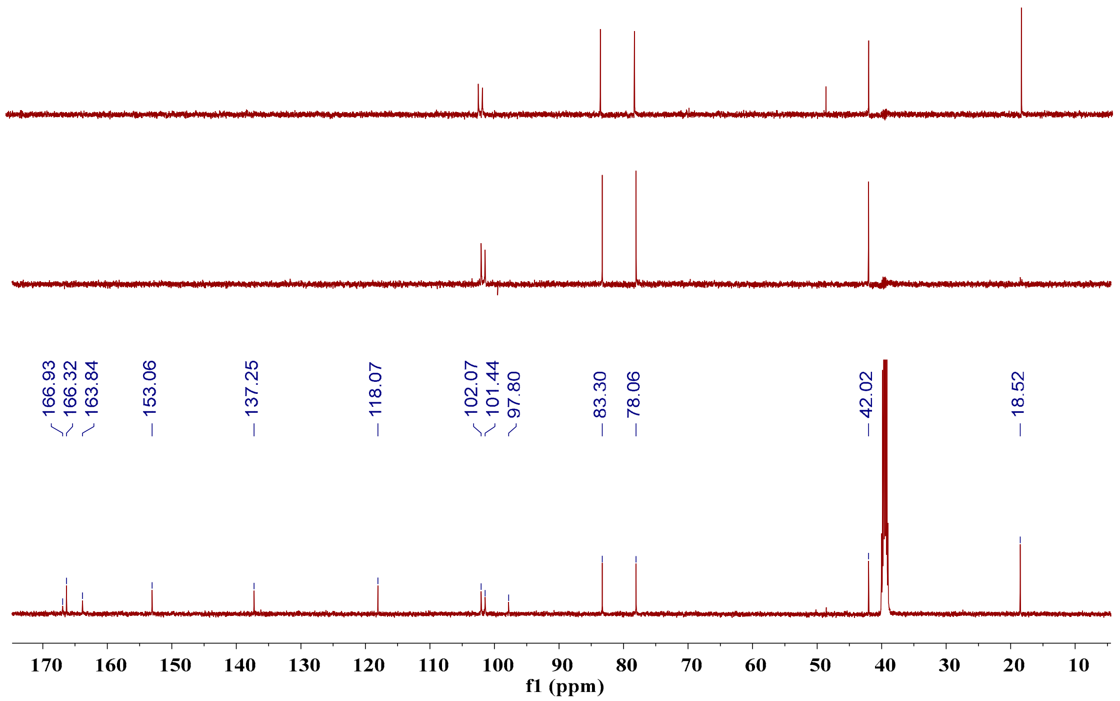

Figure S20. ^{13}C NMR spectrum (125 MHz, DMSO) of talaroisochromenol B (**5**)

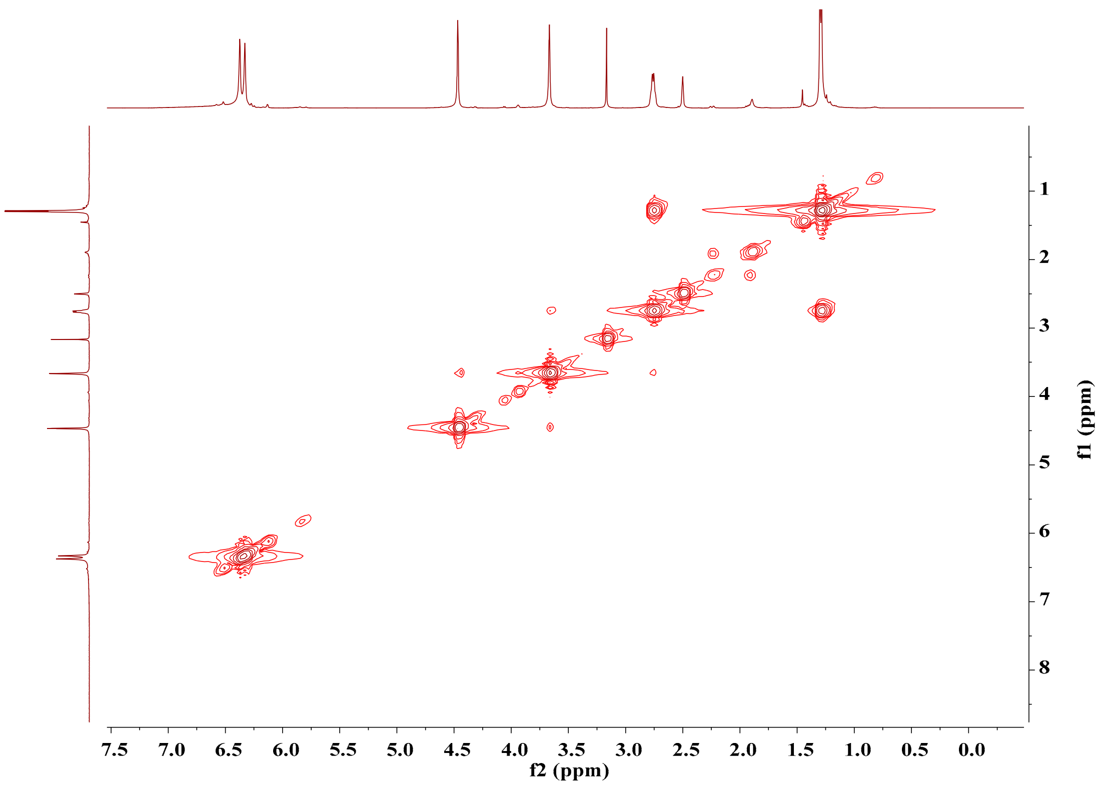

Figure S21. ^{1}H-^{1}H COSY spectrum of talaroisochromenol B (**5**)

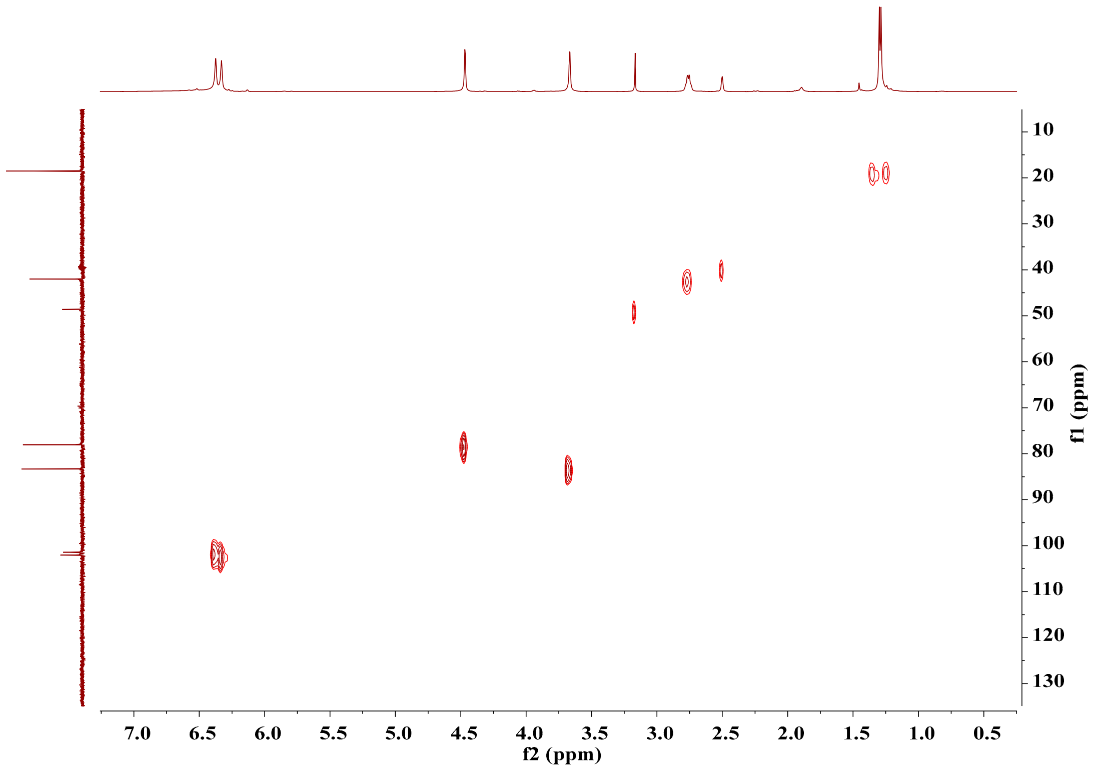

Figure S22. HSQC spectrum of talaroisochromenol B (**5**)

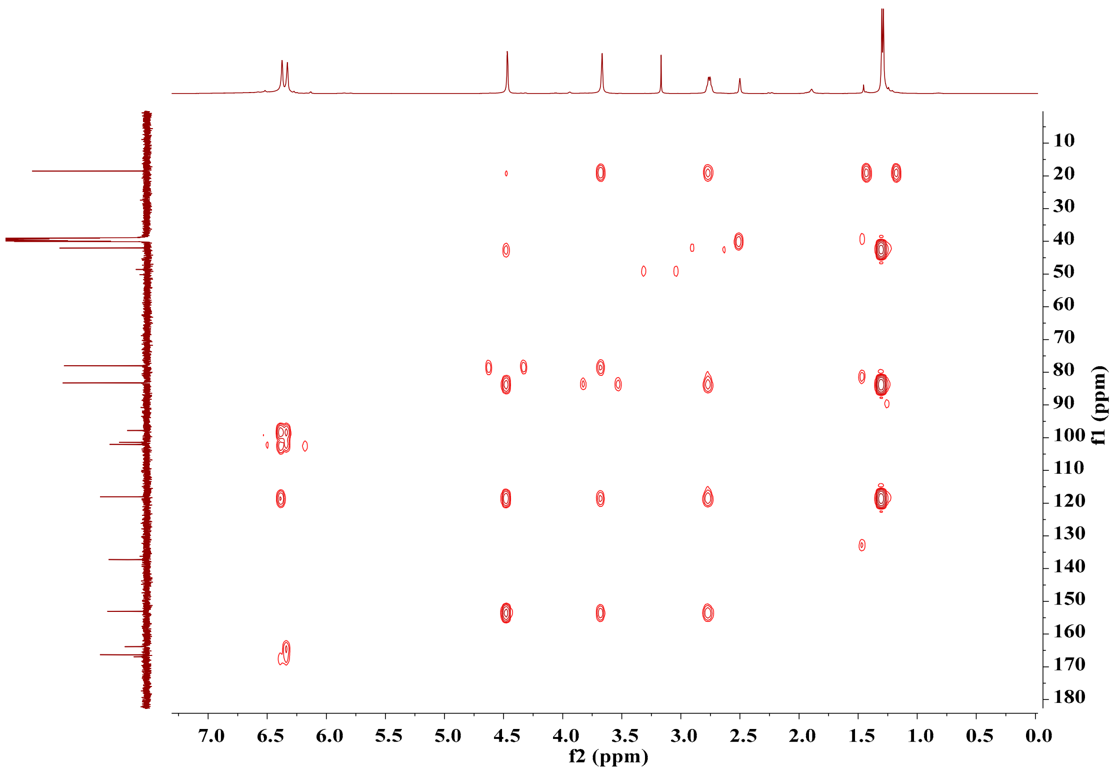

Figure S23. HMBC spectrum of talaroisochromenol B (**5**)

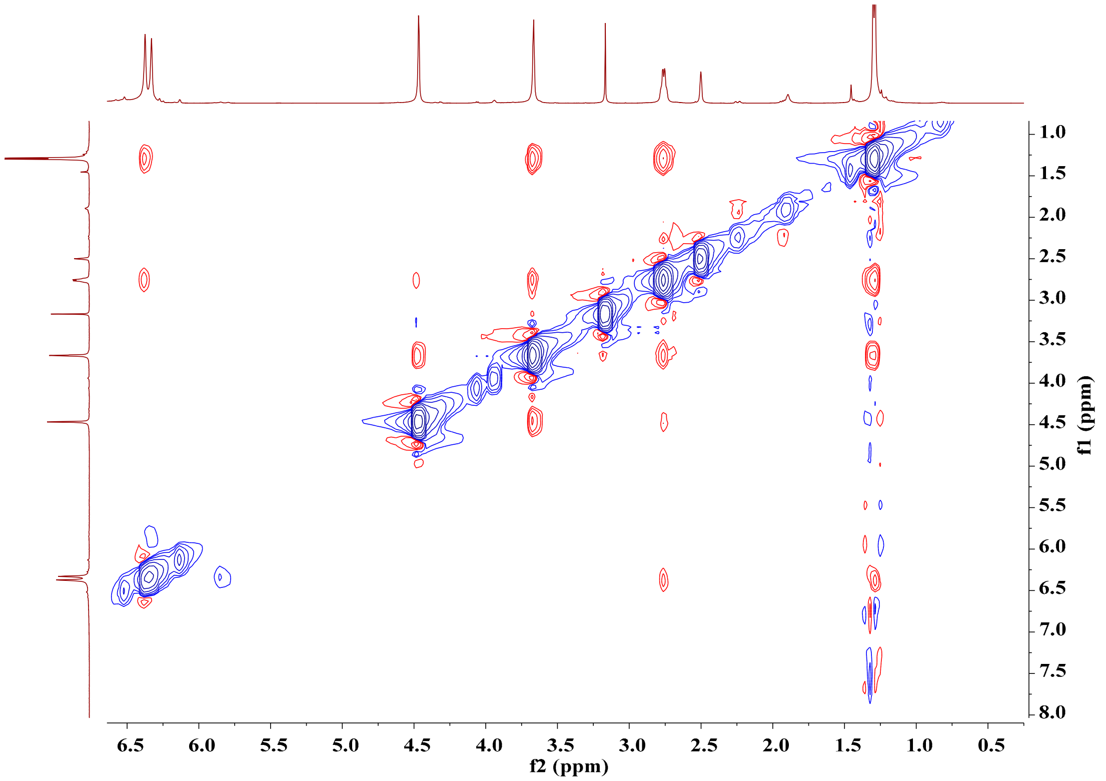

Figure S24. NOESY spectrum of talaroisochromenol B (**5**)

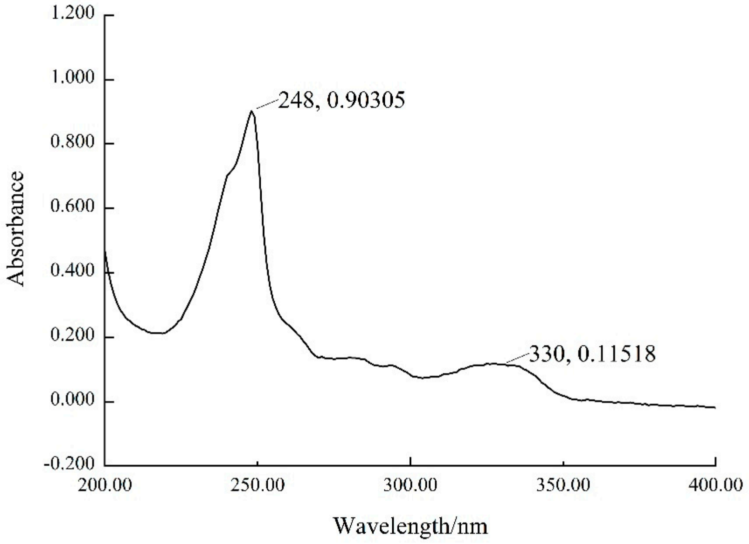

Figure S25. UV spectrum of talaroisochromenol B (**5**)

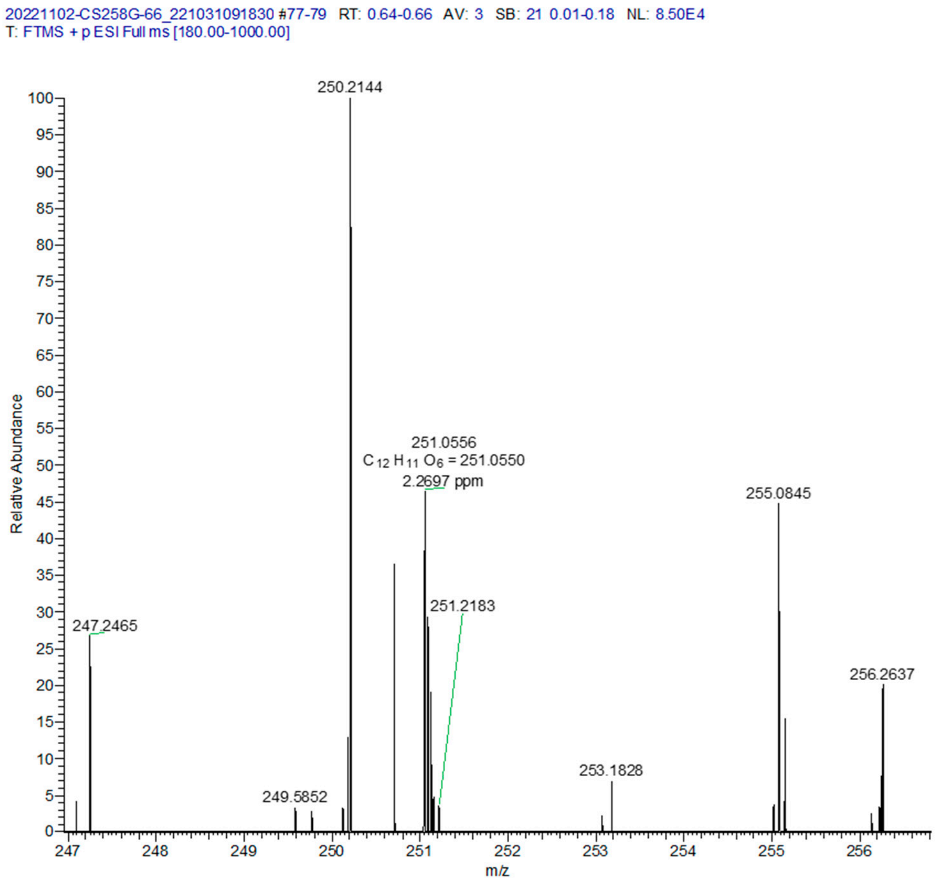

Figure S26. HRESIMS spectrum of talaroisochromenol C (**11**)

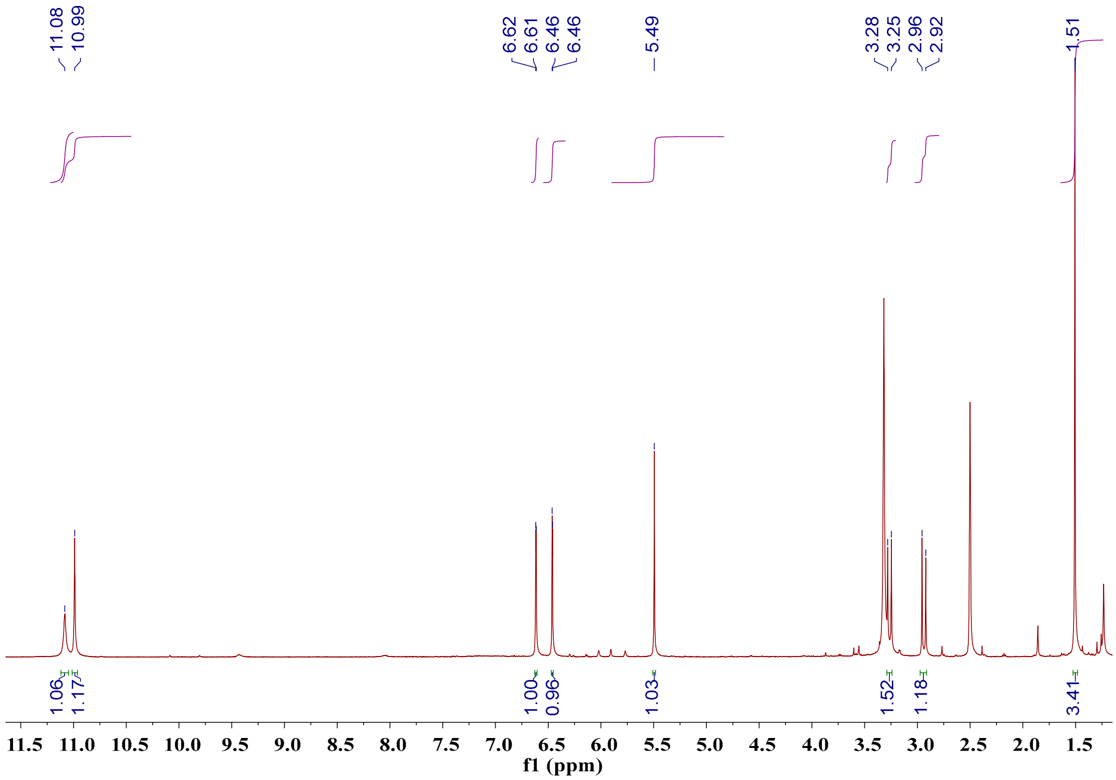

Figure S27. ^{1}H NMR spectrum (500 MHz, DMSO) of talaroisochromenol C (**11**)

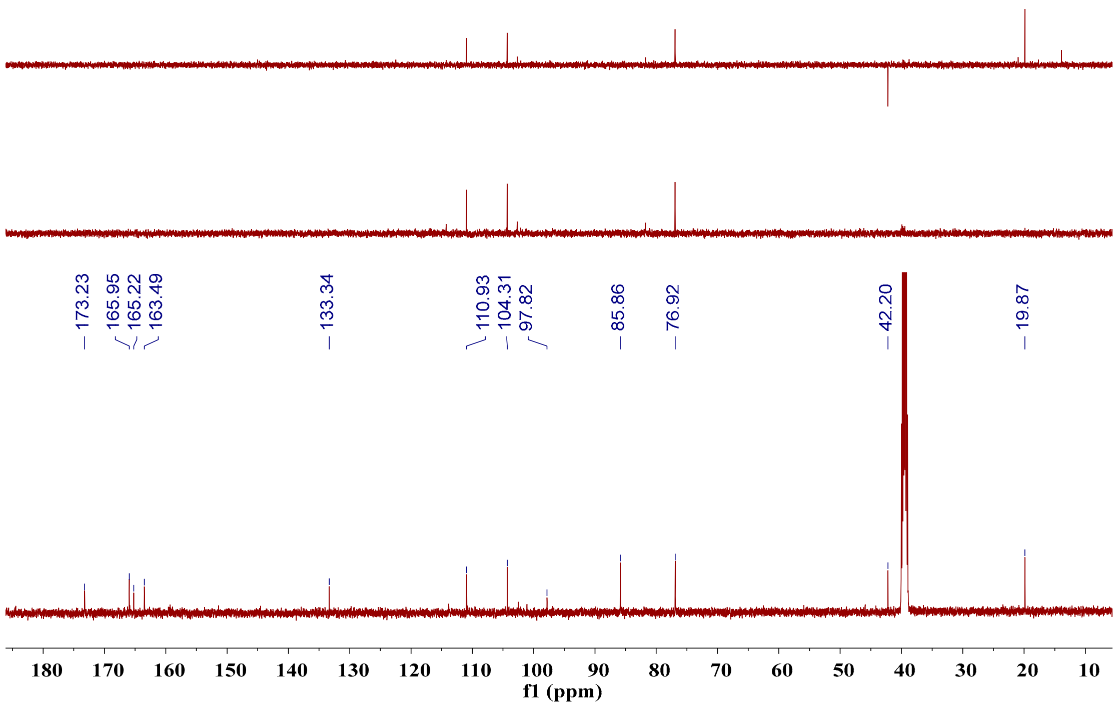

Figure S28. ^{13}C NMR spectrum (125 MHz, DMSO) of talaroisochromenol C (**11**)

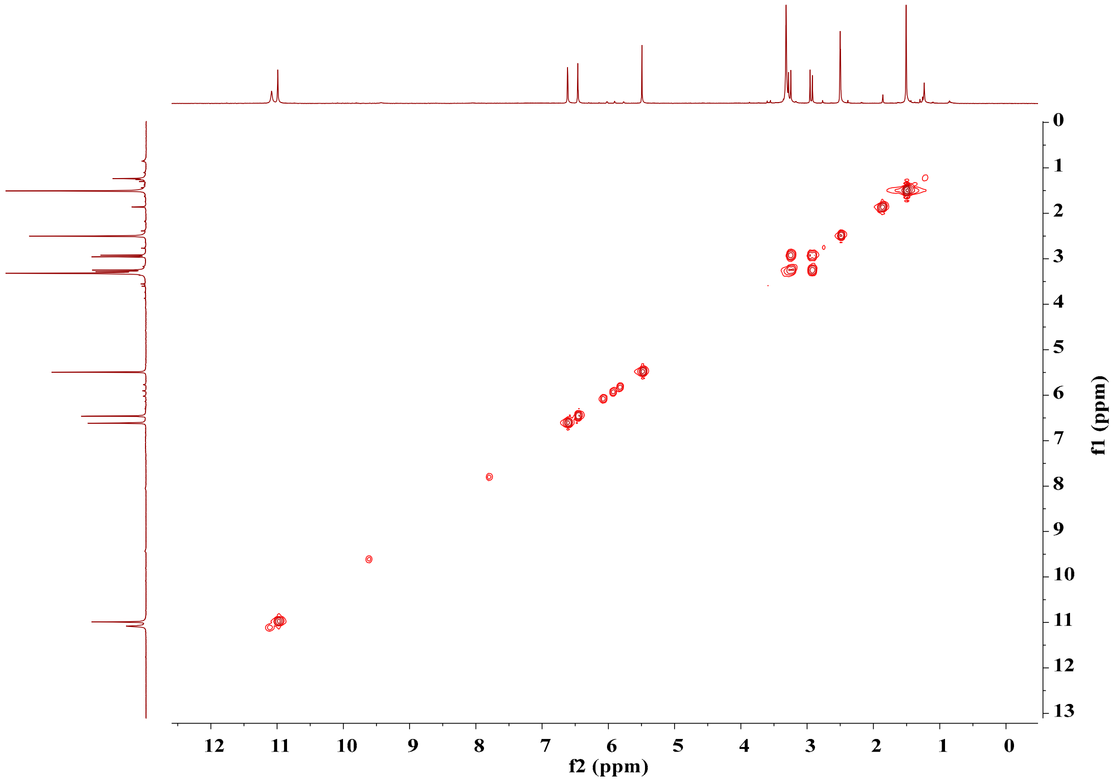

Figure S29. ^{1}H-^{1}H COSY spectrum of talaroisochromenol C (**11**)

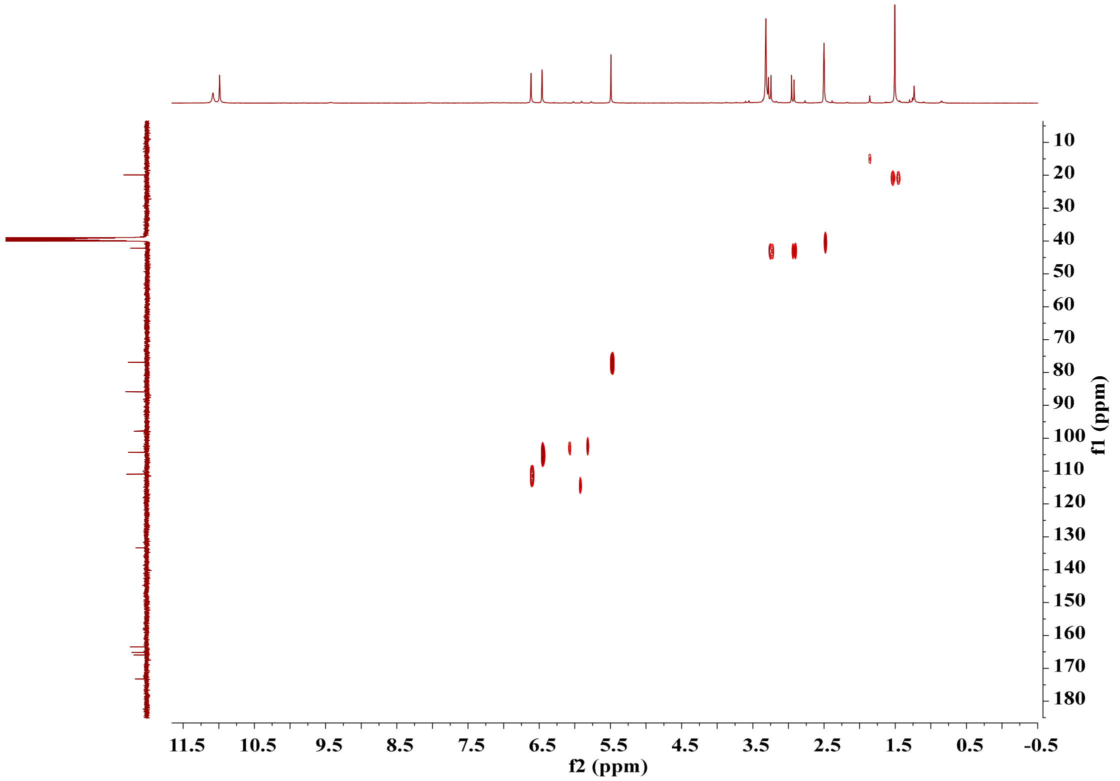

Figure S30. HSQC spectrum of talaroisochromenol C (**11**)

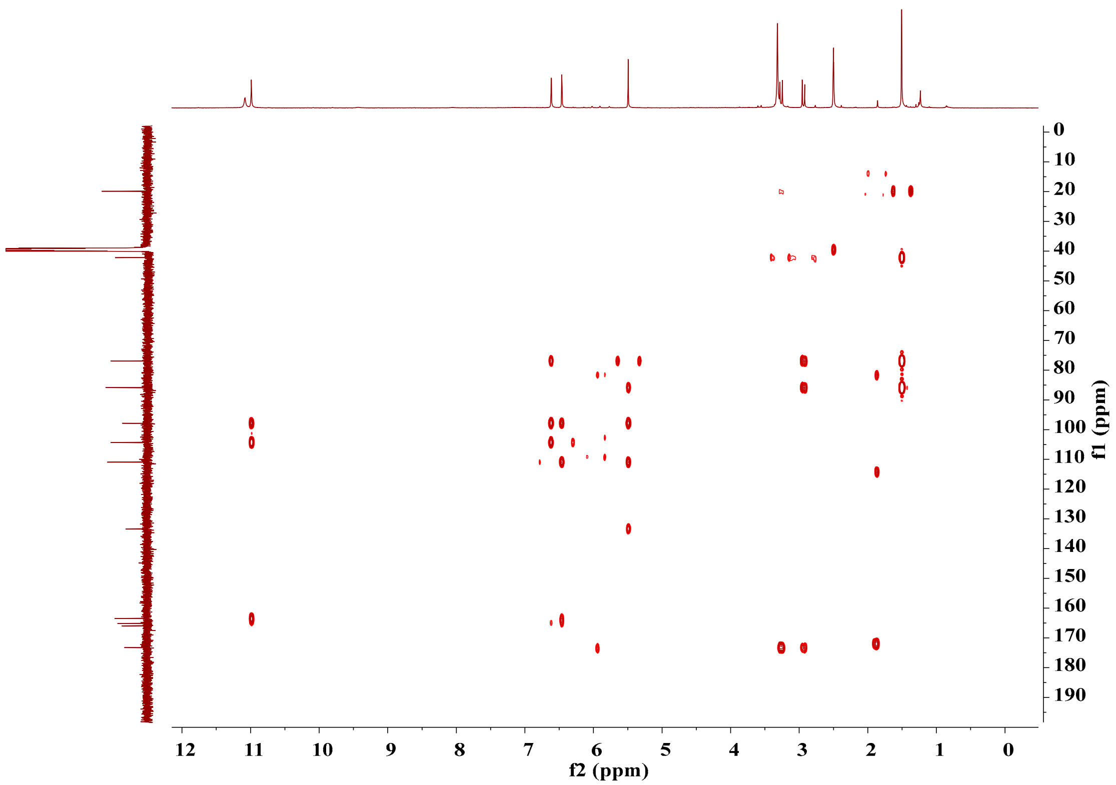

Figure S31. HMBC spectrum of talaroisochromenol C (**11**)

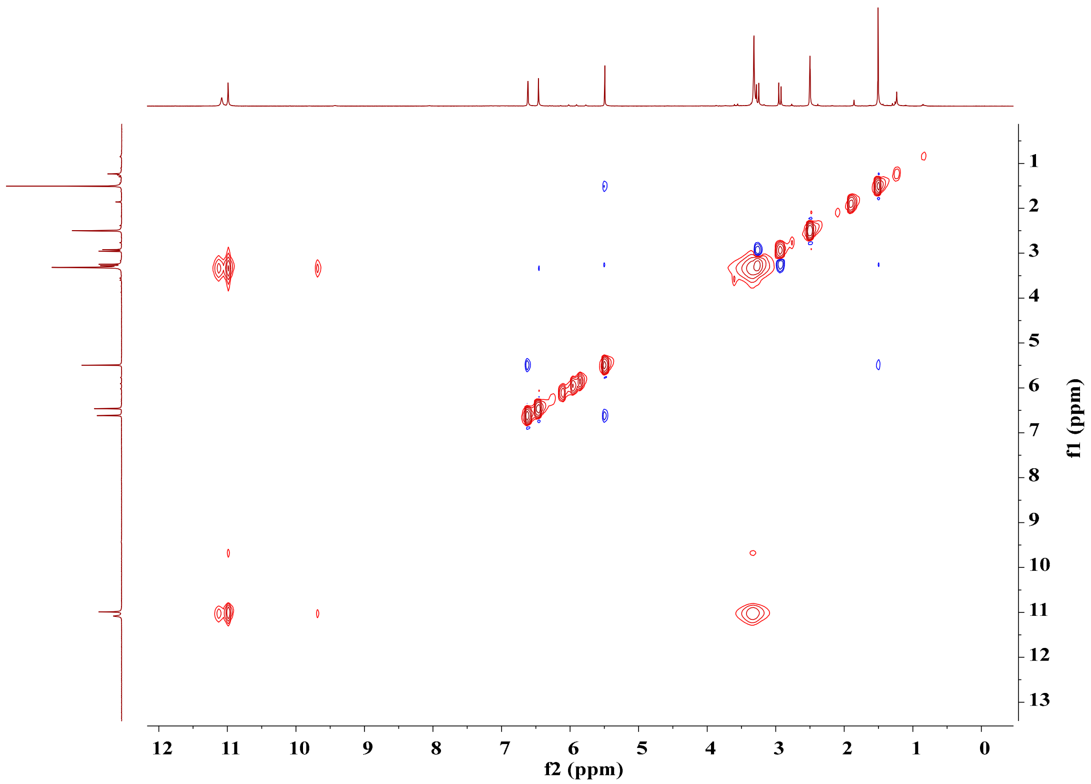

Figure S32. NOESY spectrum of talaroisochromenol C (**11**)

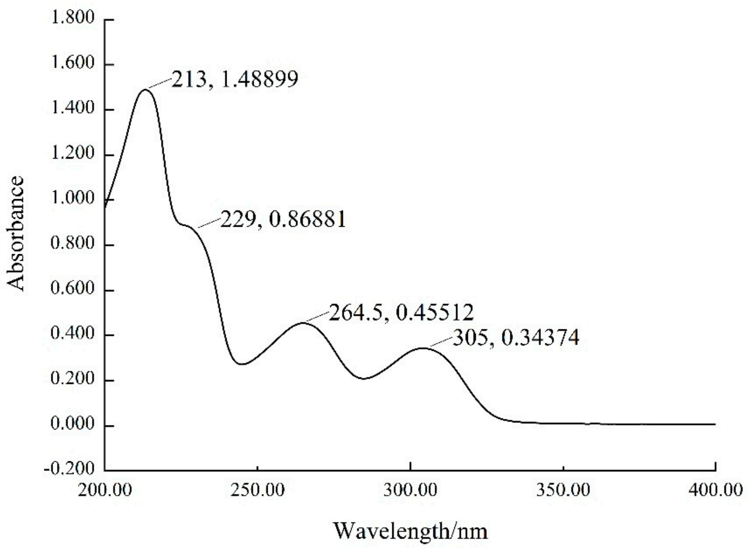

Figure S33. UV spectrum of talaroisochromenol C (**11**)

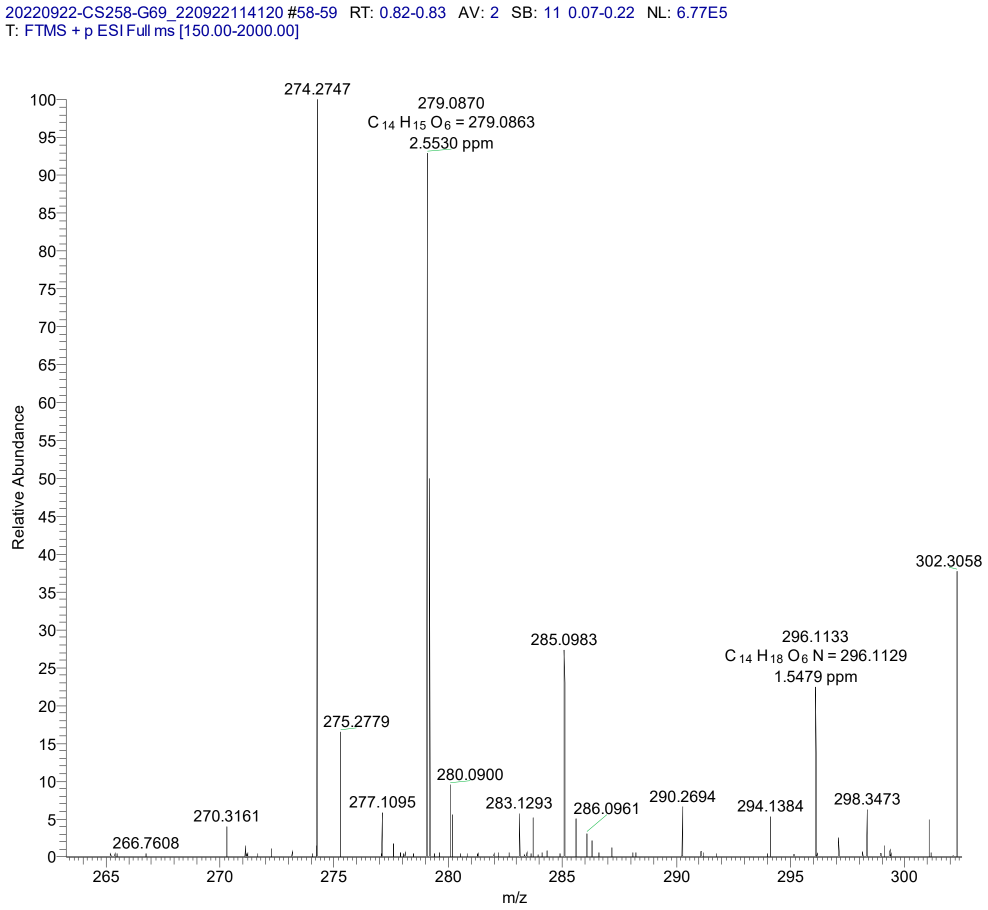

Figure S34. HRESIMS spectrum of (8*R*,9*R*,10a*R*)-5-hydroxyaltenuene (**13**)

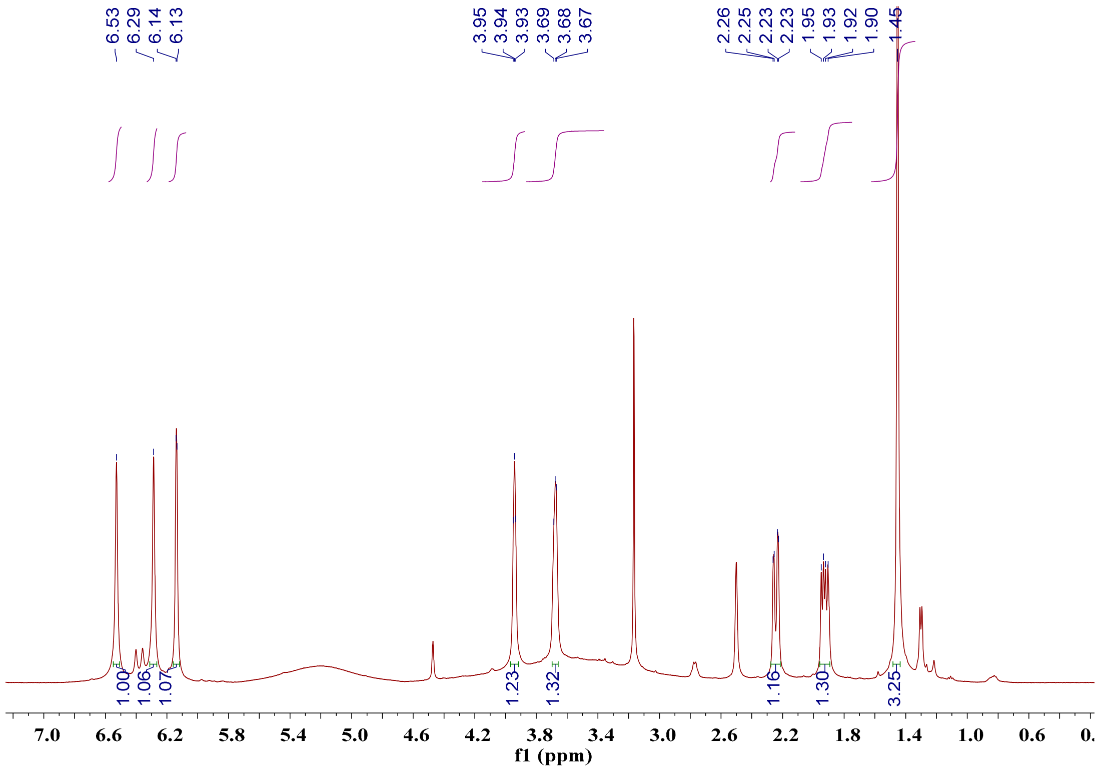

Figure S35. ^{1}H NMR spectrum (500 MHz, DMSO) of (8*R*,9*R*,10a*R*)-5-hydroxyaltenuene (**13**)

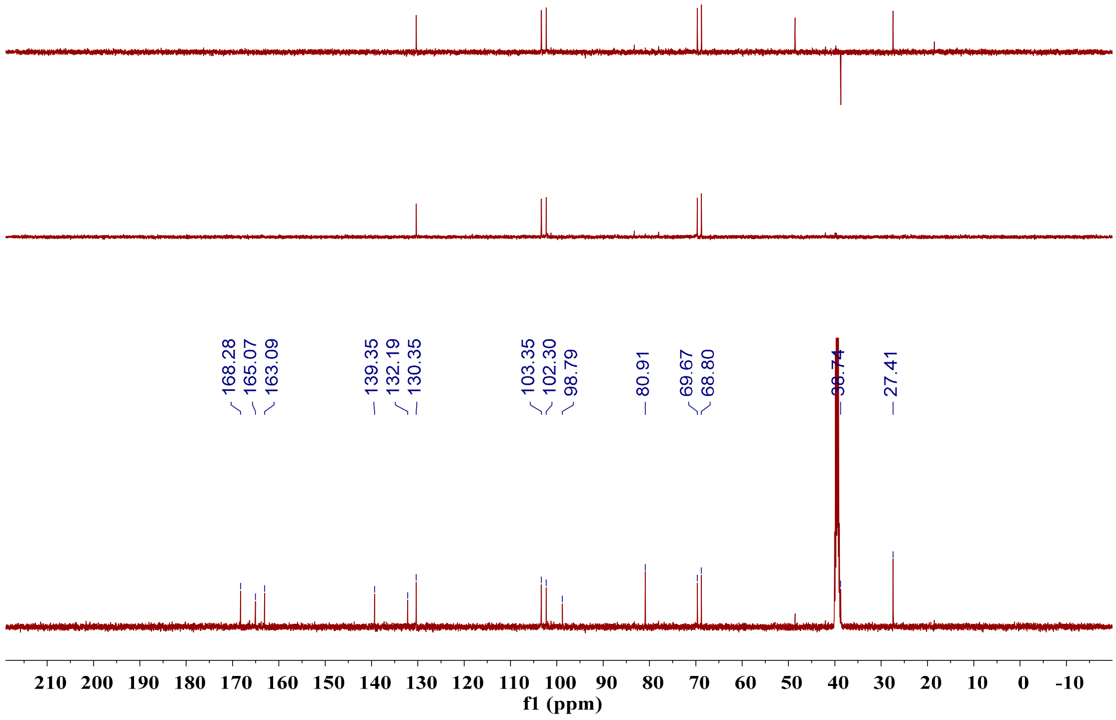

Figure S36. ^{13}C NMR spectrum (125 MHz, DMSO) of (8*R*,9*R*,10a*R*)-5-hydroxyaltenuene (**13**)

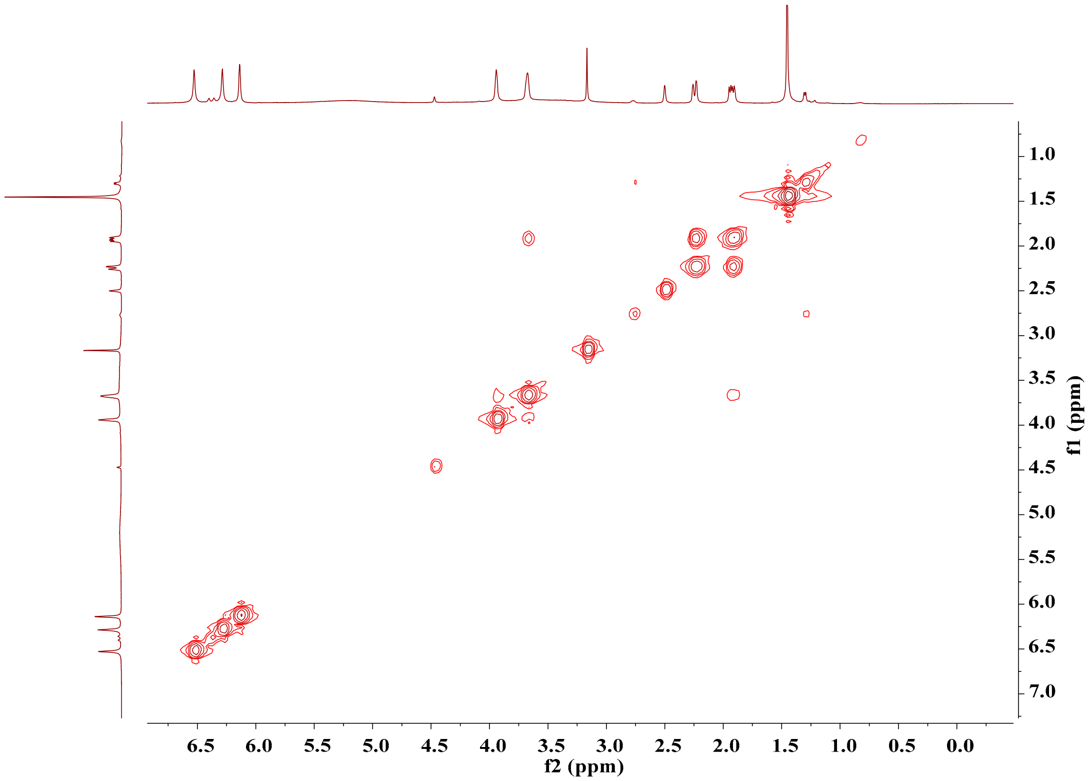

Figure S37. ^{1}H-^{1}H COSY spectrum of (8*R*,9*R*,10a*R*)-5-hydroxyaltenuene (**13**)

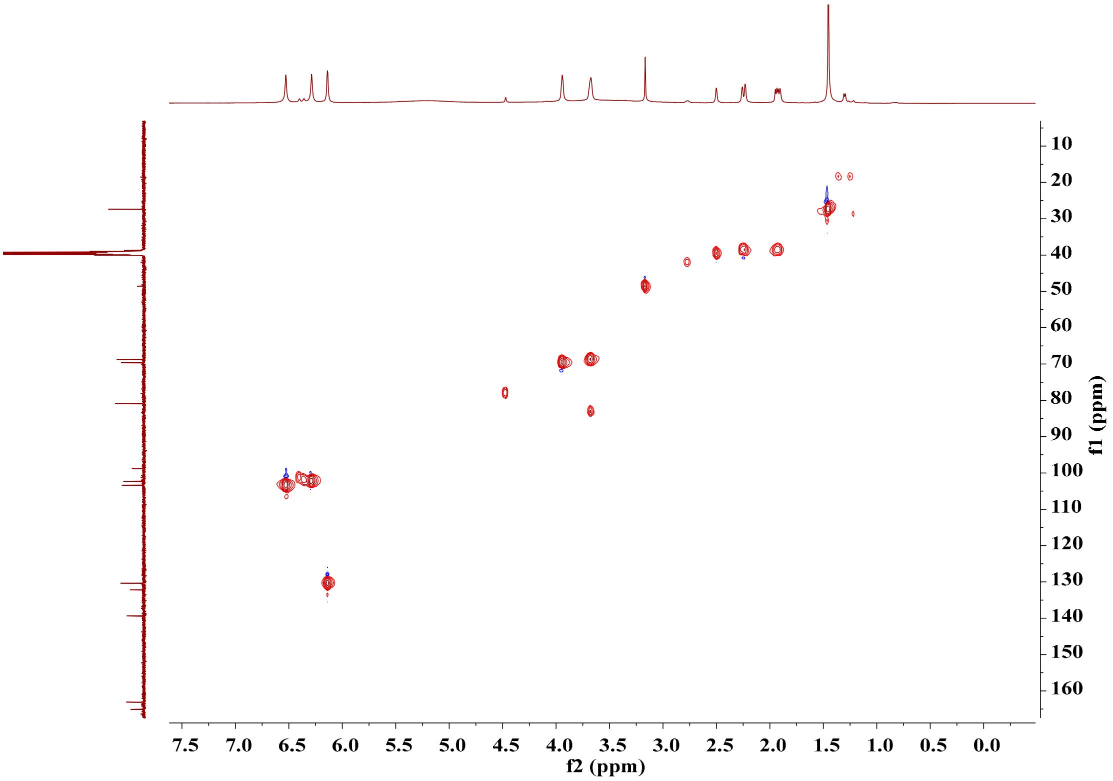

Figure S38. HSQC spectrum of (8*R*,9*R*,10a*R*)-5-hydroxyaltenuene (**13**)

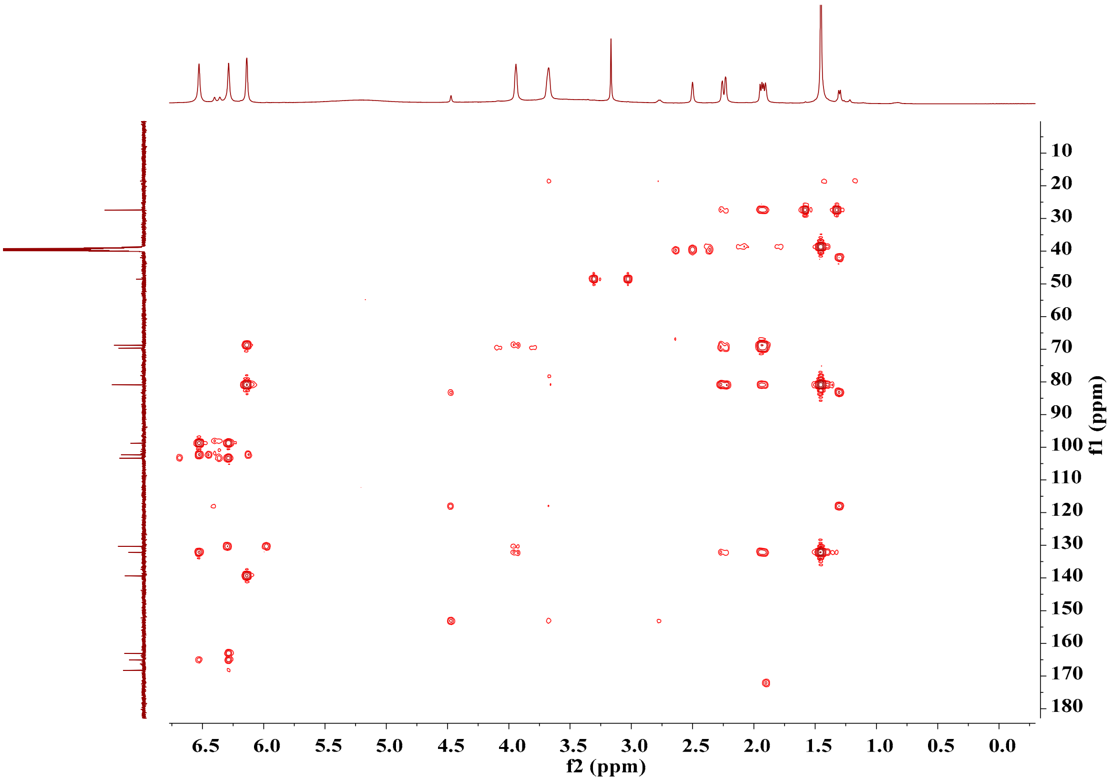

Figure S39. HMBC spectrum of (8*R*,9*R*,10a*R*)-5-hydroxyaltenuene (**13**)

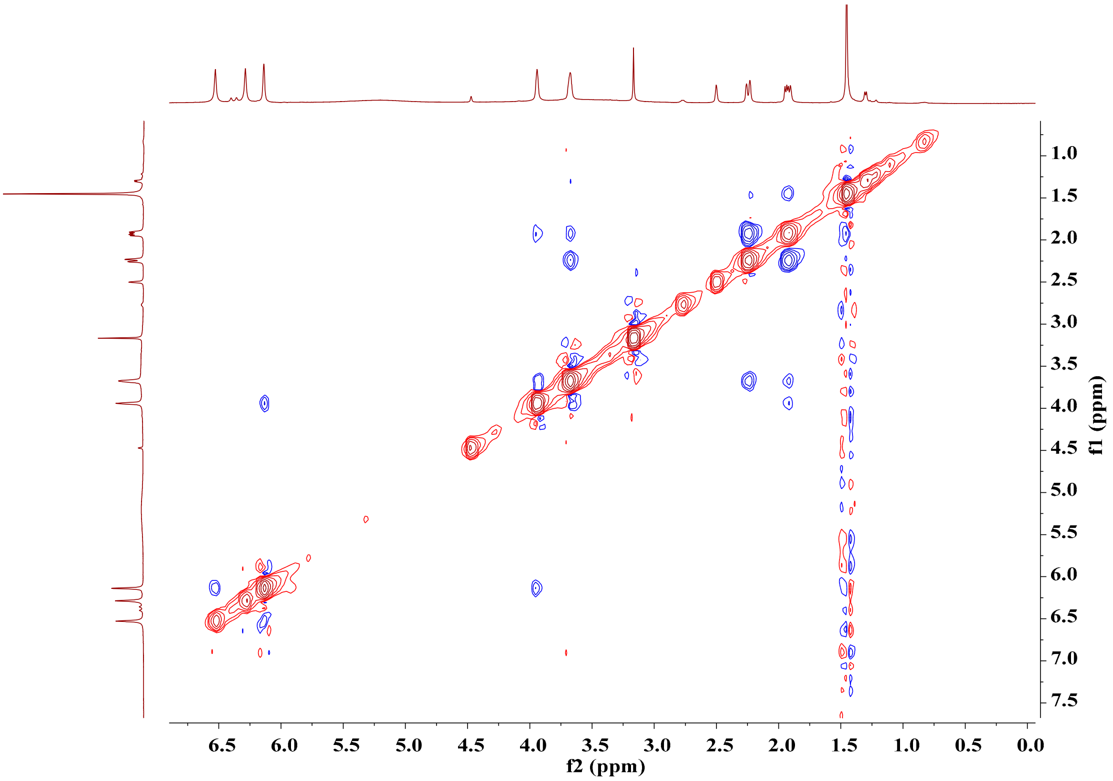

Figure S40. NOESY spectrum of (8*R*,9*R*,10a*R*)-5-hydroxyaltenuene (**13**)

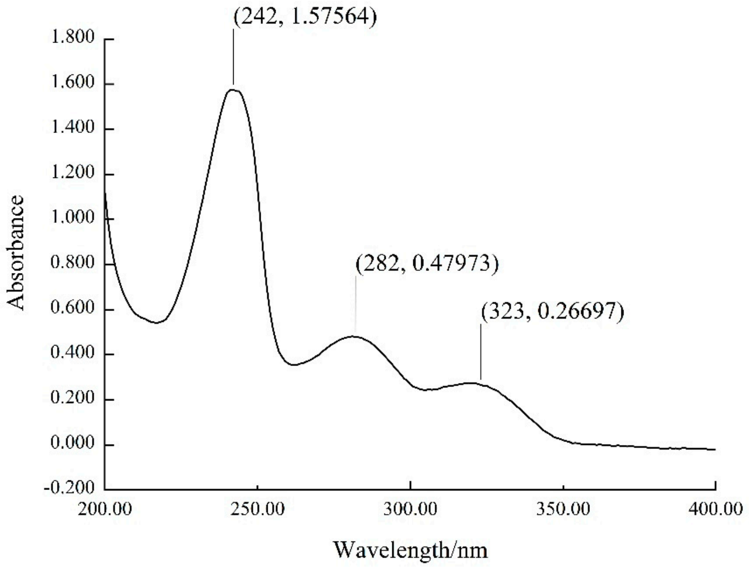

Figure S41. UV spectrum of (8*R*,9*R*,10a*R*)-5-hydroxyaltenuene (**13**)

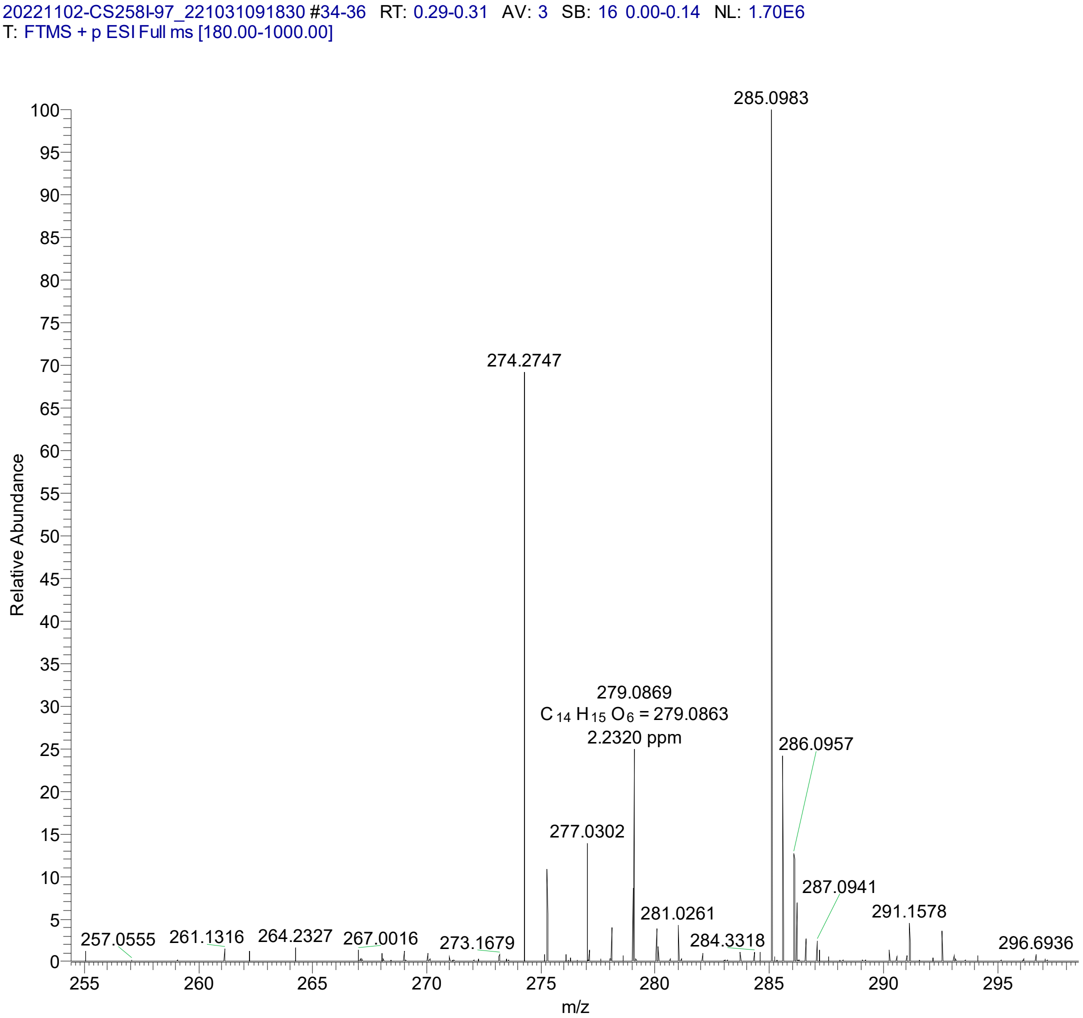

Figure S42. HRESIMS spectrum of (8*R*,9*R*,10a*S*)-5-hydroxyaltenuene (**14**)

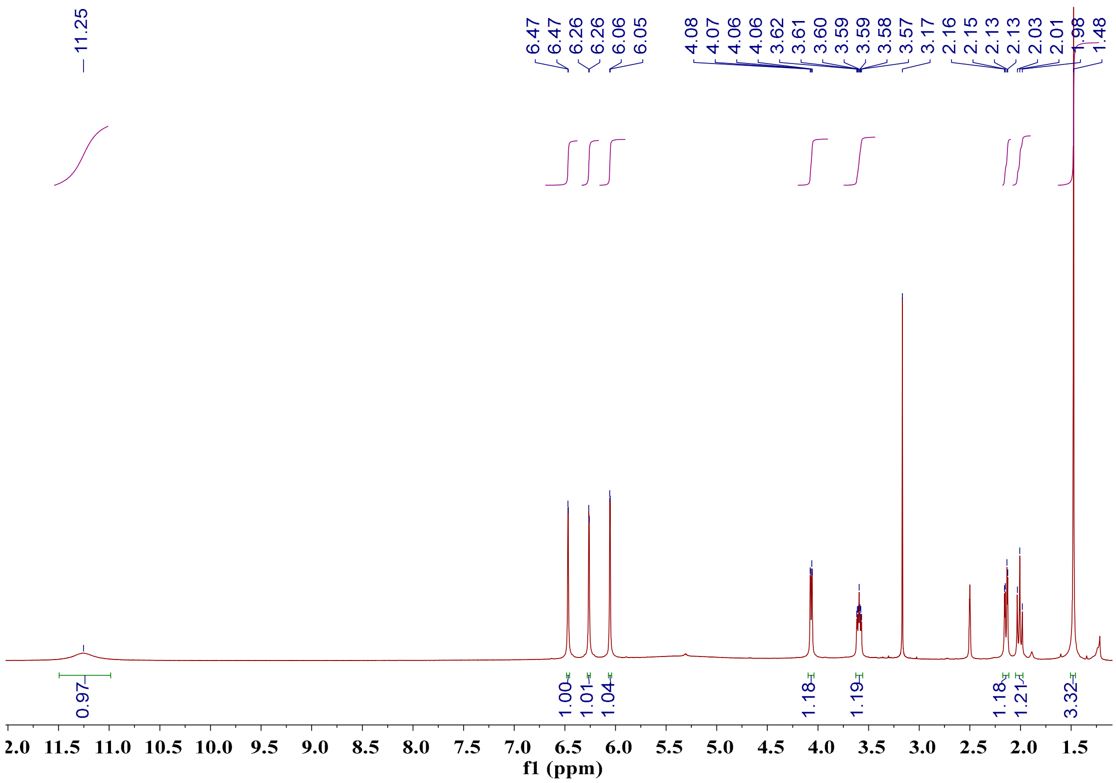

Figure S43. ^{1}H NMR spectrum (500 MHz, DMSO) of (8*R*,9*R*,10a*S*)-5-hydroxyaltenuene (**14**)

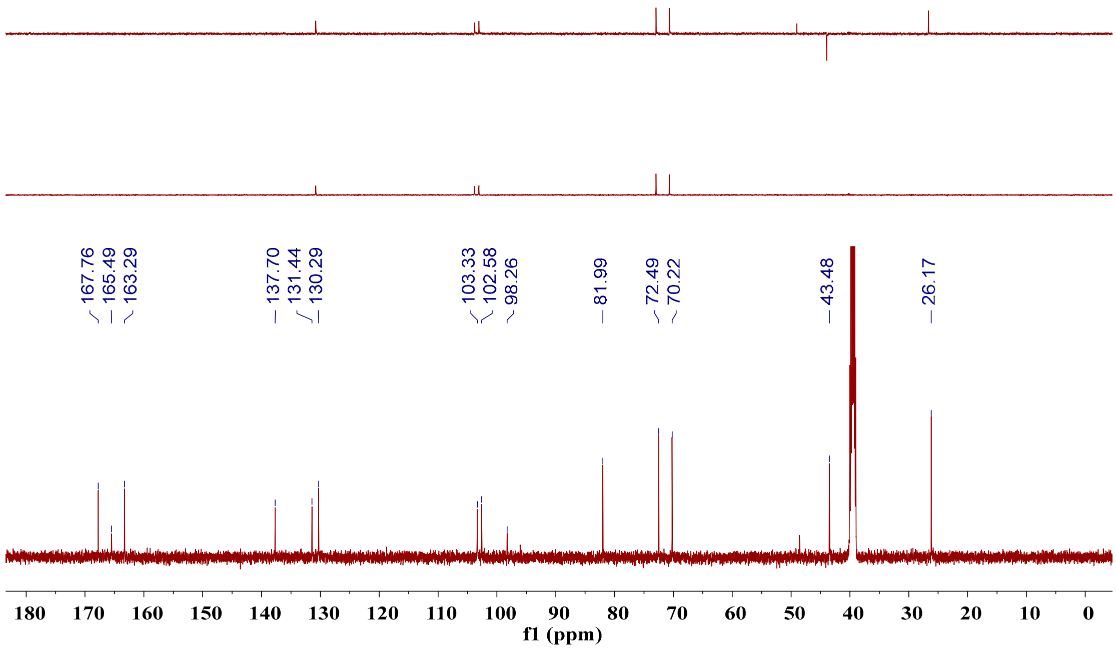

Figure S44. ^{13}C NMR spectrum (125 MHz, DMSO) of (8*R*,9*R*,10a*S*)-5-hydroxyaltenuene (**14**)

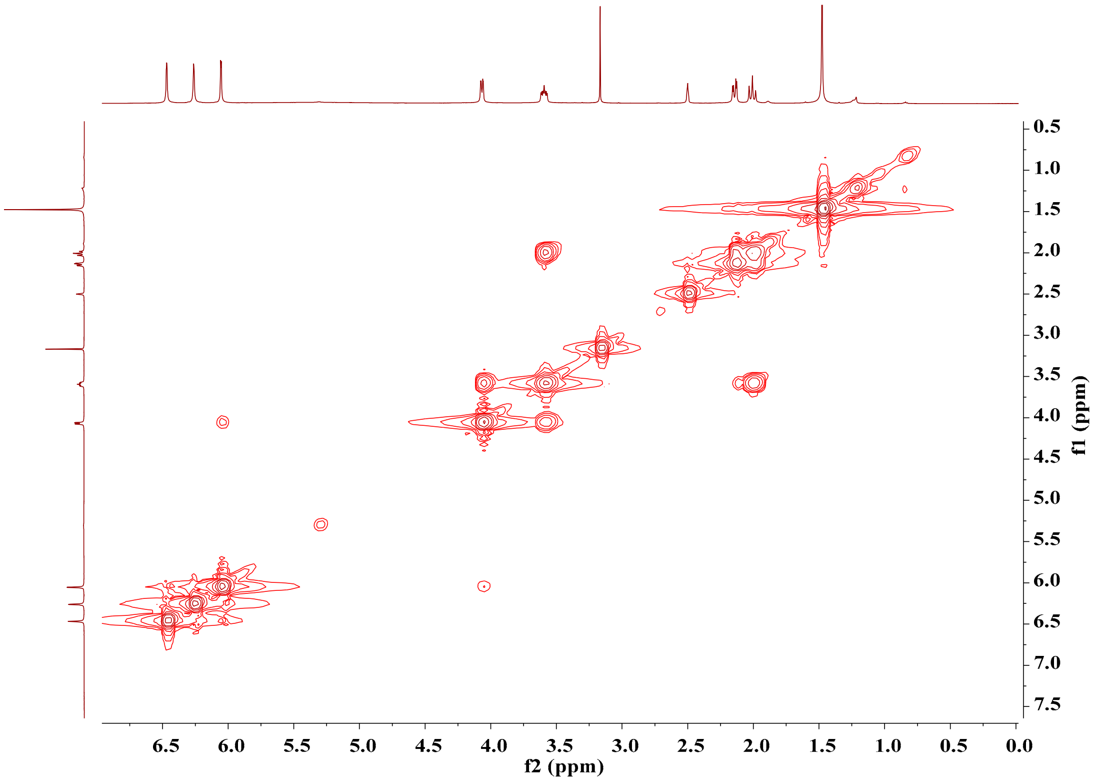

Figure S45. ^{1}H-^{1}H COSY spectrum of (8*R*,9*R*,10a*S*)-5-hydroxyaltenuene (**14**)

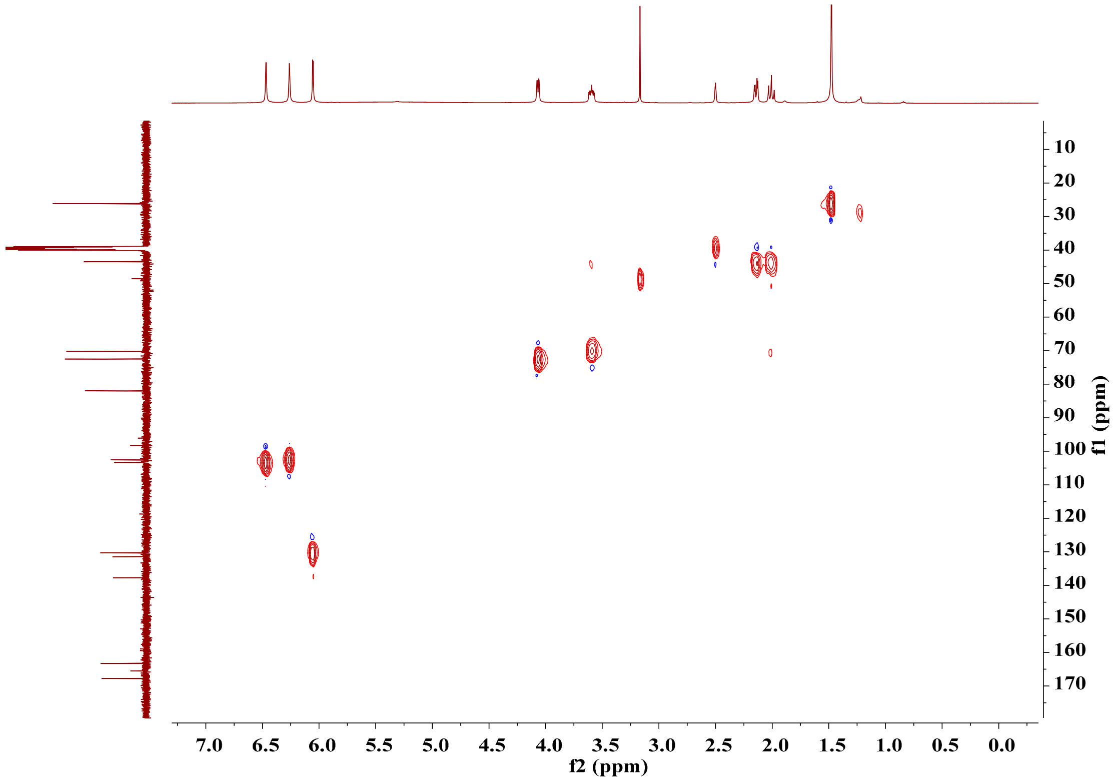

Figure S46. HSQC spectrum of (8*R*,9*R*,10a*S*)-5-hydroxyaltenuene (**14**)

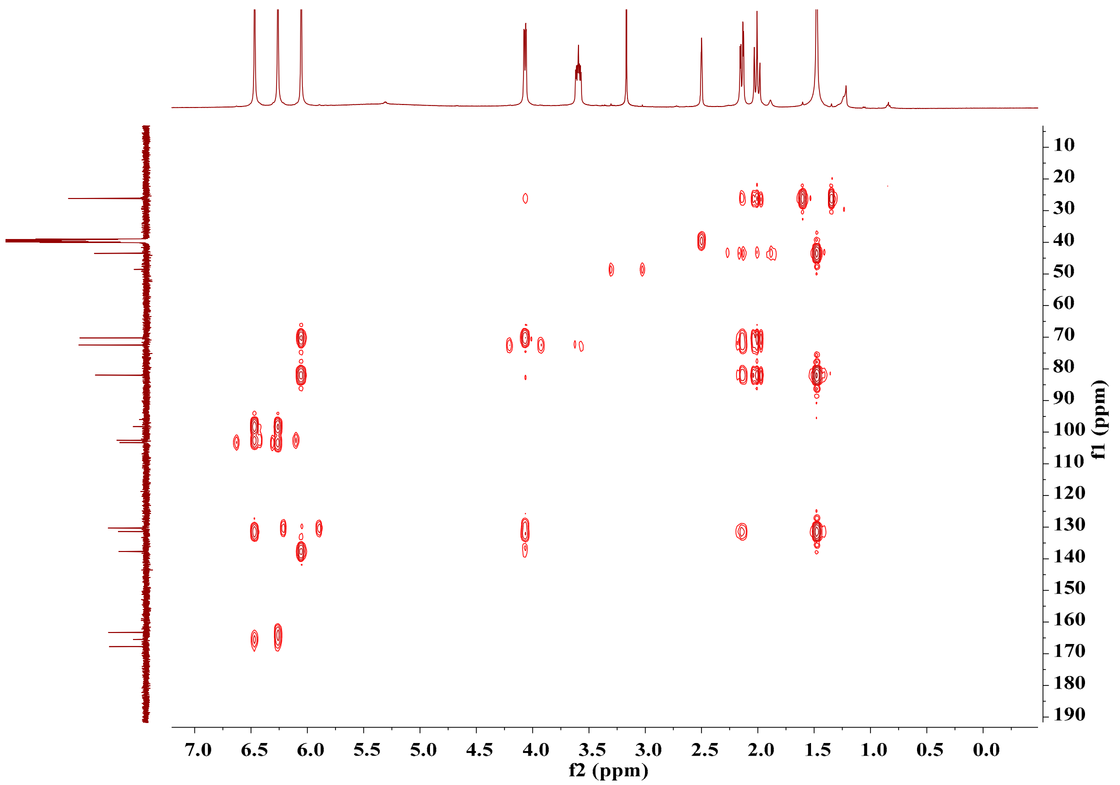

Figure S47. HMBC spectrum of (8*R*,9*R*,10a*S*)-5-hydroxyaltenuene (**14**)

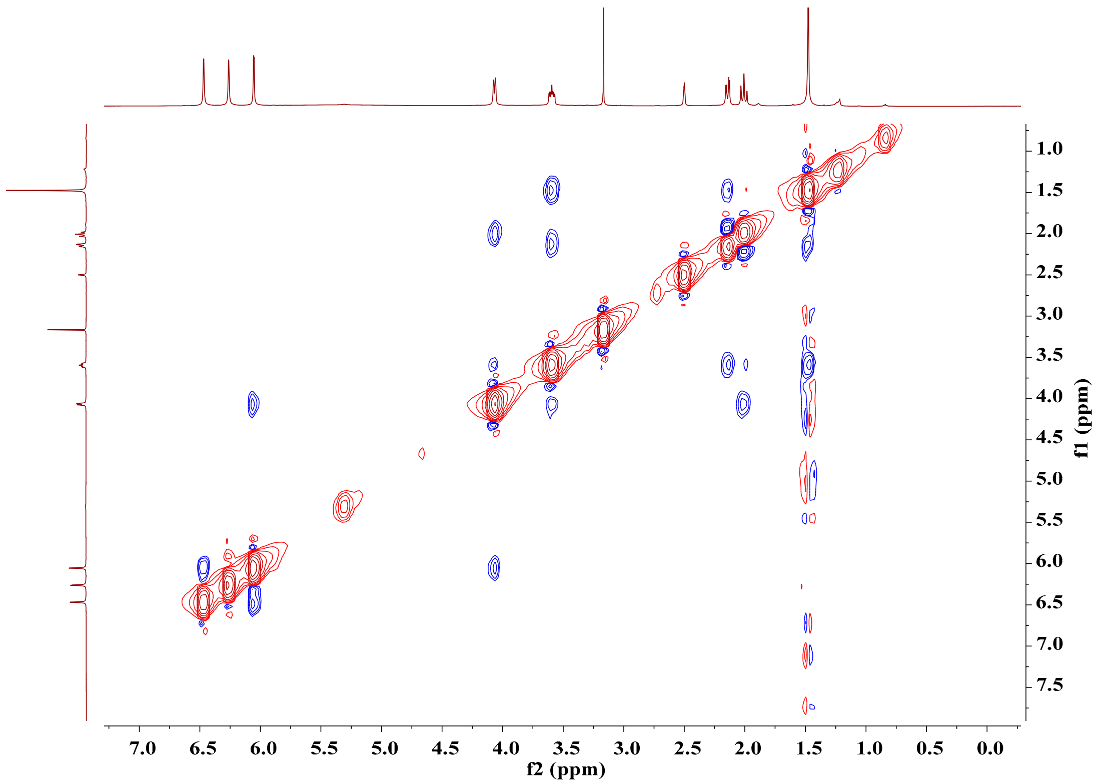

Figure S48. NOESY spectrum of (8*R*,9*R*,10a*S*)-5-hydroxyaltenuene (**14**)

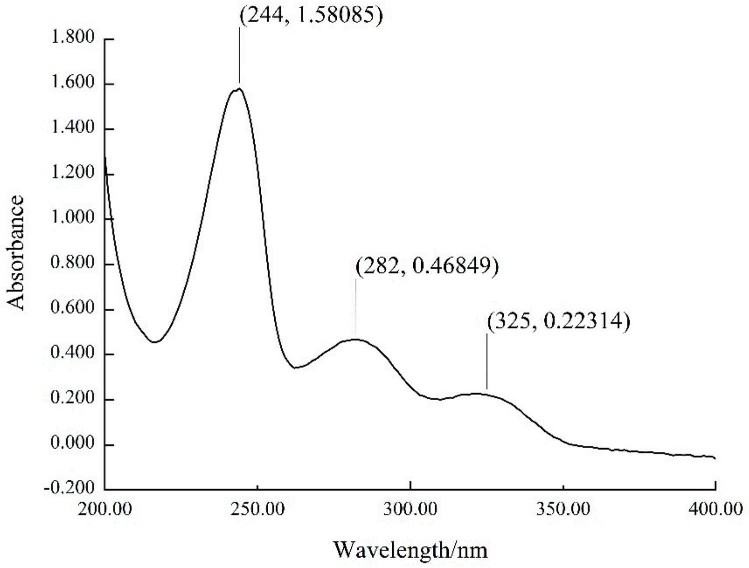

Figure S49. UV spectrum of (8*R*,9*R*,10a*S*)-5-hydroxyaltenuene (**14**)

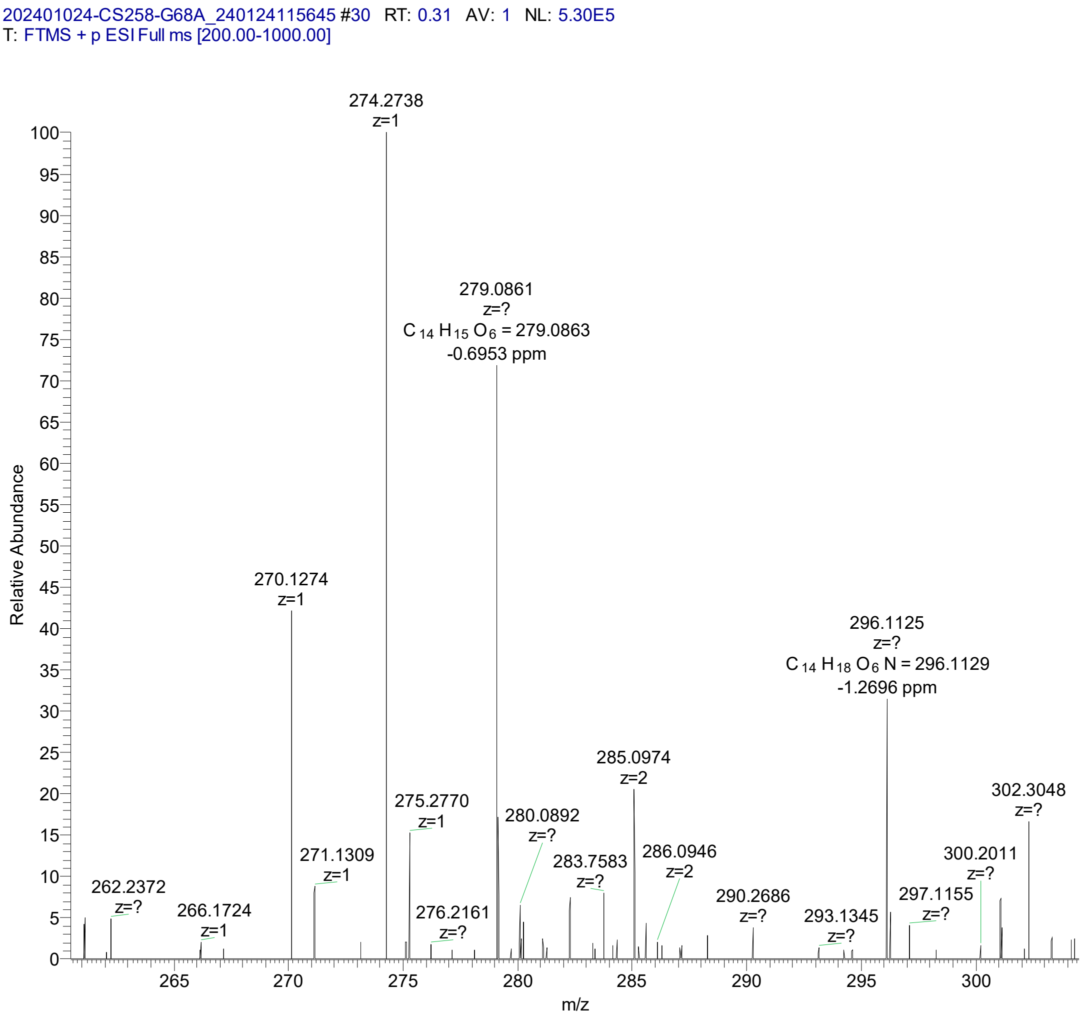

Figure S50. HRESIMS spectrum of (8*R*,9*S*,10a*R*)-5-hydroxyaltenuene (**15**)

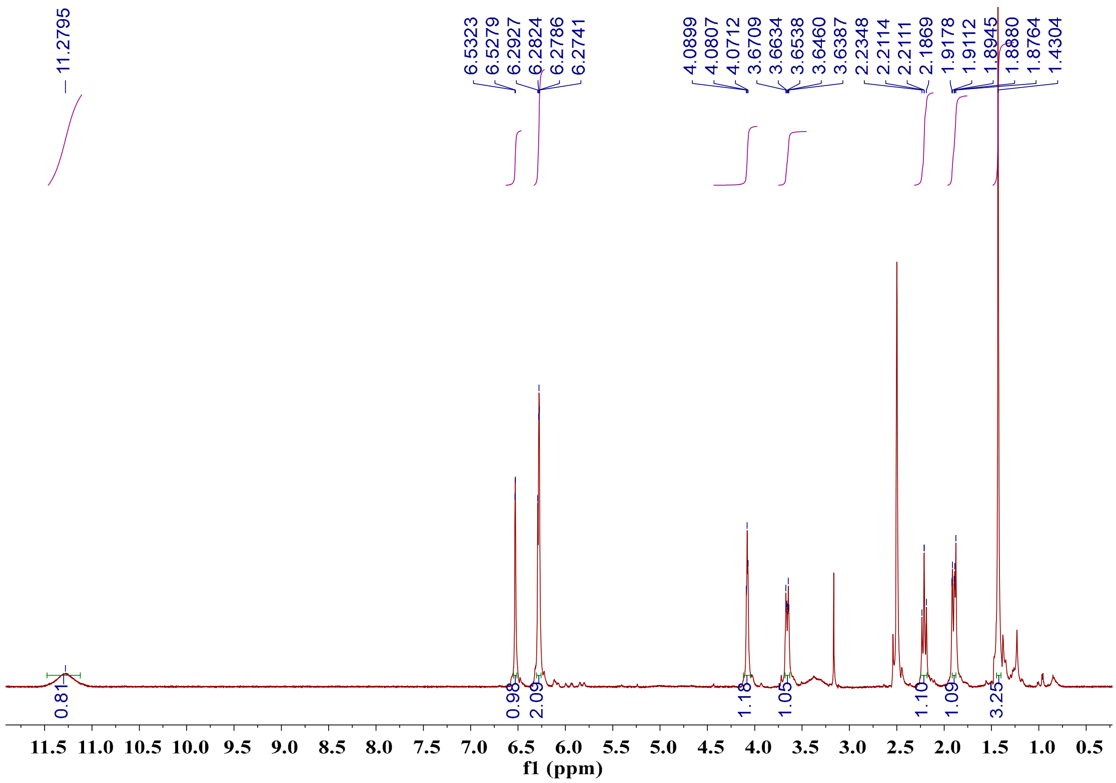

Figure S51. ^{1}H NMR spectrum (500 MHz, DMSO) of (8*R*,9*S*,10a*R*)-5-hydroxyaltenuene (**15**)

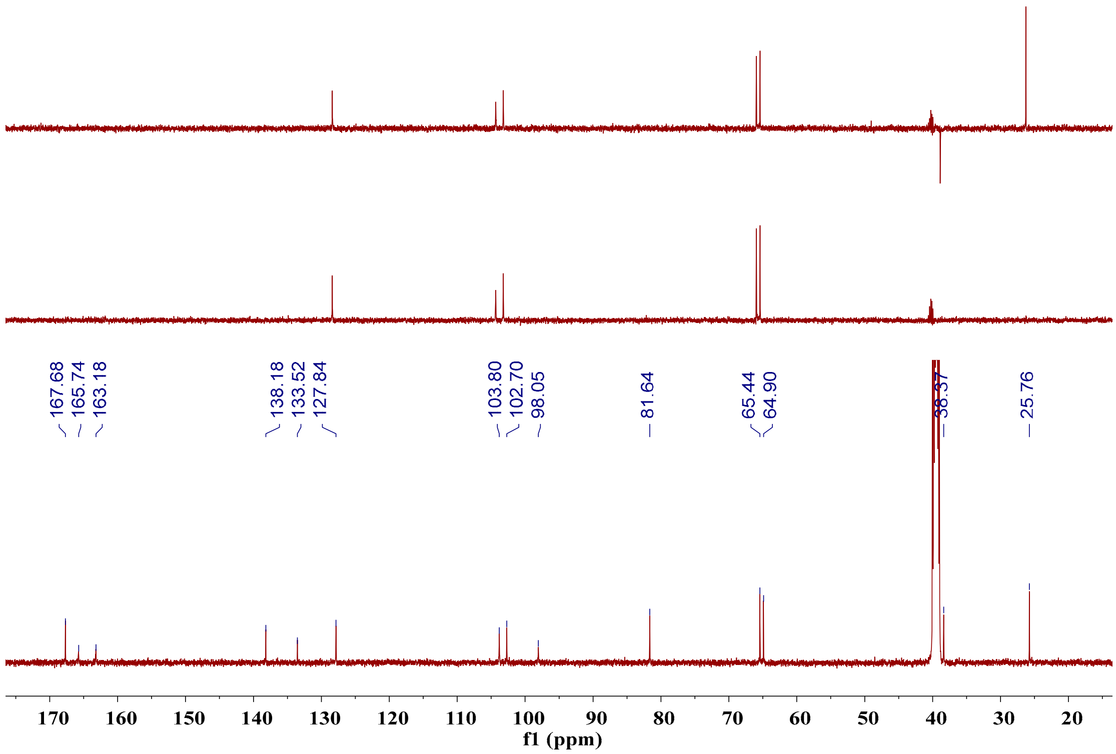

Figure S52. ^{13}C NMR spectrum (125 MHz, DMSO) of (8*R*,9*S*,10a*R*)-5-hydroxyaltenuene (**15**)

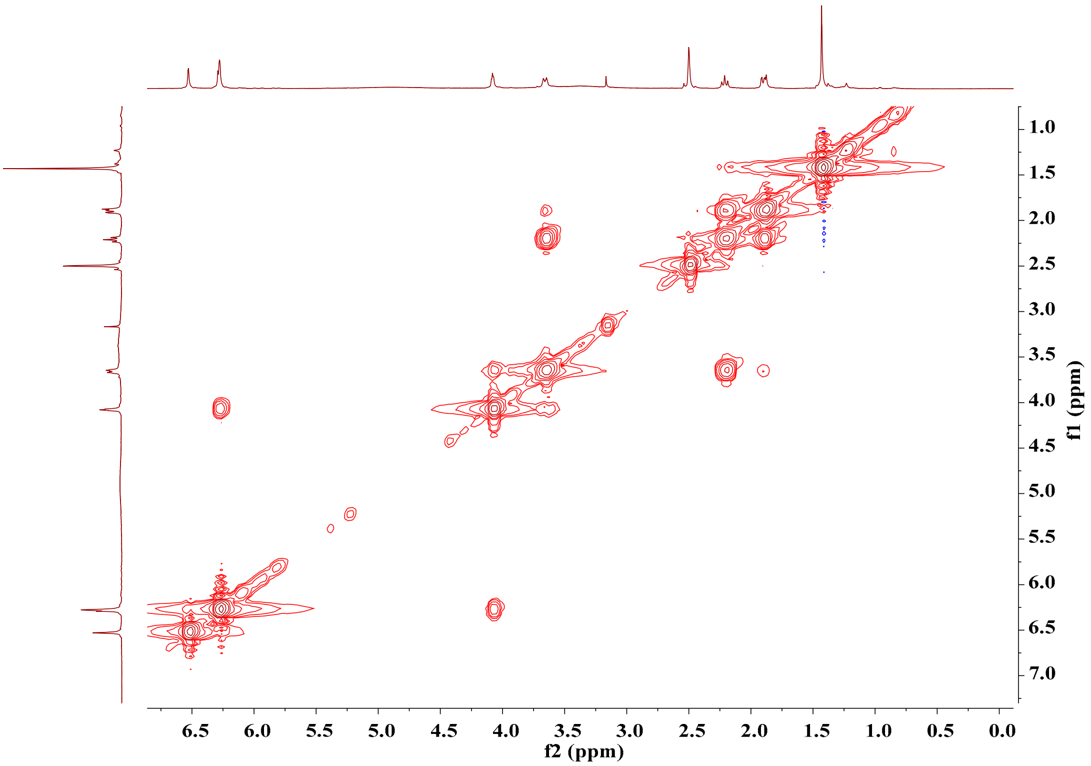

Figure S53. ^{1}H-^{1}H COSY spectrum of (8*R*,9*S*,10a*R*)-5-hydroxyaltenuene (**15**)

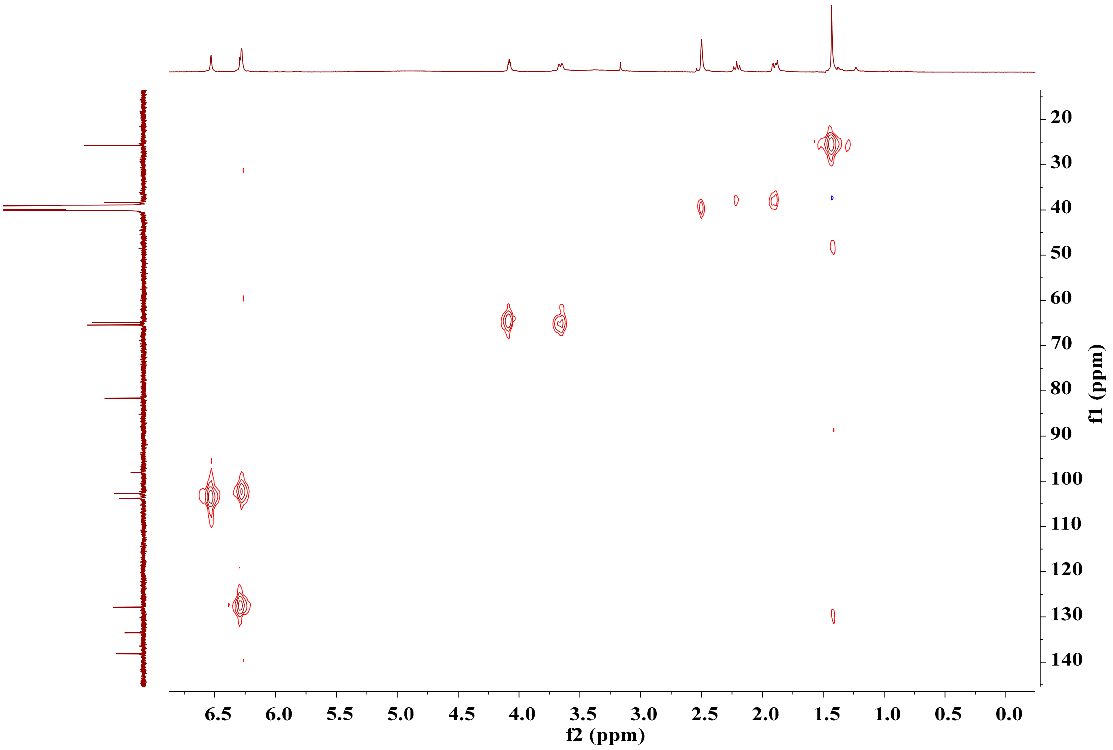

Figure S54. HSQC spectrum of (8*R*,9*S*,10a*R*)-5-hydroxyaltenuene (**15**)

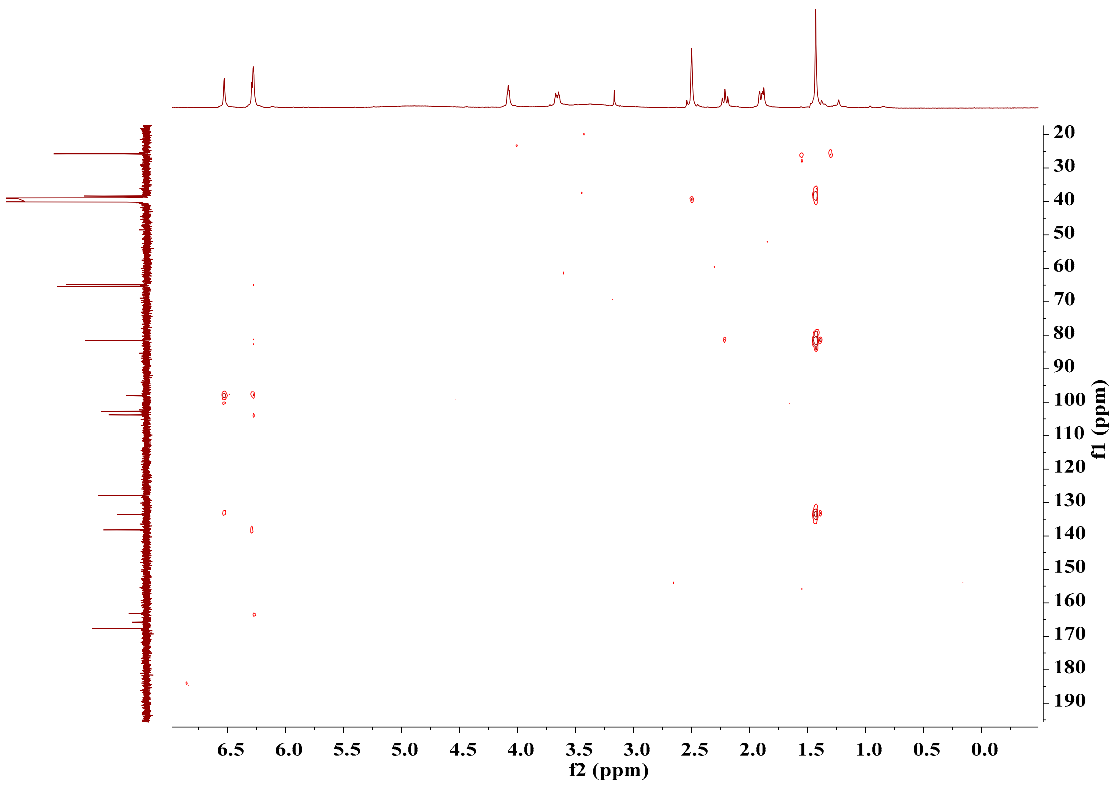

Figure S55. HMBC spectrum of (8*R*,9*S*,10a*R*)-5-hydroxyaltenuene (**15**)

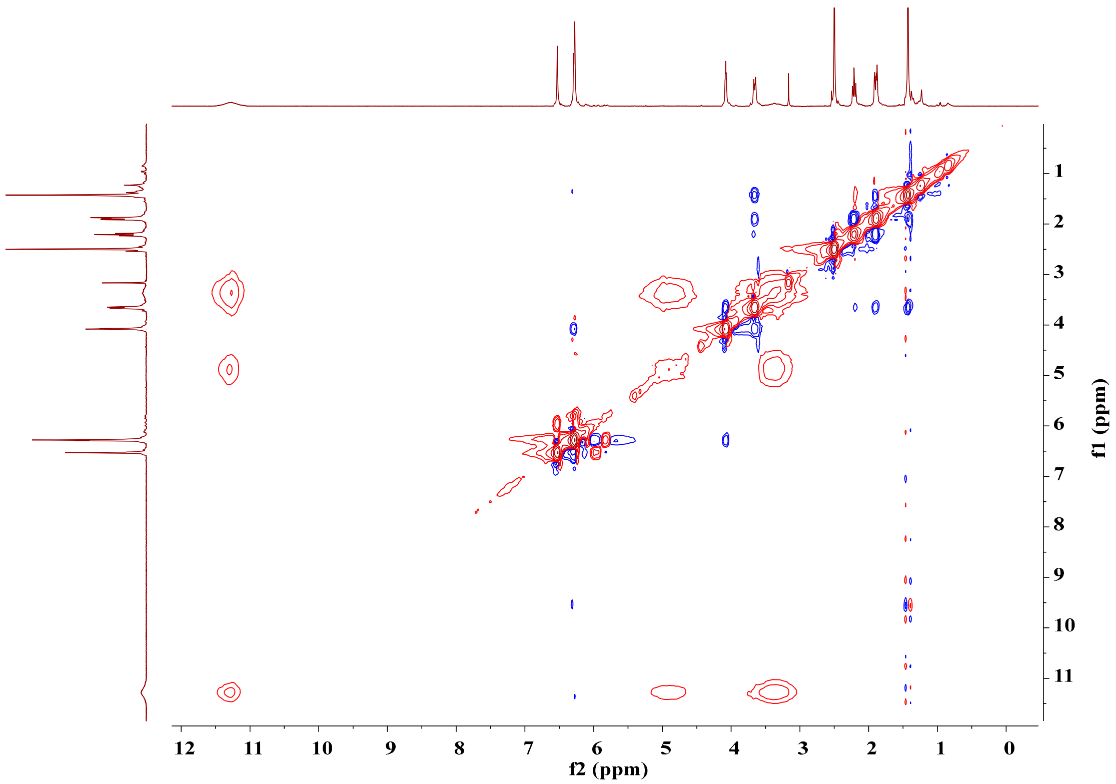

Figure S56. NOESY spectrum of (8*R*,9*S*,10a*R*)-5-hydroxyaltenuene (**15**)

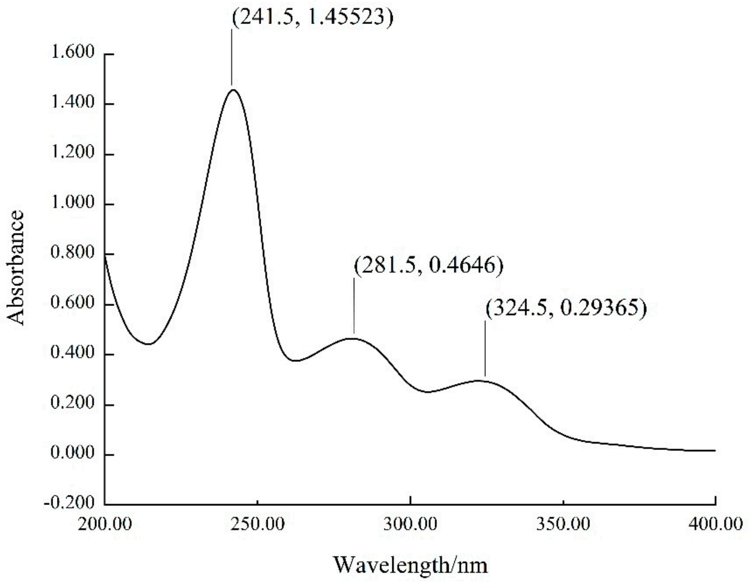

Figure S57. UV spectrum of (8*R*,9*S*,10a*R*)-5-hydroxyaltenuene (**15**)

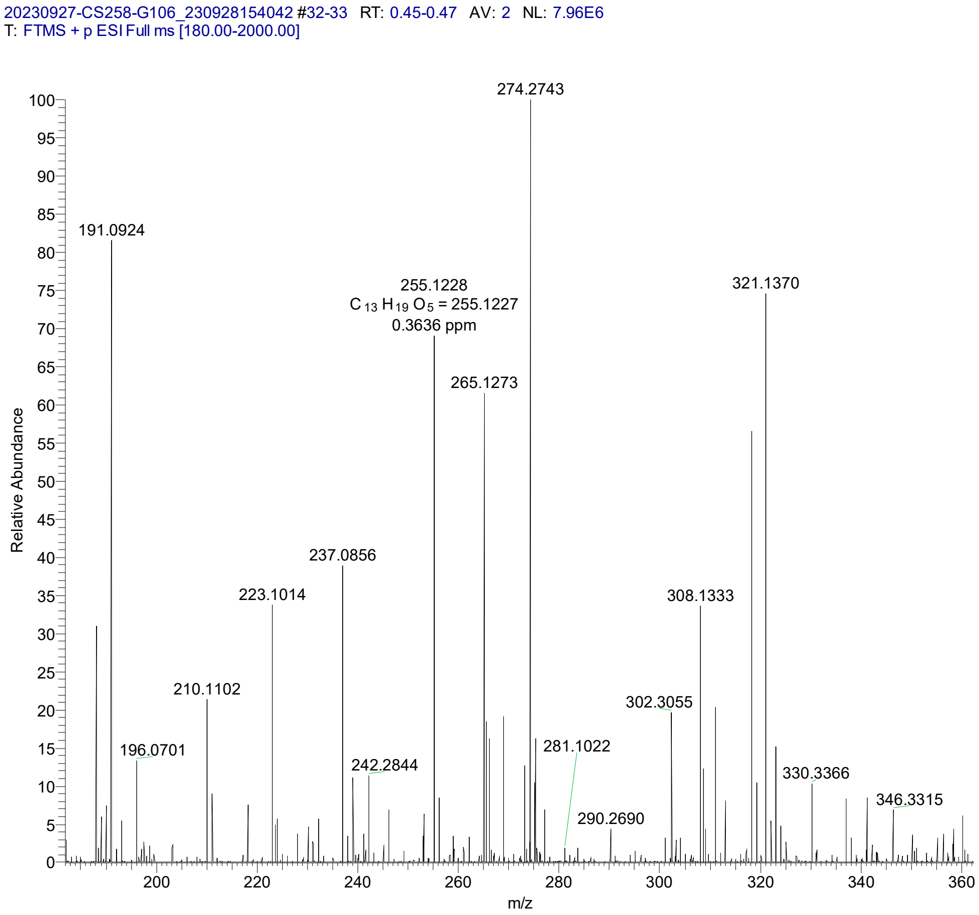

Figure S58. HRESIMS spectrum of nemanecin D (**25**)

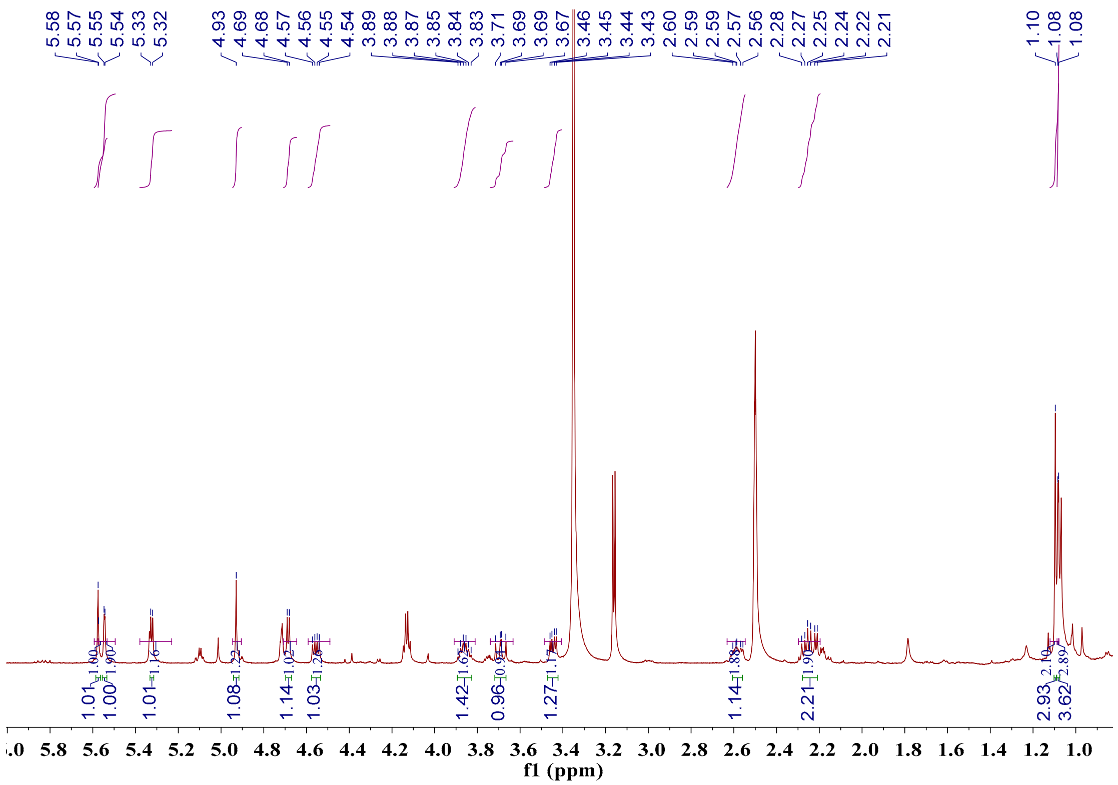

Figure S59. ^{1}H NMR spectrum (500 MHz, DMSO) of nemanecin D (**25**)

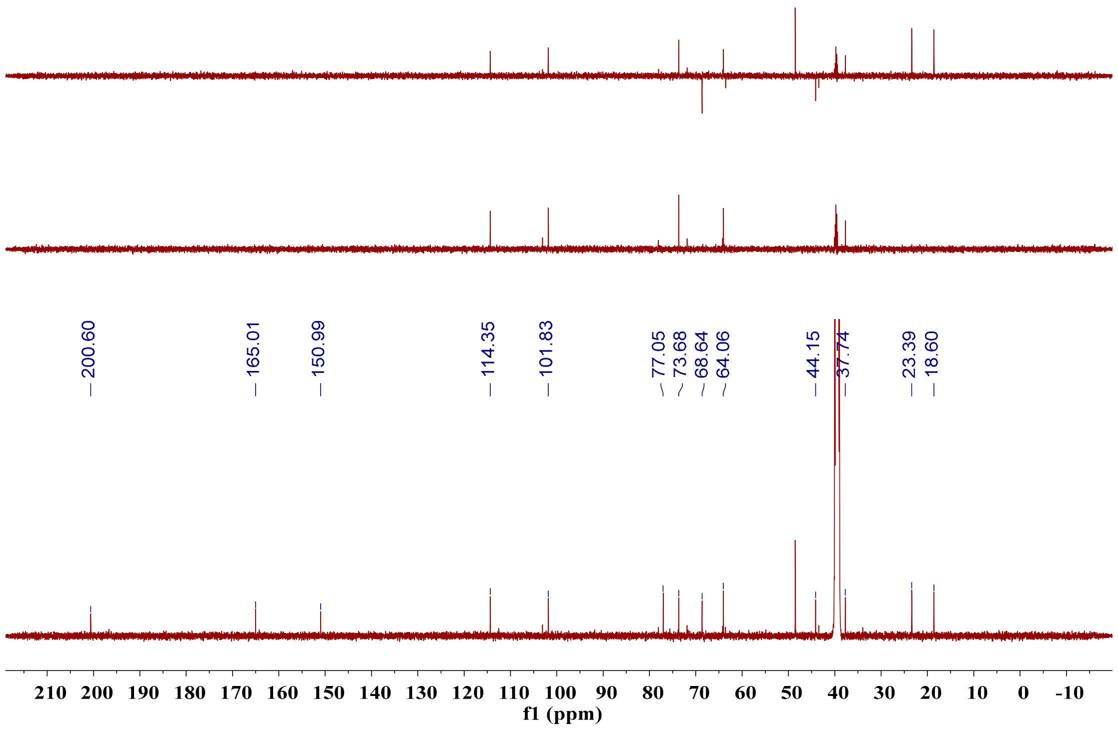

Figure S60. ^{13}C NMR spectrum (125 MHz, DMSO) of nemanecin D (**25**)

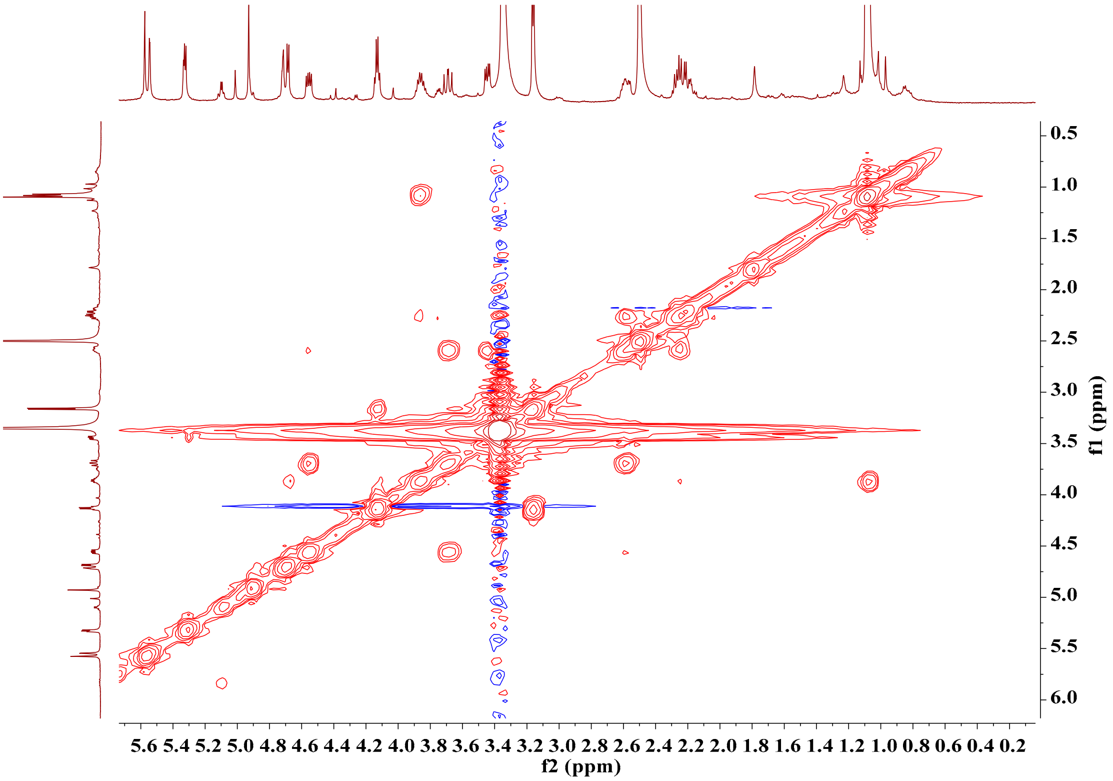

Figure S61. ^{1}H-^{1}H COSY spectrum of nemanecin D (**25**)

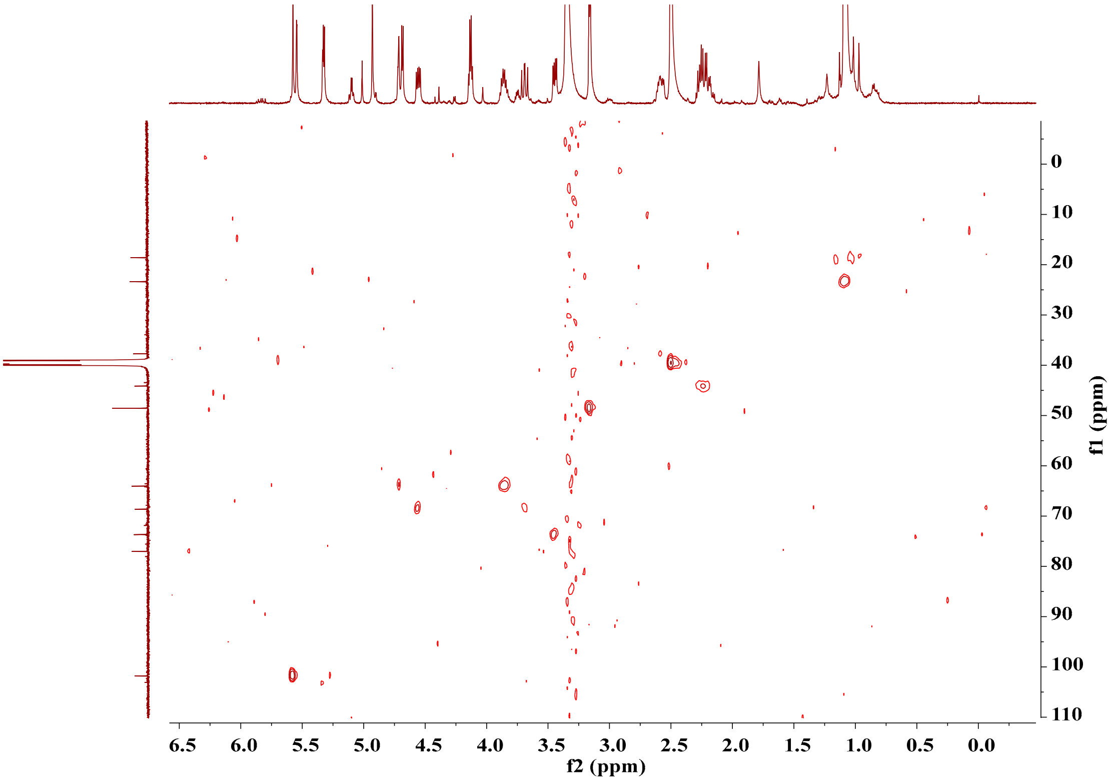

Figure S62. HSQC spectrum of nemanecin D (**25**)

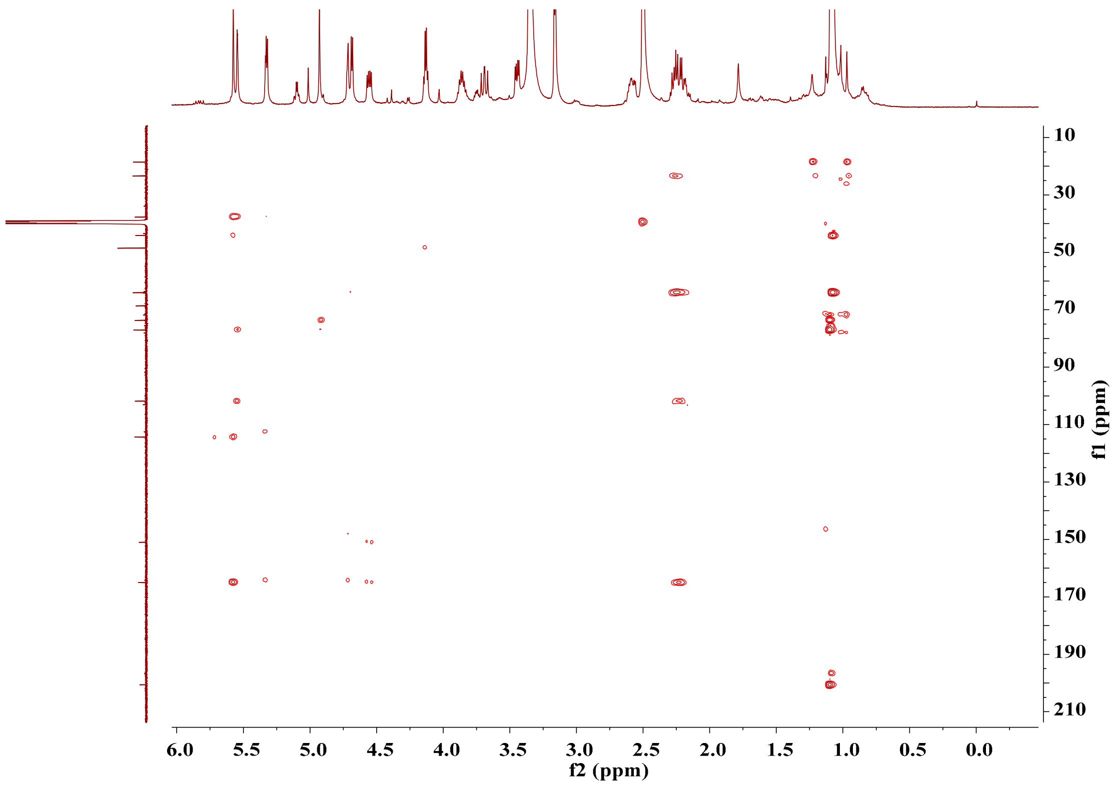

Figure S63. HMBC spectrum of nemanecin D (**25**)

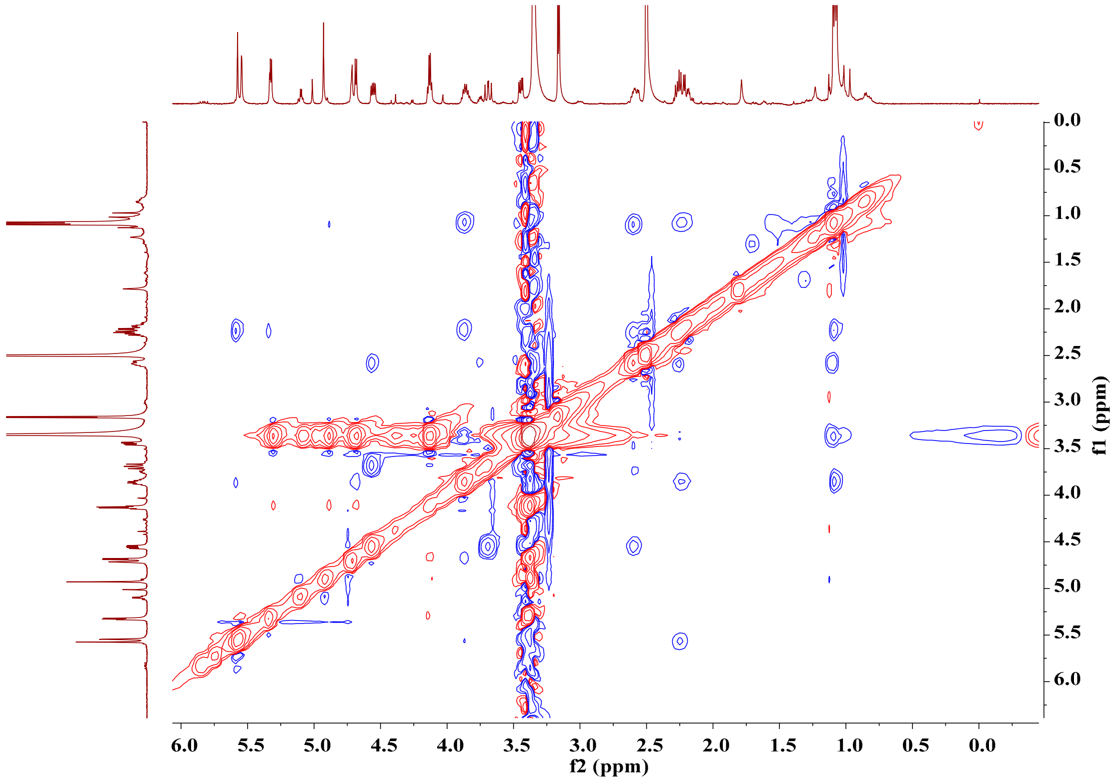

Figure S64. NOESY spectrum of nemanecin D (**25**)

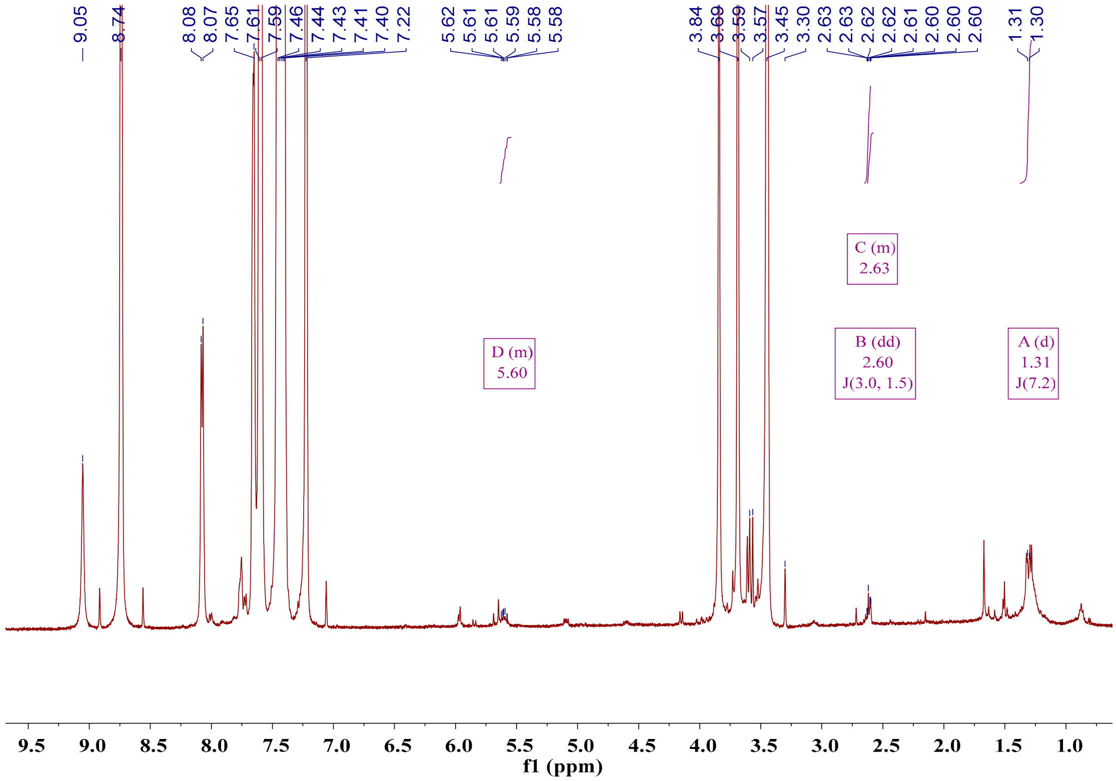

Figure S65. ^{1}H NMR spectrum of (*S*)-MTPA ester (**25a**)

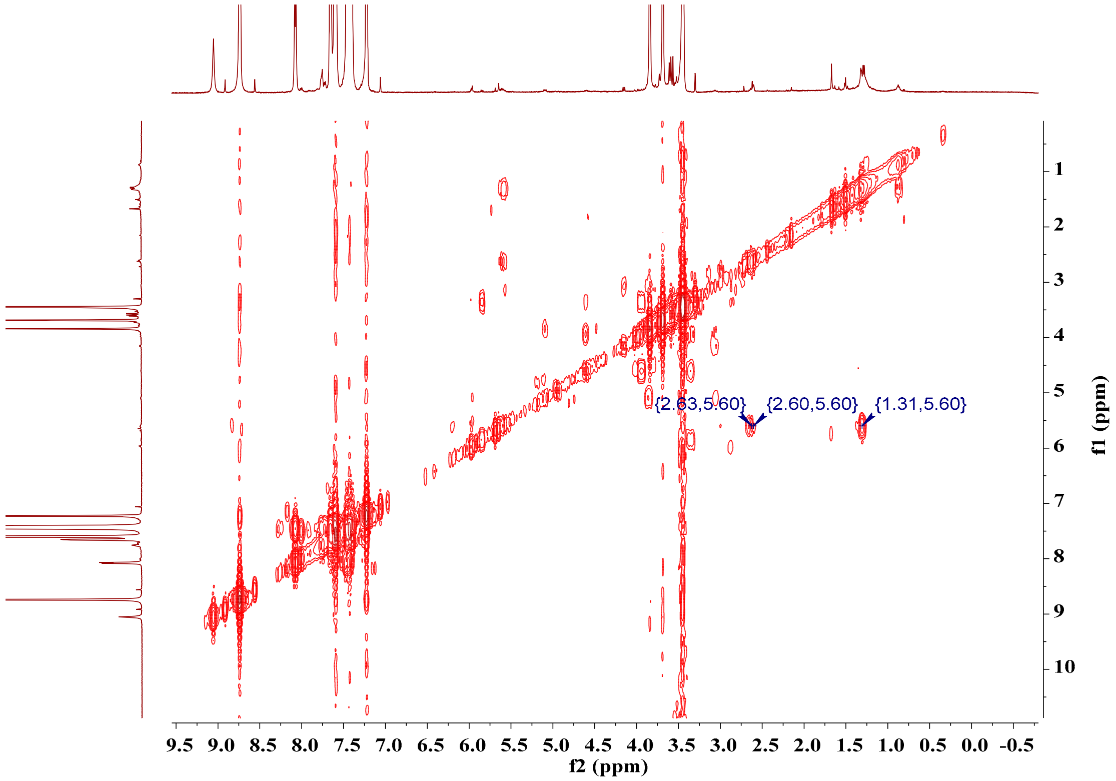

Figure S66. ^{1}H-^{1}H COSY spectrum of (*S*)-MTPA ester (**25a**)

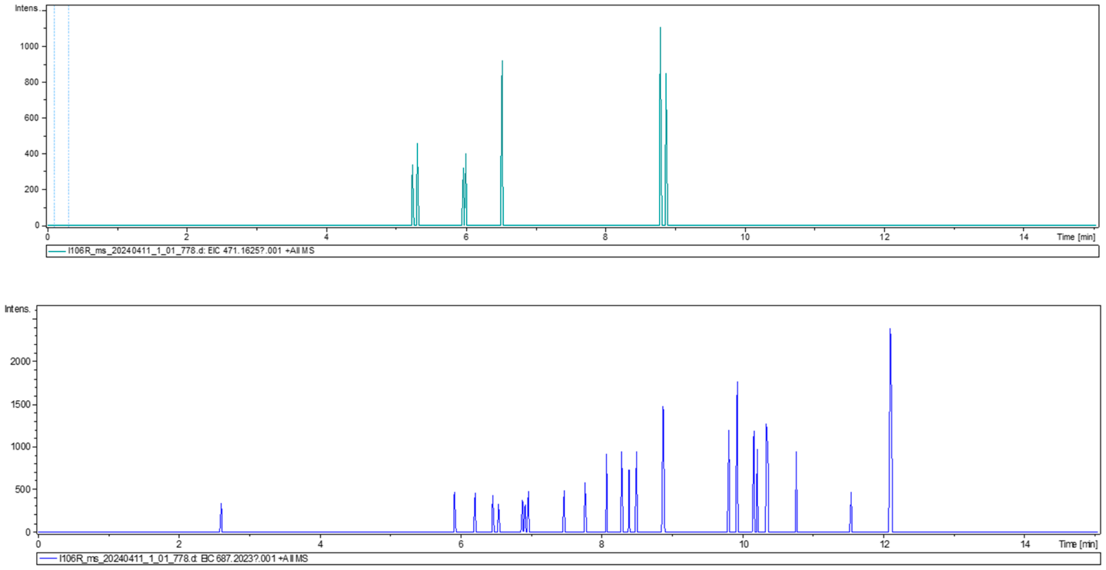

Failed to find the MS peak due to instability

Figure S67. HRMS spectrum of (*S*)-MTPA ester (**25a**)

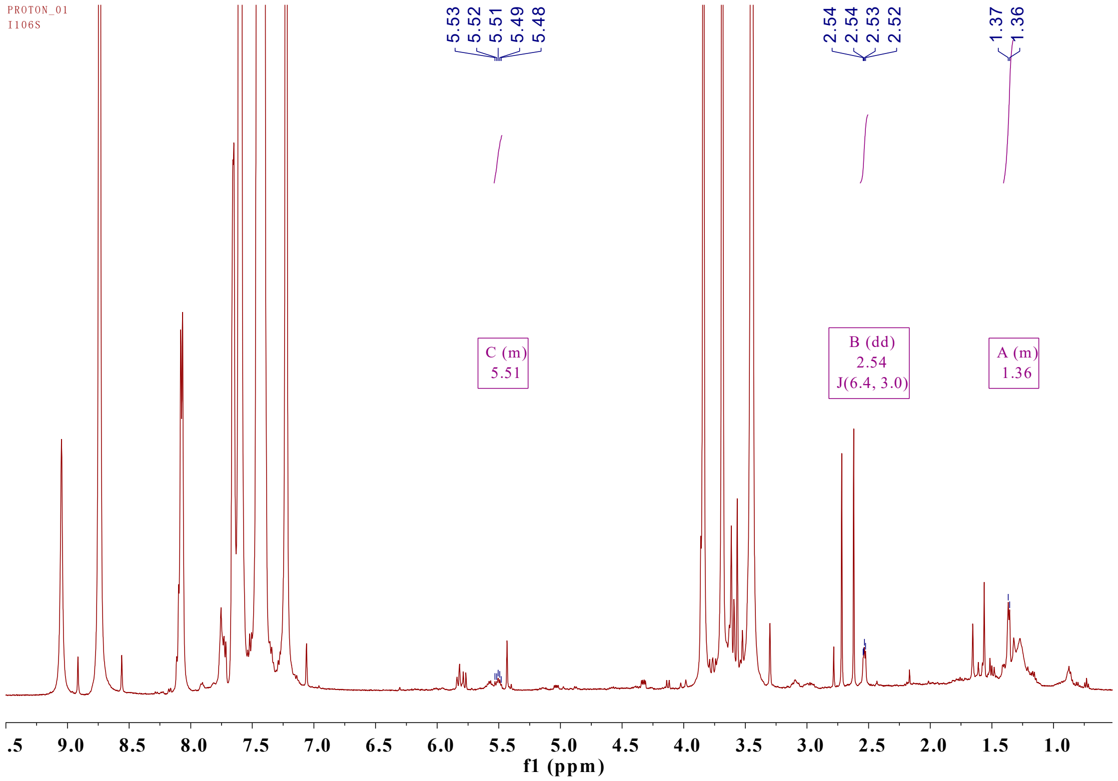

Figure S68. ^{1}H NMR spectrum of (*R*)-MTPA ester (**25b**)

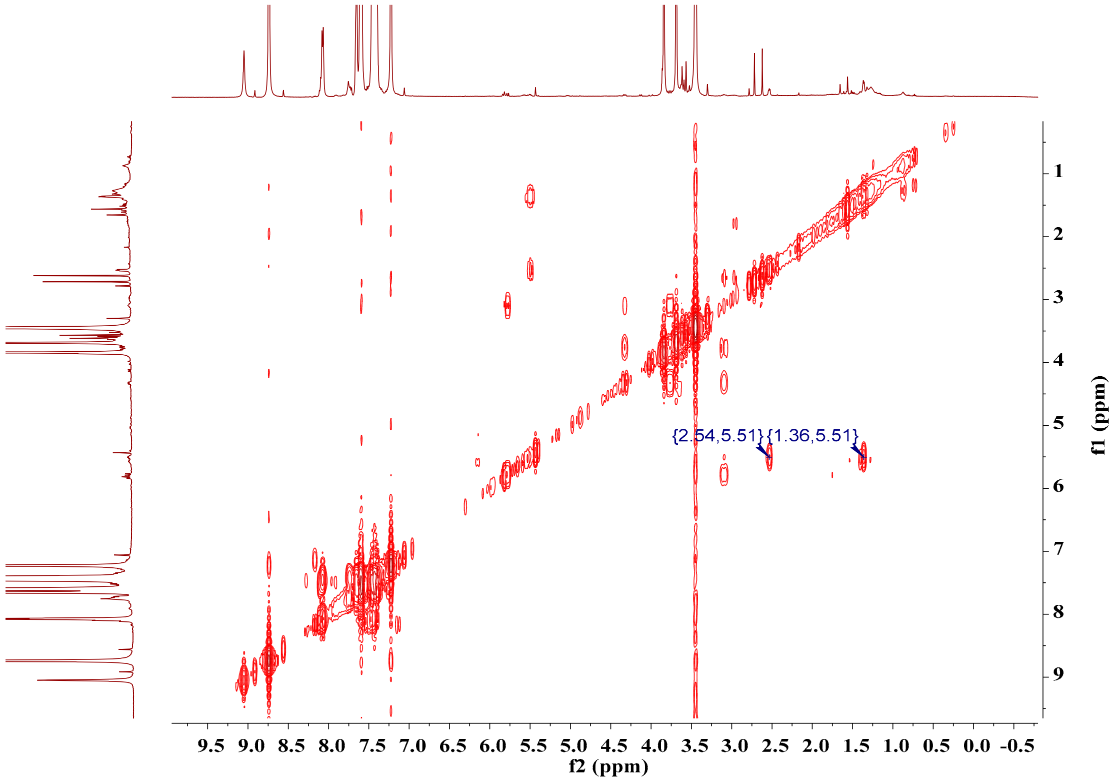

Figure S69. ^{1}H-^{1}H COSY spectrum of (*R*)-MTPA ester (**25b**)

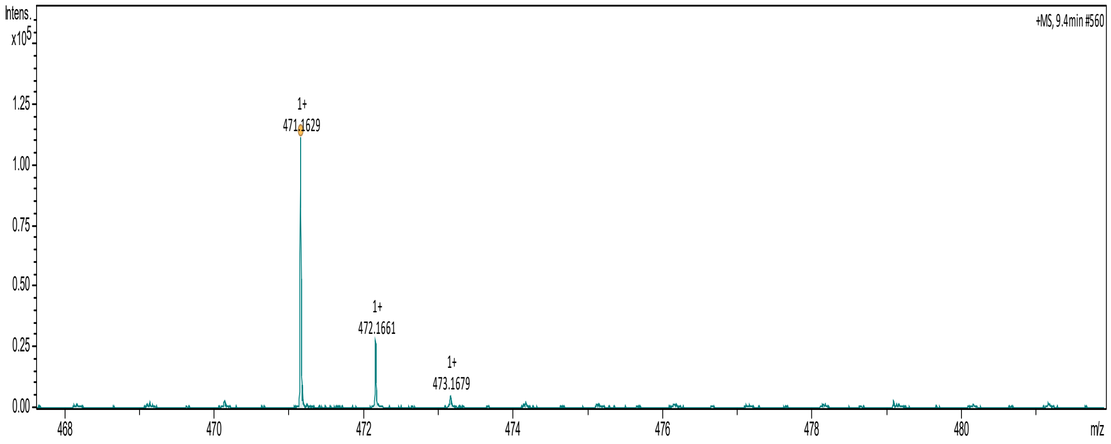

Meas. m/z	z	Ion Formula	m/z	err [mDa]	err [ppm]	mSigma	rdb	Adduct	Sum Formula
471.162861	1	$C_{23}H_{26}F_{3}O_{7}$	471.162514	-0.3	-0.7	0.3	9.5	M+H	$C_{23}H_{25}F_{3}O_{7}$

Figure S70. HRMS spectrum of (*R*)-MTPA ester (**25b**)

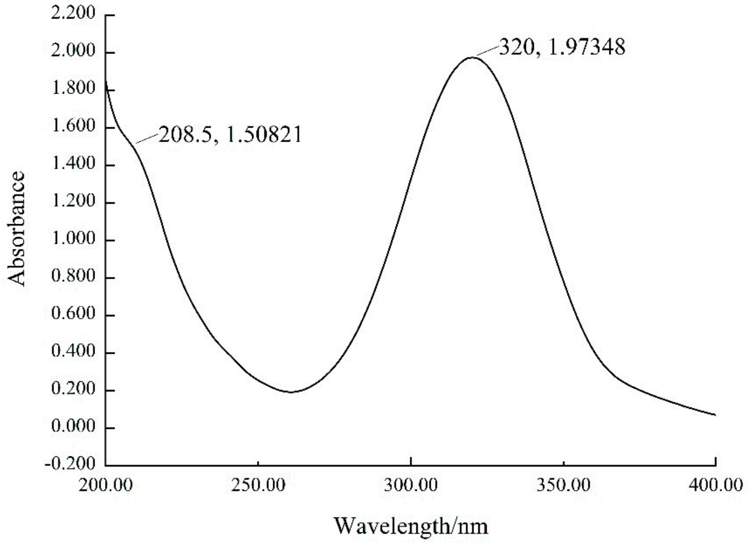

Figure S71. UV spectrum of nemanecin D (**25**)

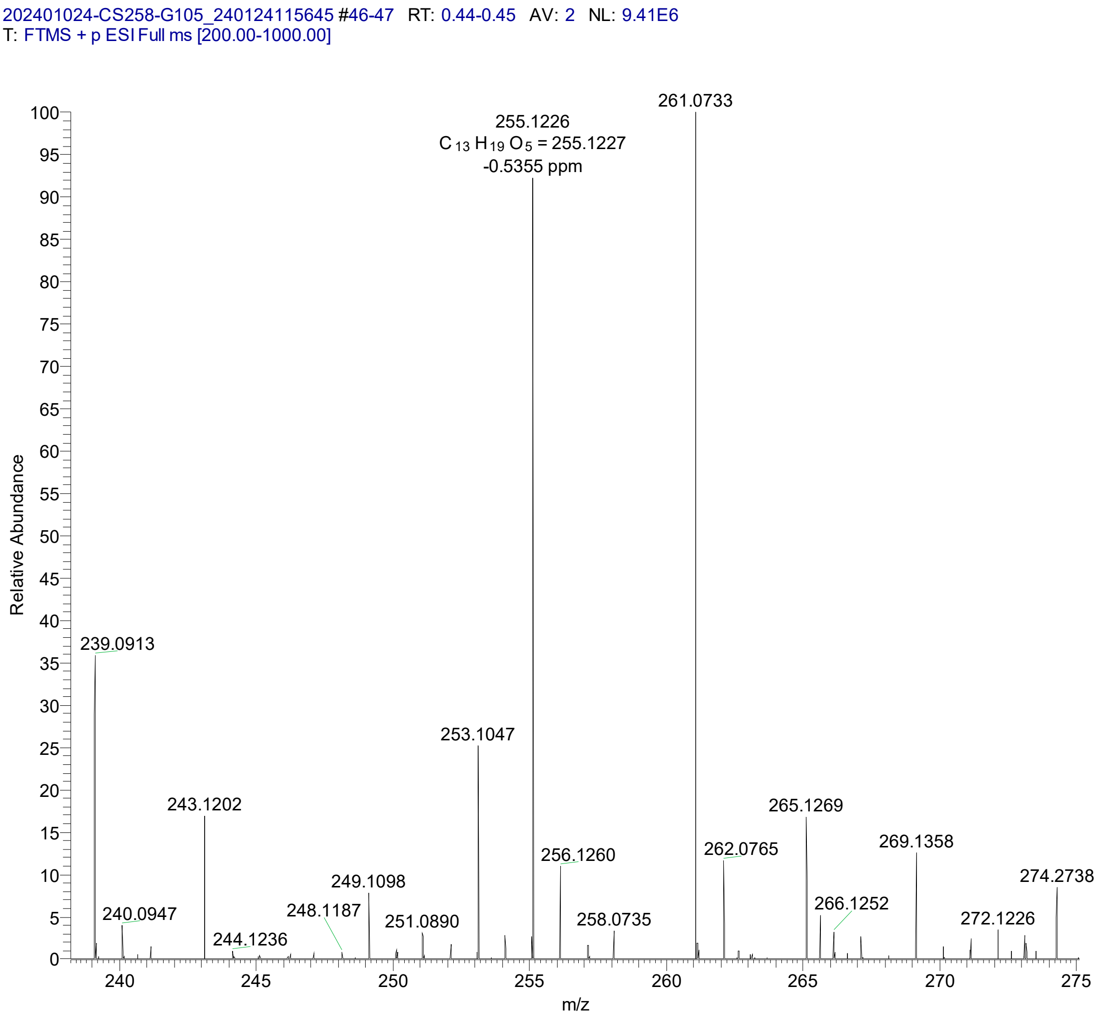

Figure S72. HRESIMS spectrum of nemanecin E (**26**)

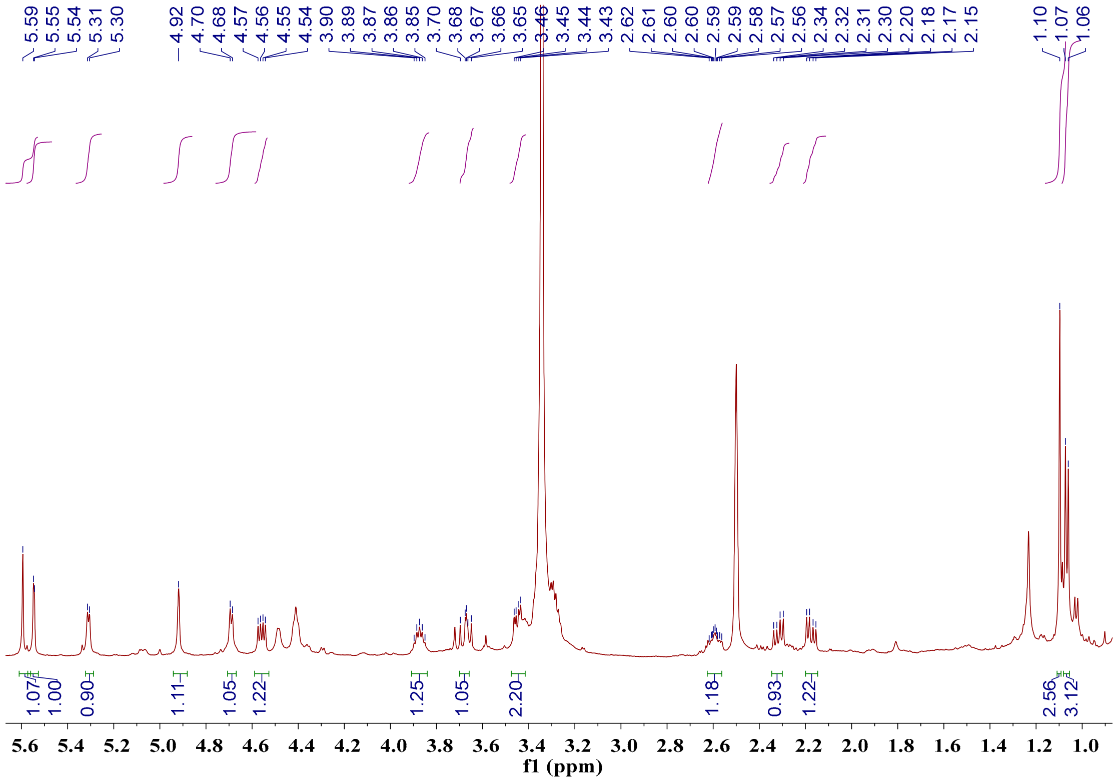

Figure S73. ^{1}H NMR spectrum (500 MHz, DMSO) of nemanecin E (**26**)

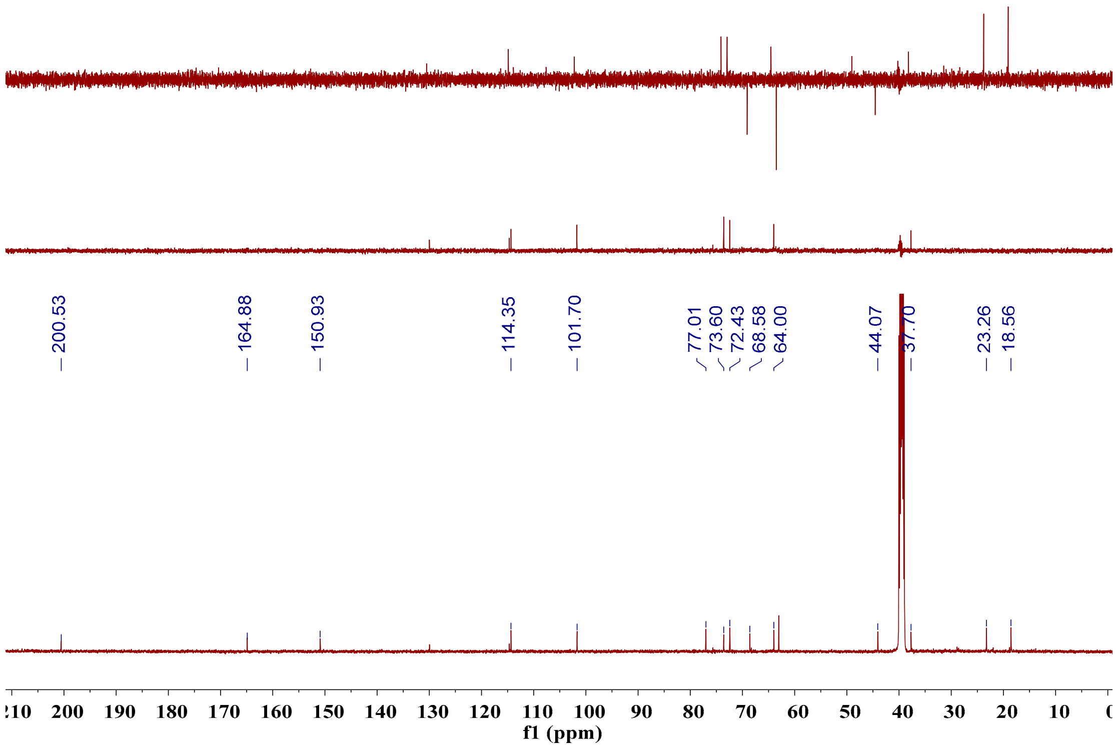

Figure S74. ^{13}C NMR spectrum (125 MHz, DMSO) of nemanecin E (**26**)

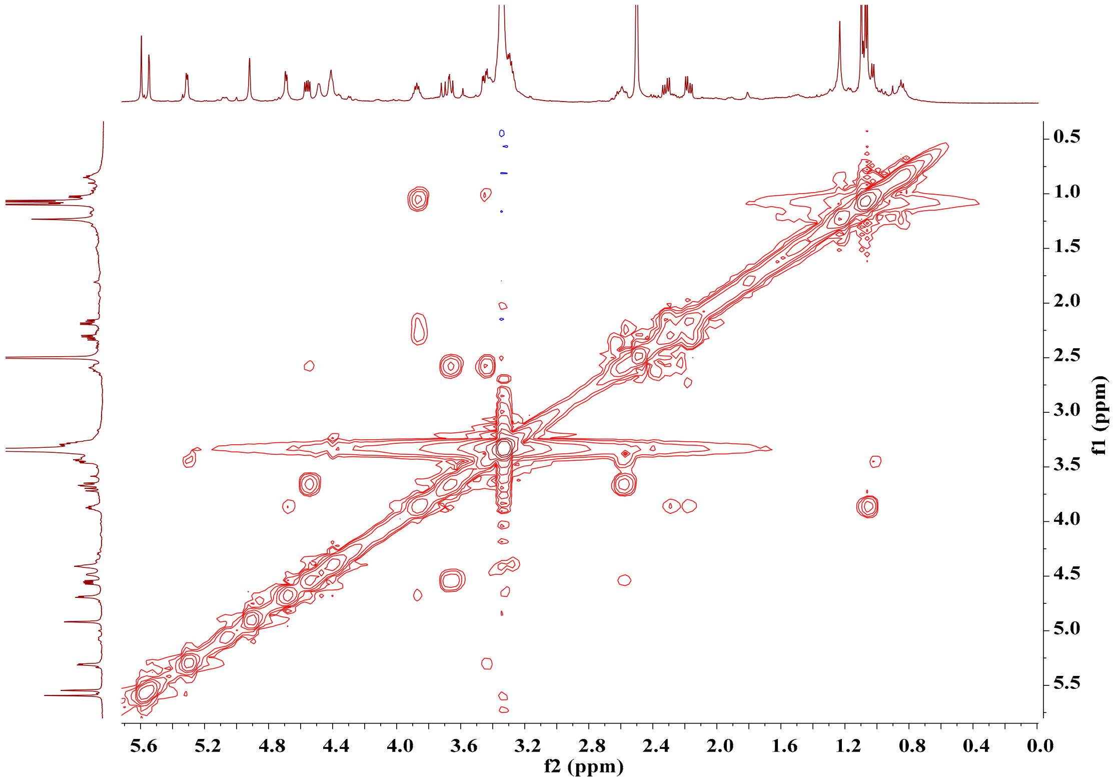

Figure S75. ^{1}H-^{1}H COSY spectrum of nemanecin E (**26**)

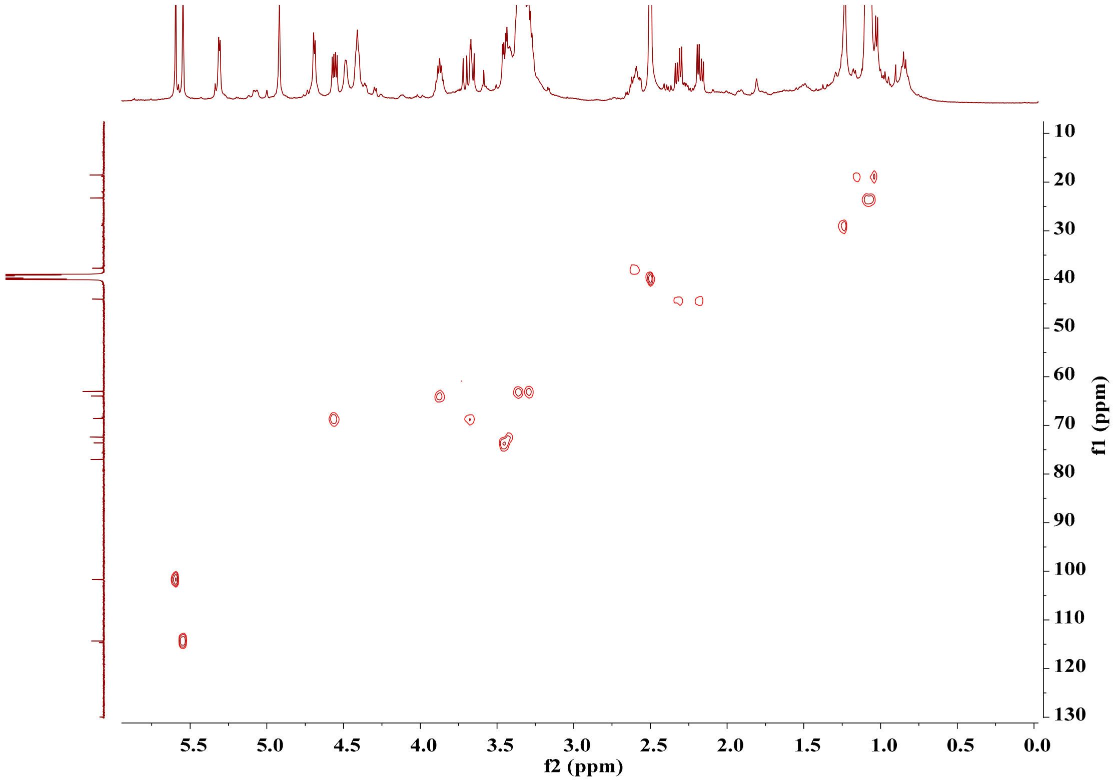

Figure S76. HSQC spectrum of nemanecin E (**26**)

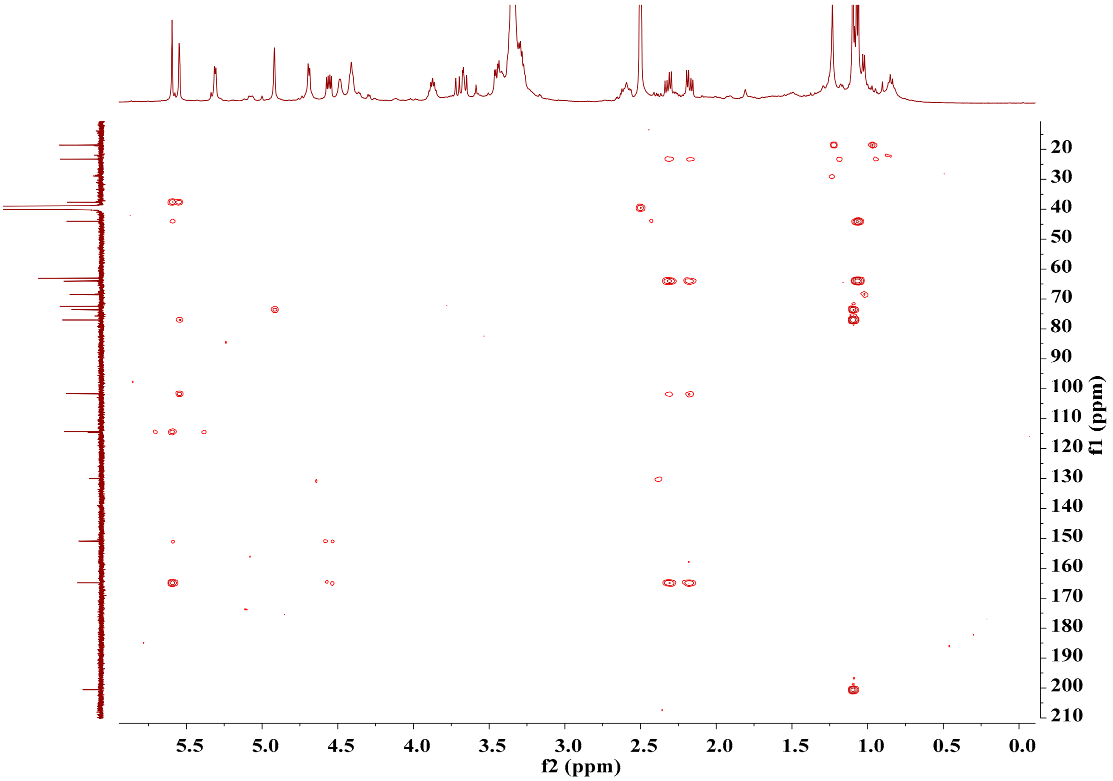

Figure S77. HMBC spectrum of nemanecin E (**26**)

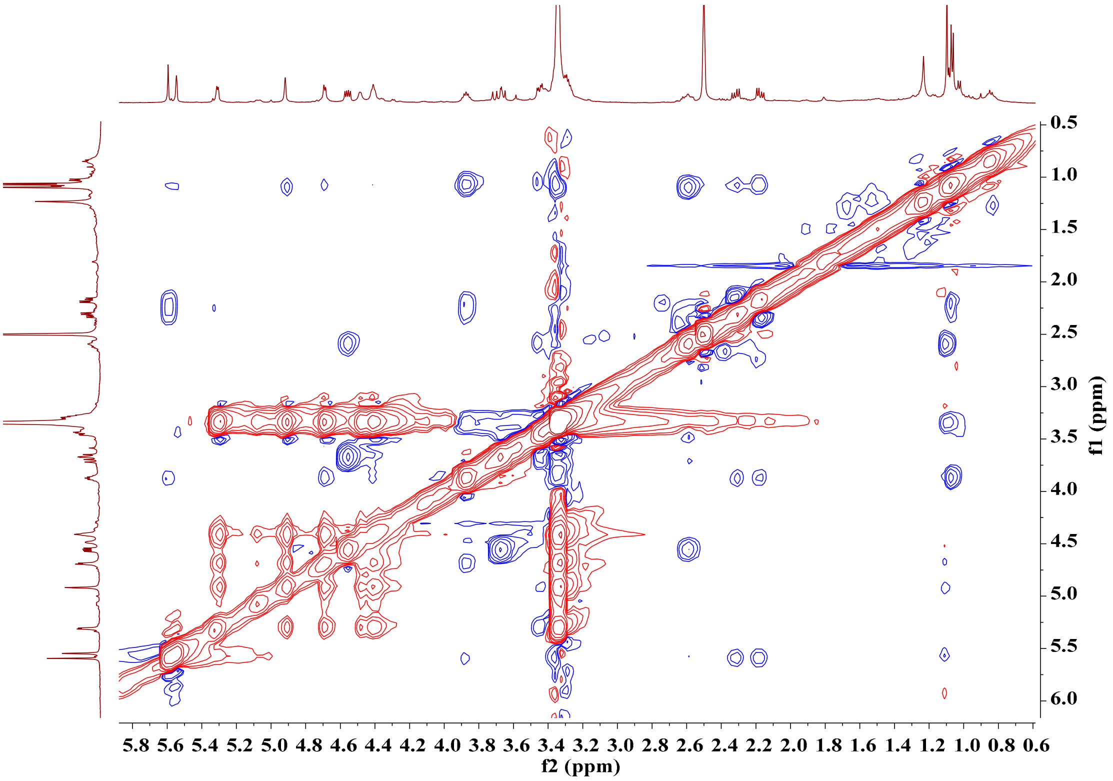

Figure S78. NOESY spectrum of nemanecin E (**26**)

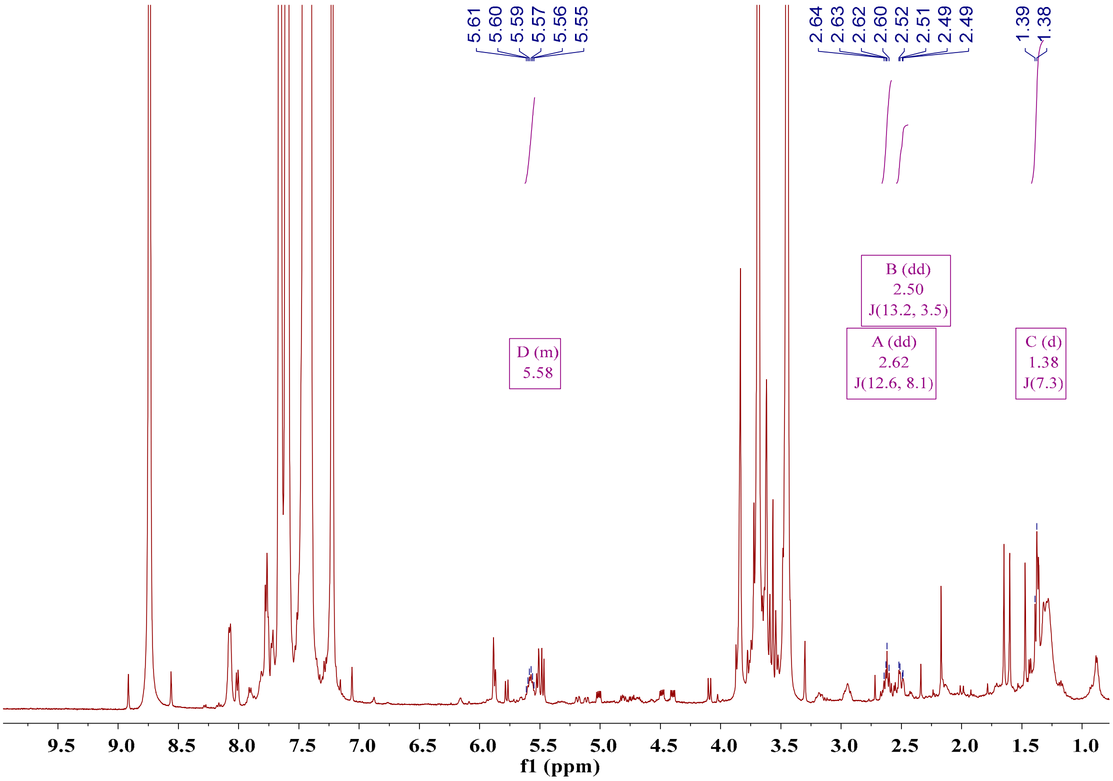

Figure S79. ^{1}H NMR spectrum of (*S*)-MTPA Ester (**26a**)

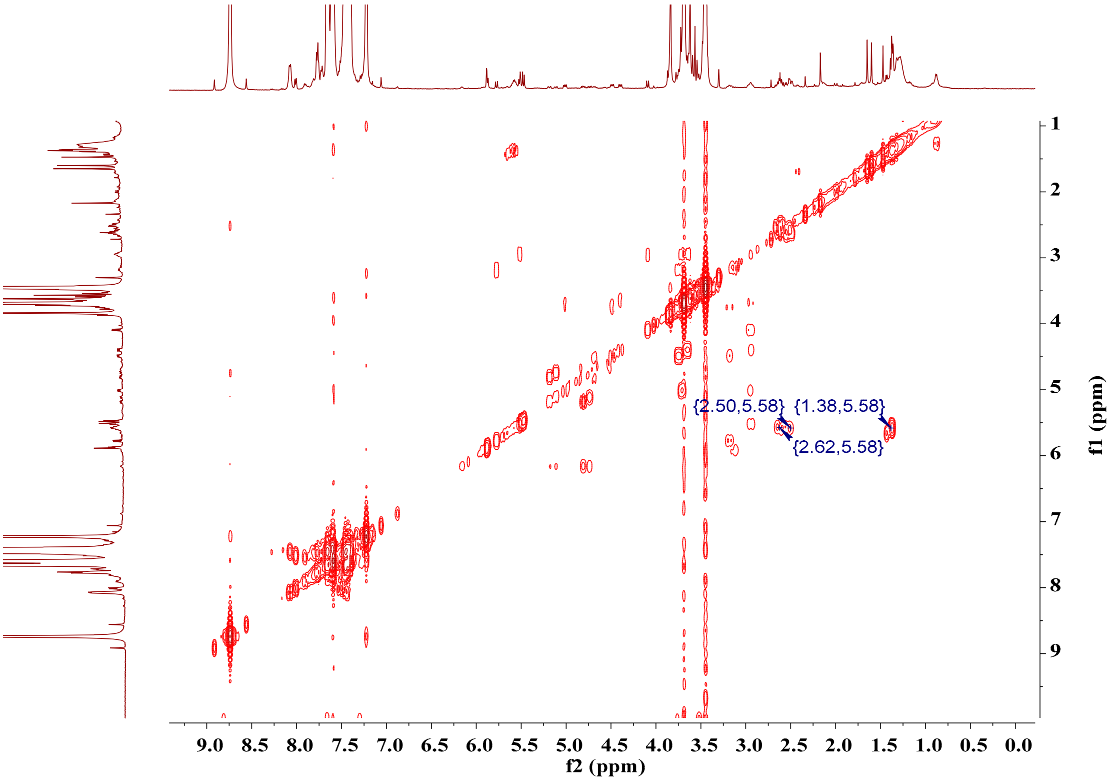

Figure S80. ^{1}H-^{1}H COSY spectrum of (*S*)-MTPA Ester (**26a**)

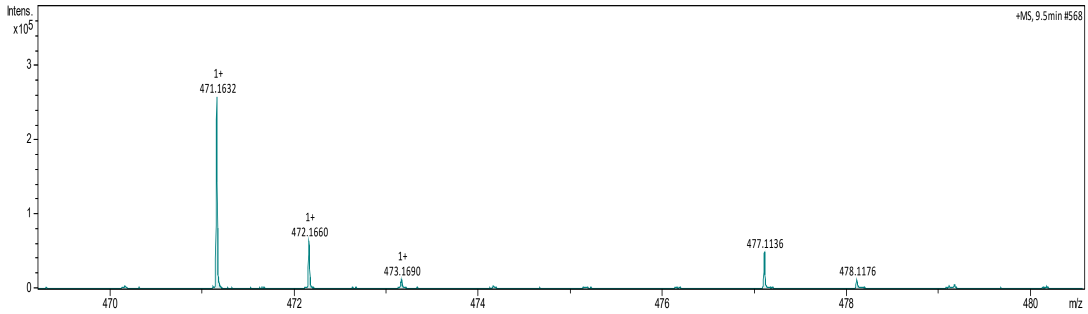

Meas. m/z	z	Ion Formula	m/z	err [mDa]	err [ppm]	mSigma	rdb	Adduct	Sum Formula
471.163243	1	$C_{23}H_{26}F_3O_7$	471.162514	-0.7	-1.5	4.4	9.5	M+H	$C_{23}H_{25}F_3O_7$

Figure S81. HRMS spectrum of (*S*)-MTPA Ester (**26a**)

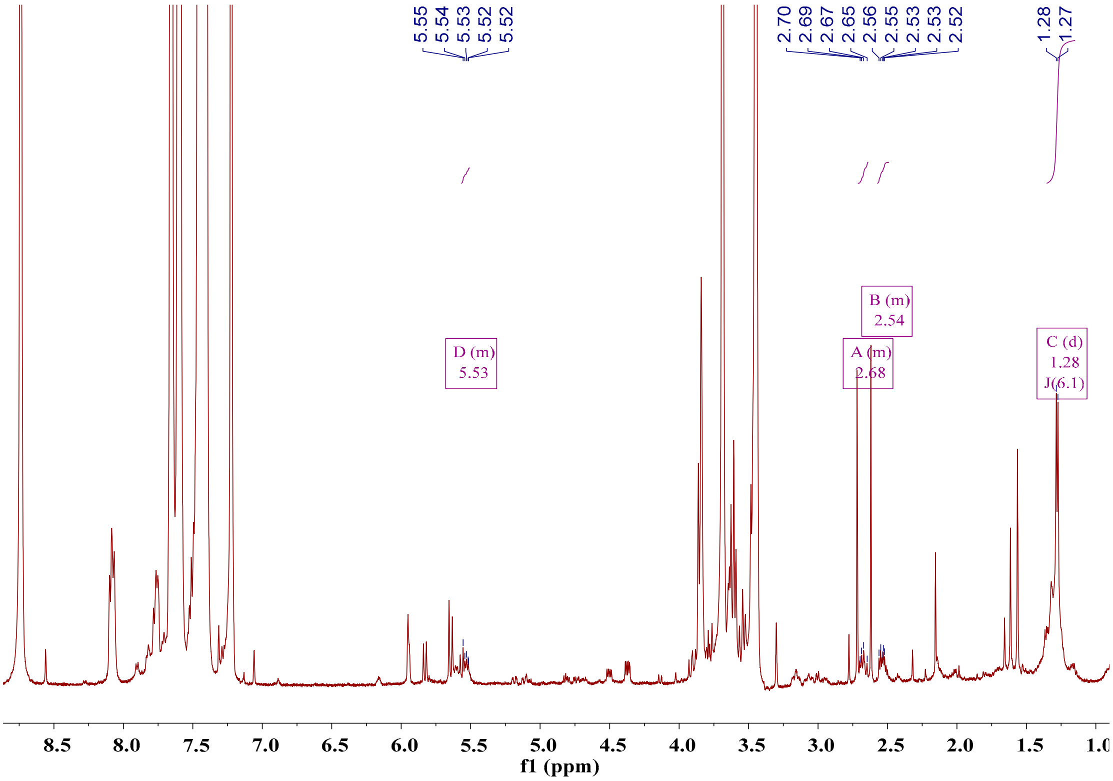

Figure S82. ^{1}H NMR spectrum of (*R*)-MTPA Ester (**26b**)

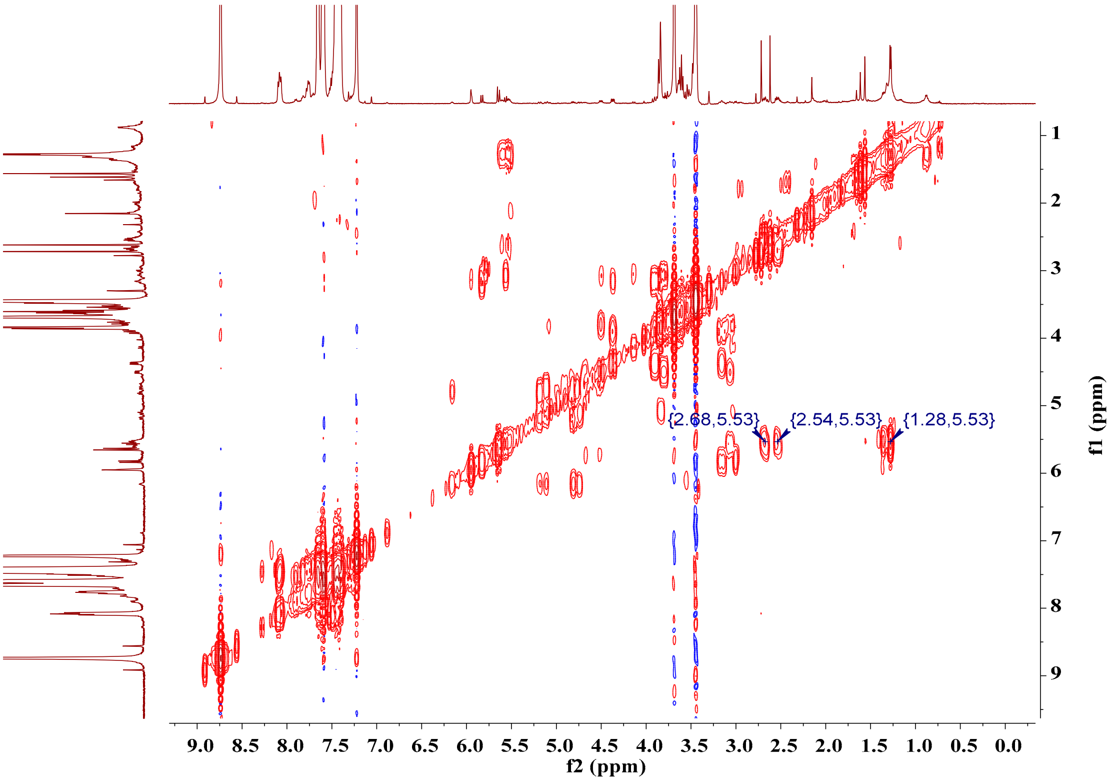

Figure S83. ^{1}H-^{1}H COSY spectrum of (*R*)-MTPA Ester (**26b**)

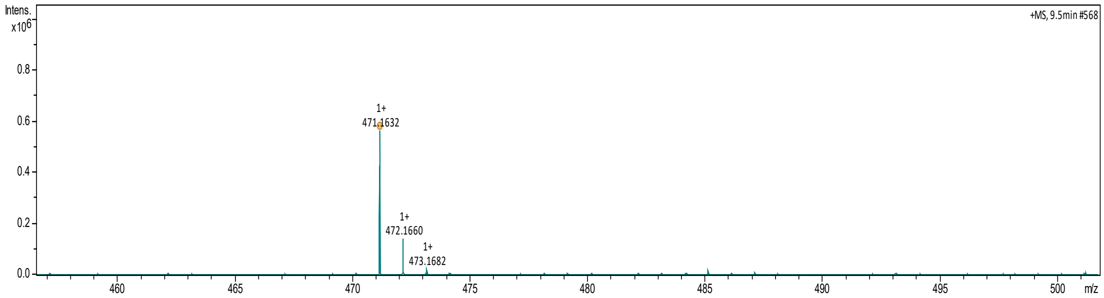

Meas. m/z	z	Ion Formula	m/z	err [mDa]	err [ppm]	mSigma	rdb	Adduct	Sum Formula
471.163162	1	$C_{23}H_{26}F_3O_7$	471.162514	-0.6	-1.4	3.0	9.5	M+H	$C_{23}H_{25}F_3O_7$

Figure S84. HRMS spectrum of (*R*)-MTPA Ester (**26b**)

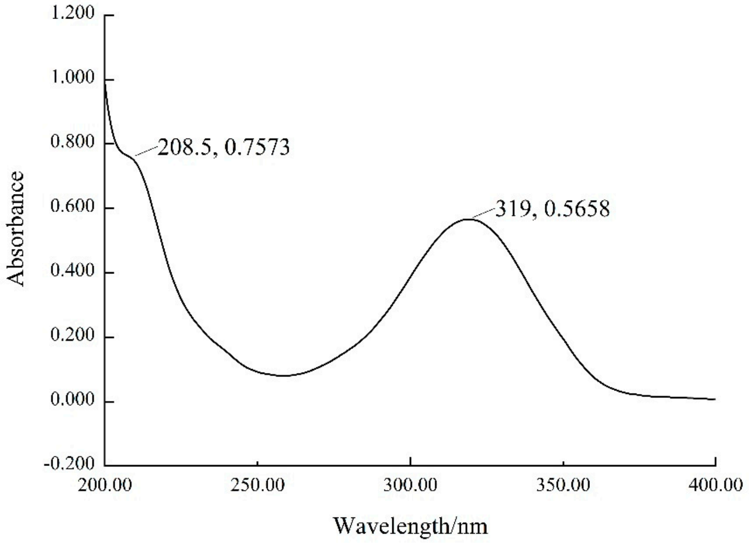

Figure S85. UV spectrum of nemanecin E (**26**)

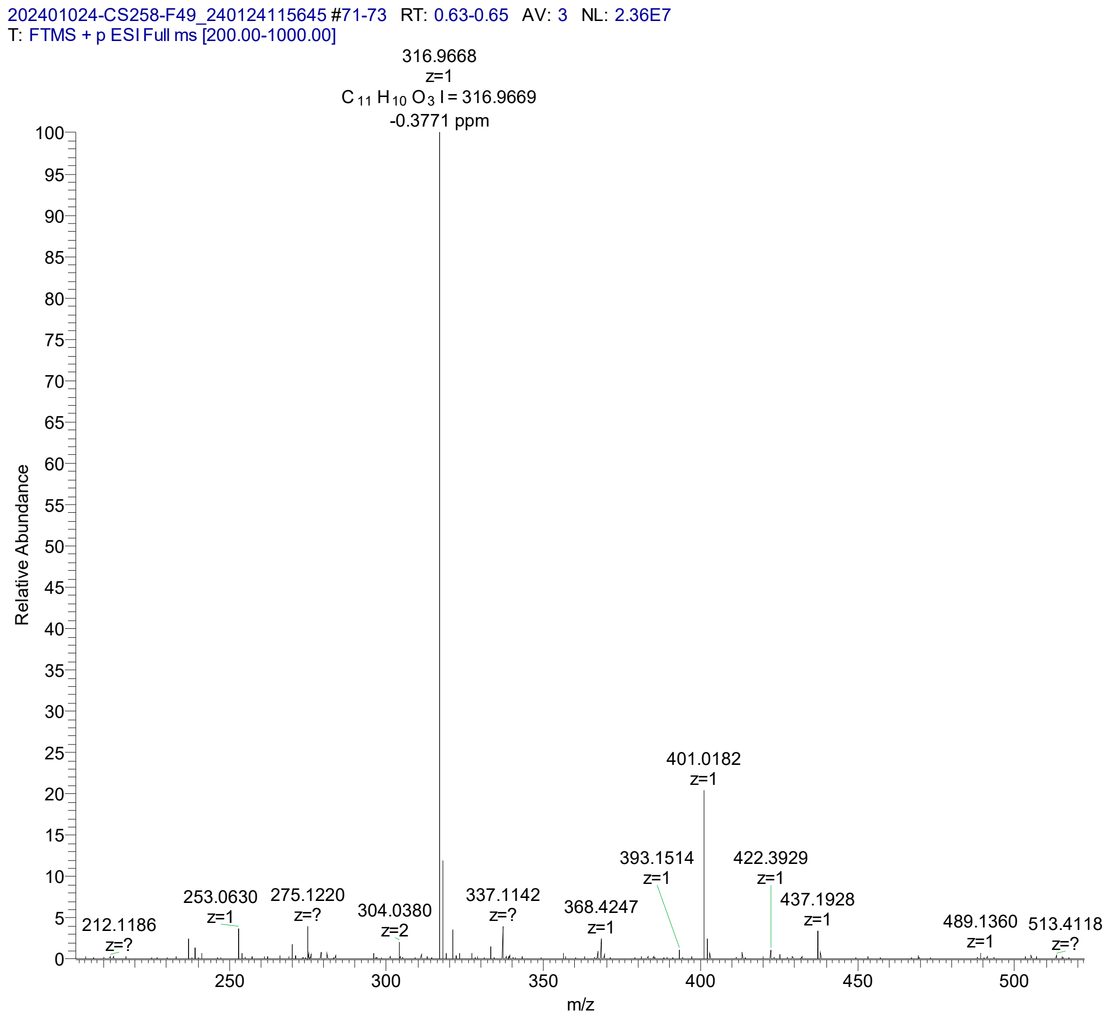

Figure S86. HRESIMS spectrum of 2,5-dimethy-8-iodochromone (**27**)

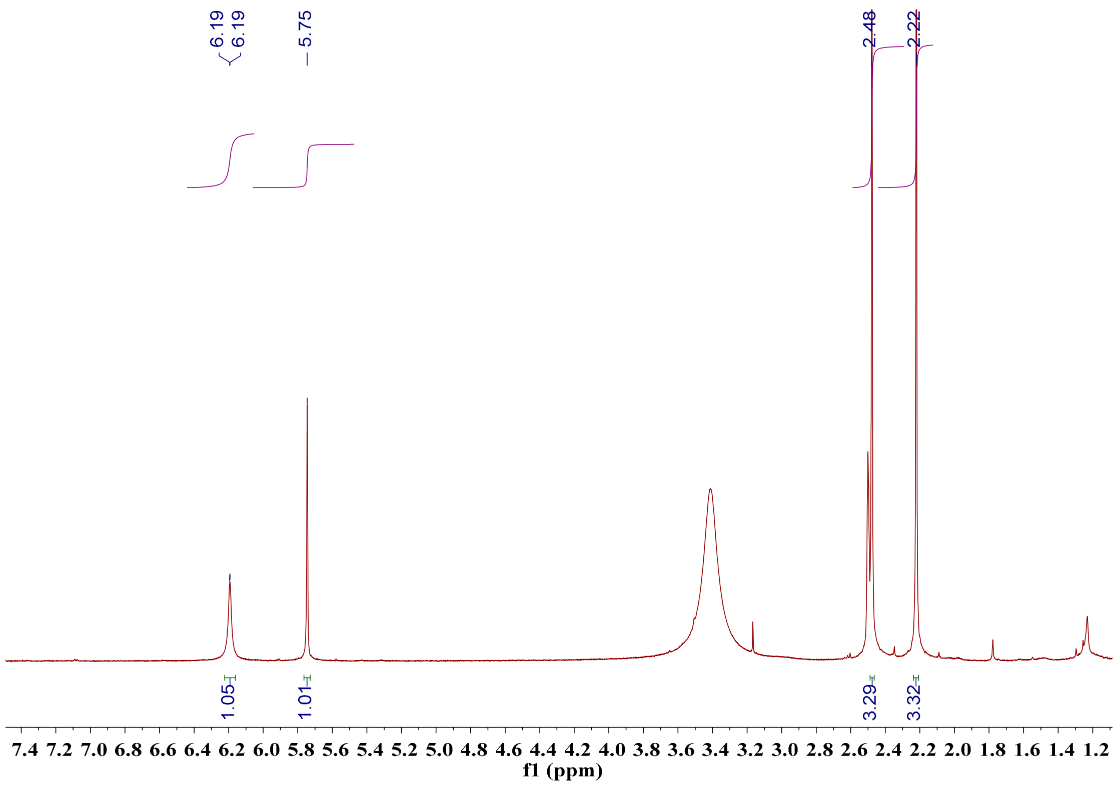

Figure S87. ^{1}H NMR spectrum (500 MHz, DMSO) of 2,5-dimethy-8-iodochromone (**27**)

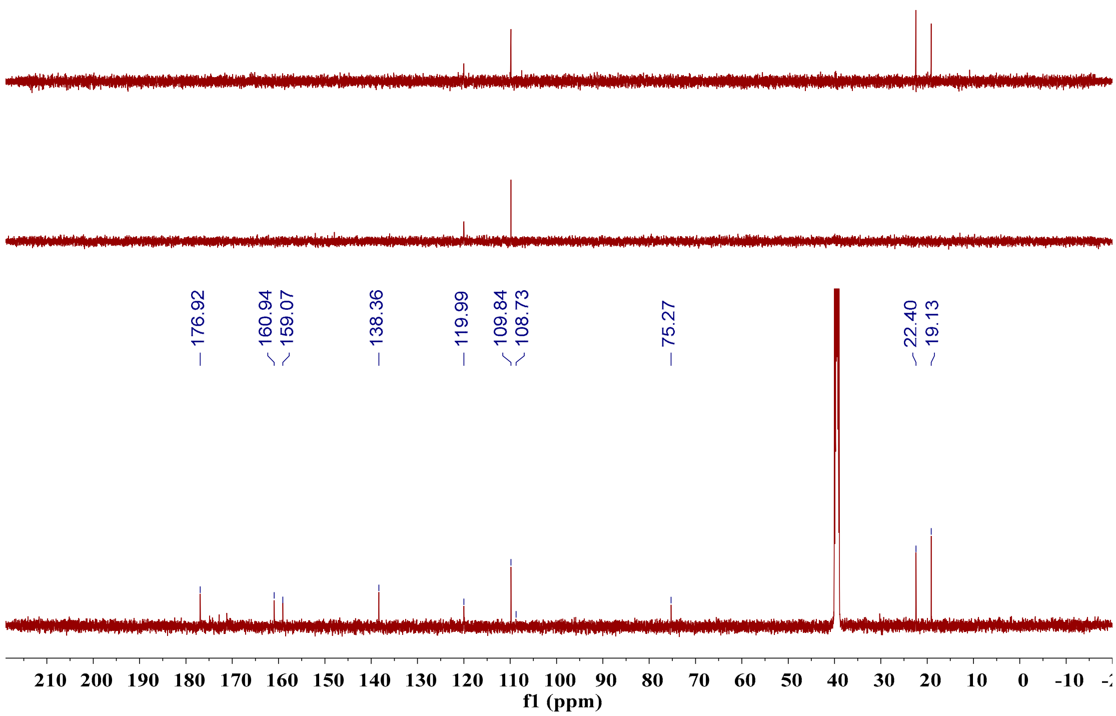

Figure S88. ^{13}C NMR spectrum (125 MHz, DMSO) of 2,5-dimethy-8-iodochromone (**27**)

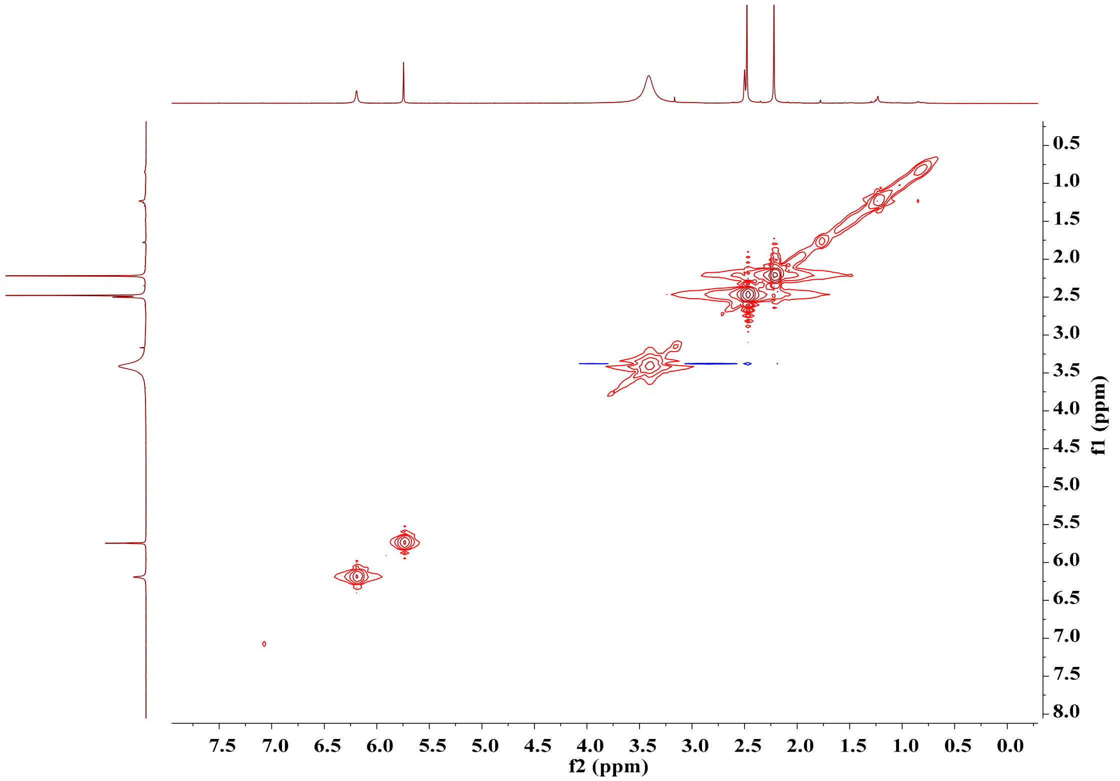

Figure S89. ^{1}H-^{1}H COSY spectrum of 2,5-dimethy-8-iodochromone (**27**)

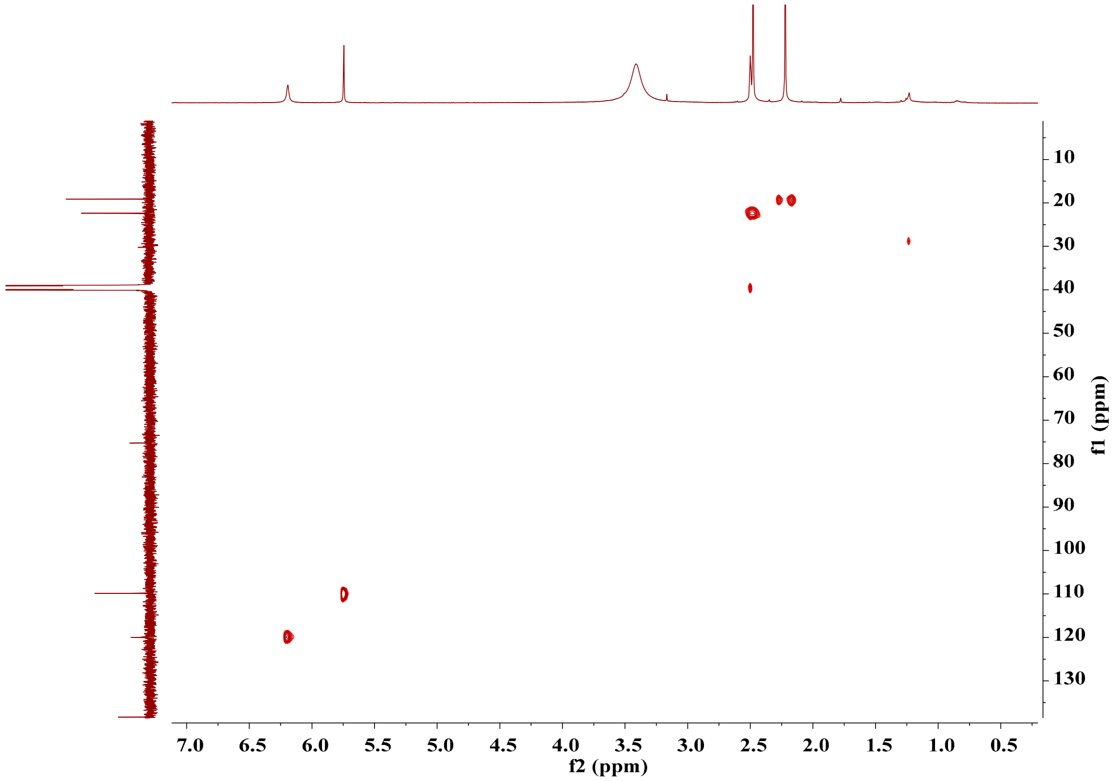

Figure S90. HSQC spectrum of 2,5-dimethy-8-iodochromone (**27**)

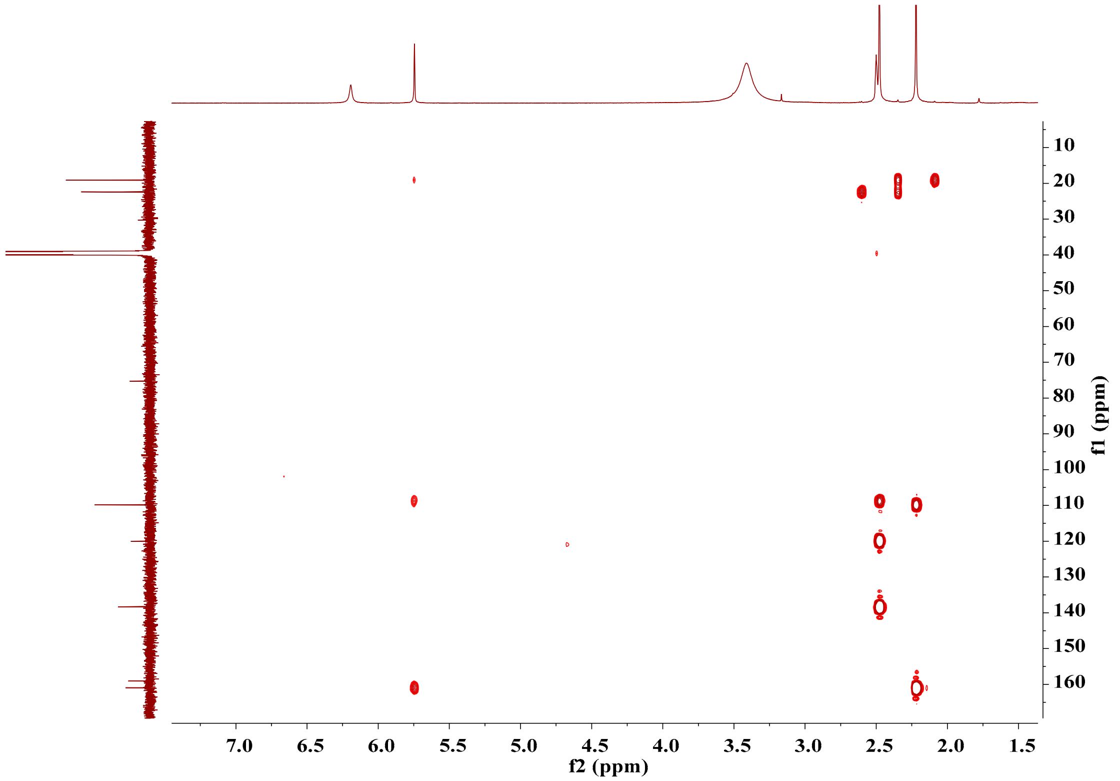

Figure S91. HMBC spectrum of 2,5-dimethy-8-iodochromone (**27**)

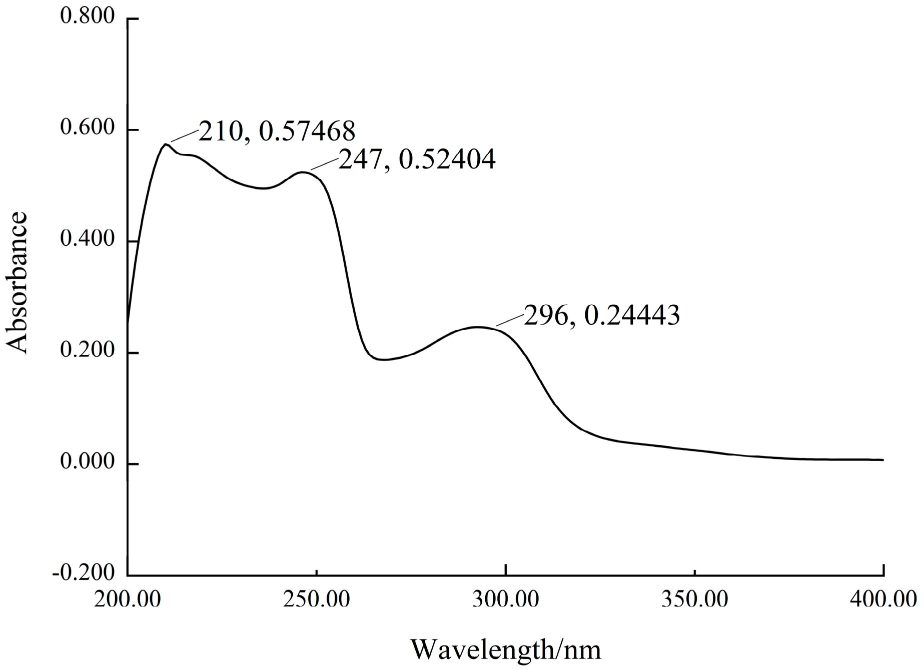

Figure S92. UV spectrum of 2,5-dimethy-8-iodochromone (**27**)

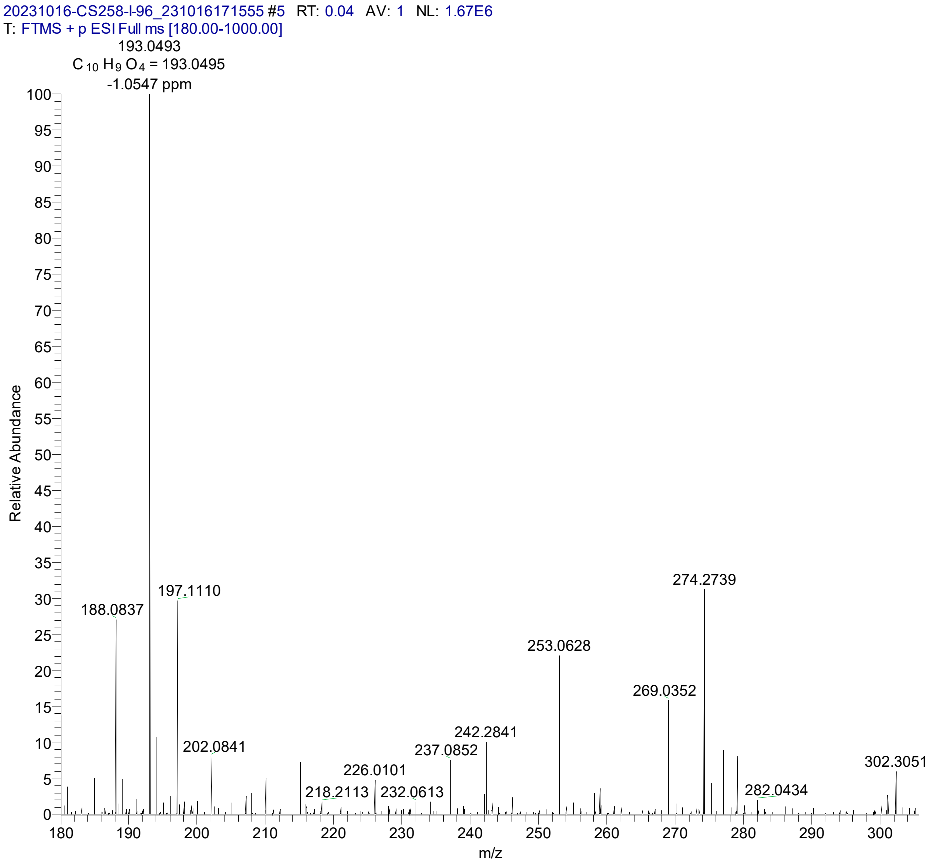

Figure S93. HRESIMS spectrum of 6-hydroxy-4-methoxycoumarin (**28**)

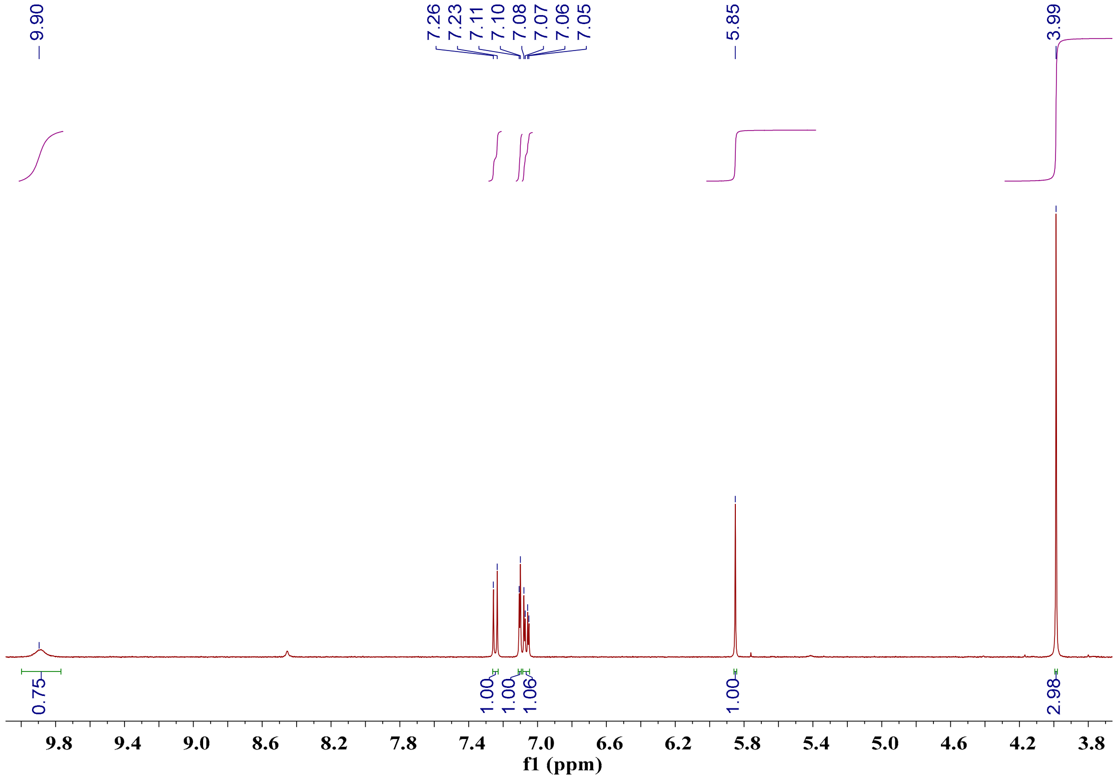

Figure S94. ^{1}H NMR spectrum (400 MHz, DMSO) of 6-hydroxy-4-methoxycoumarin (**28**)

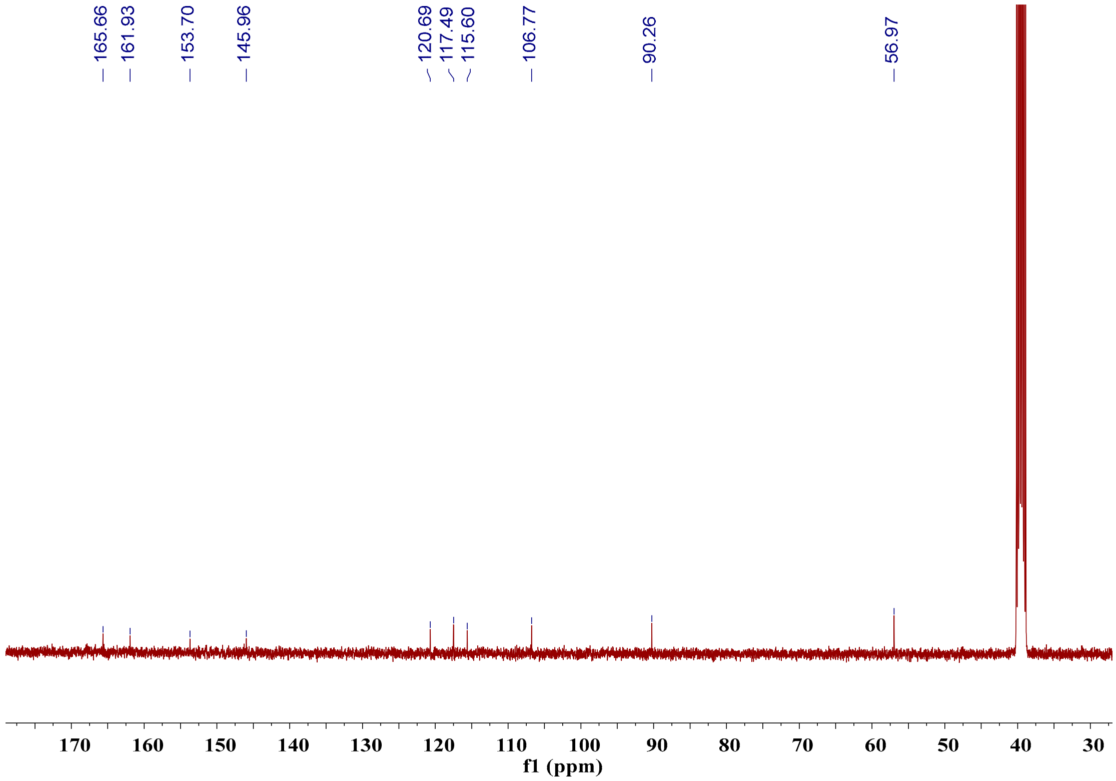

Figure S95. ^{13}C NMR spectrum (100 MHz, DMSO) of 6-hydroxy-4-methoxycoumarin (**28**)

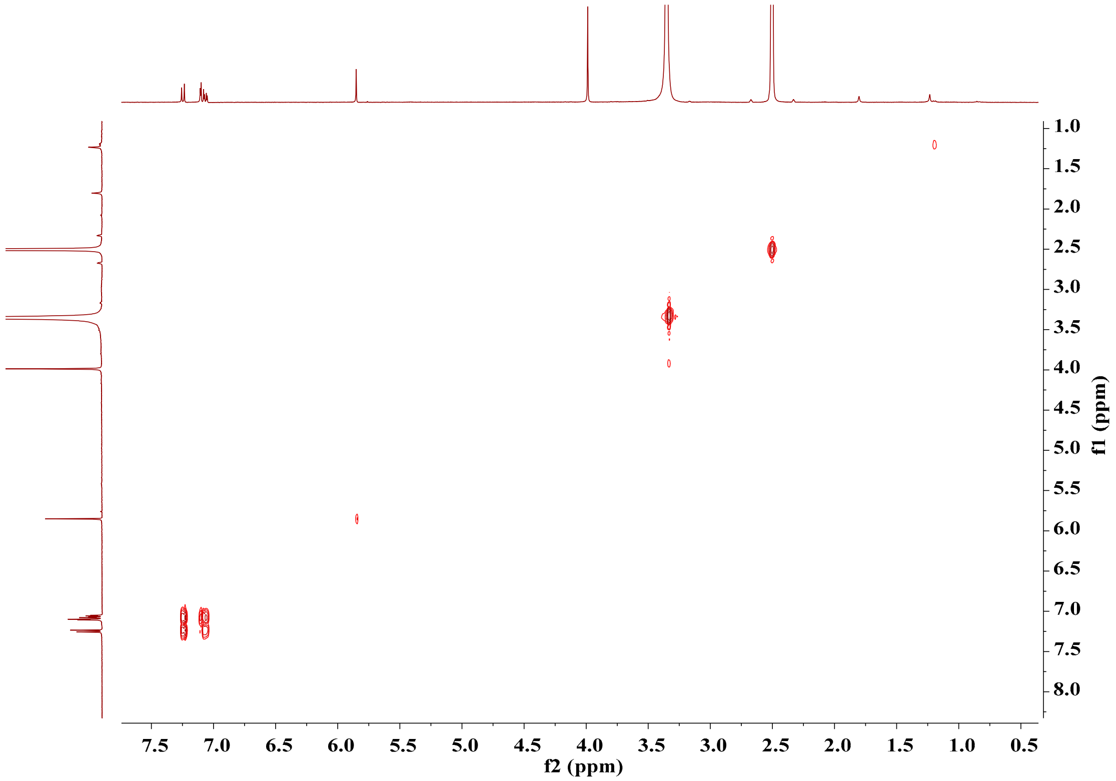

Figure S96. ^1H-^1H COSY spectrum of 6-hydroxy-4-methoxycoumarin (**28**)

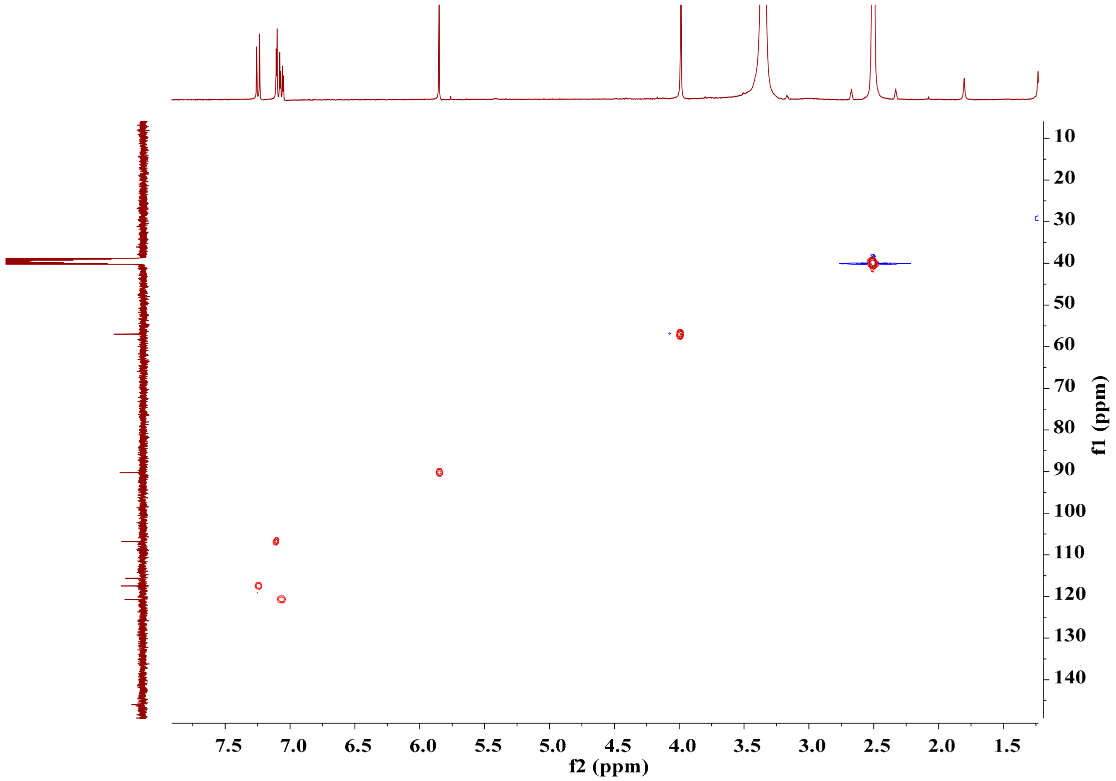

Figure S97. HSQC spectrum of 6-hydroxy-4-methoxycoumarin (**28**)

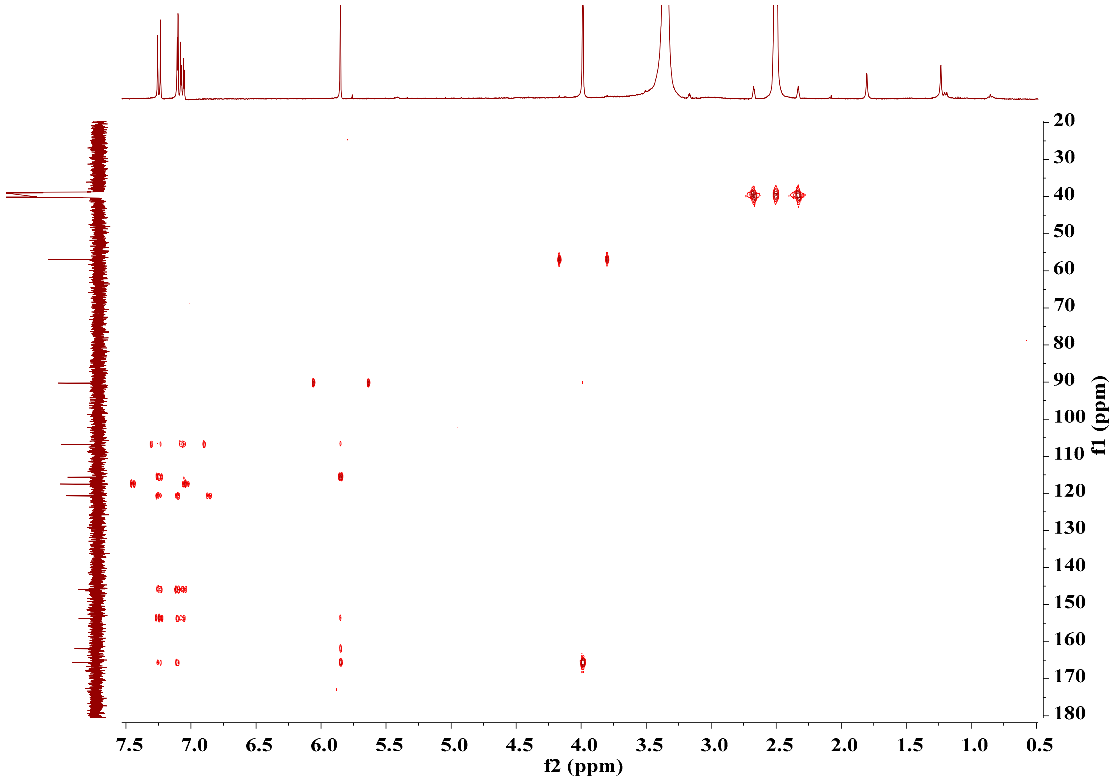

Figure S98. HMBC spectrum of 6-hydroxy-4-methoxycoumarin (**28**)

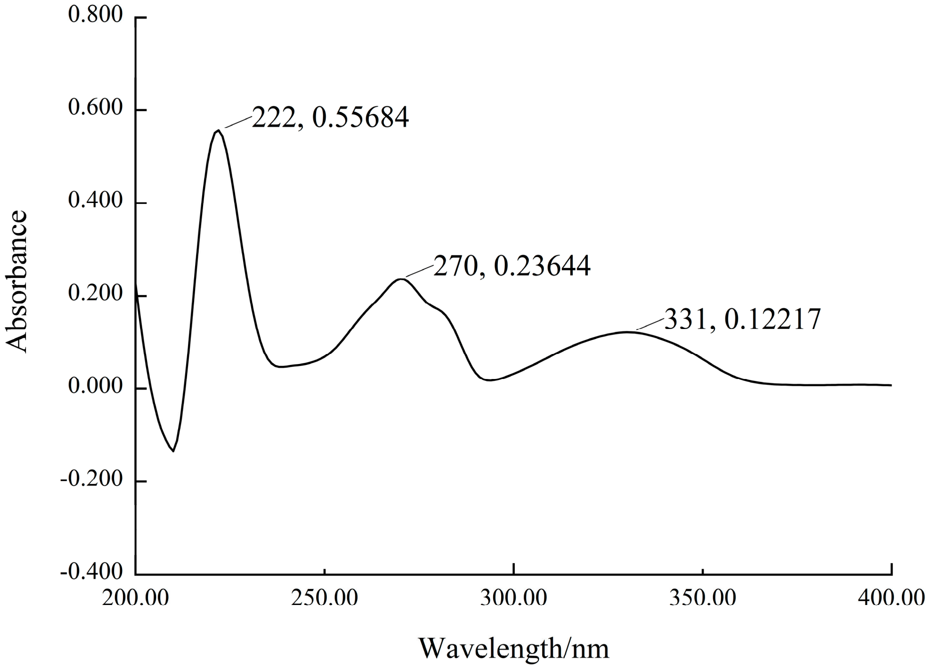

Figure S99. UV spectrum of 6-hydroxy-4-methoxycoumarin (**28**)

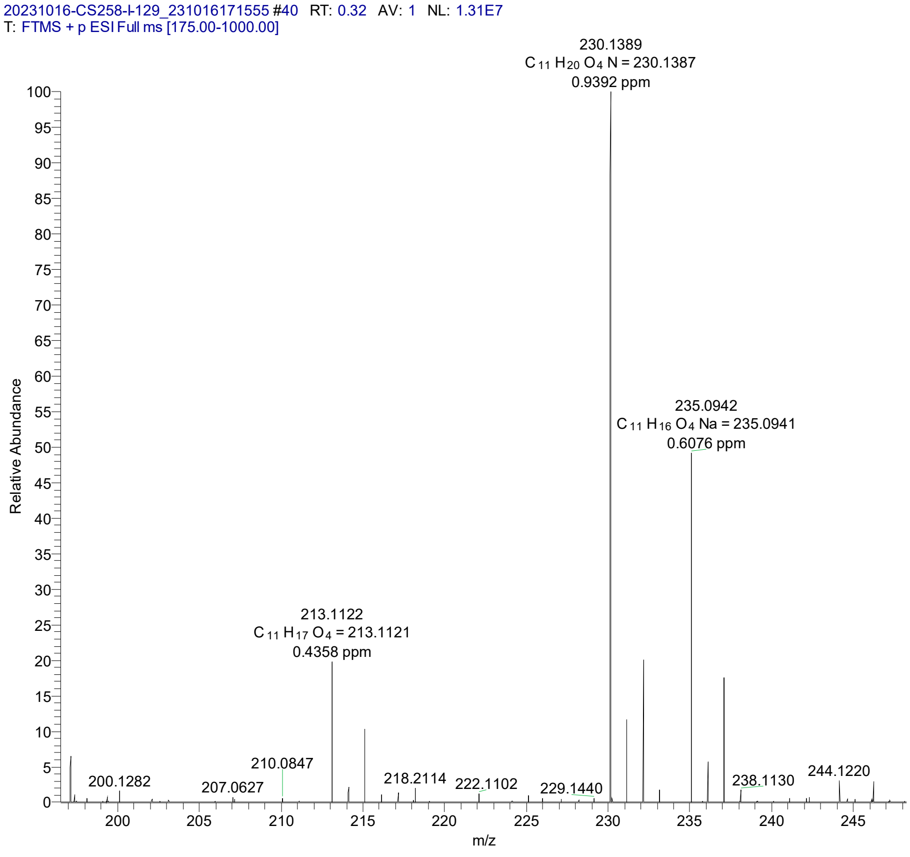

Figure S100. HRESIMS spectrum of talarofurolactone A (**29**)

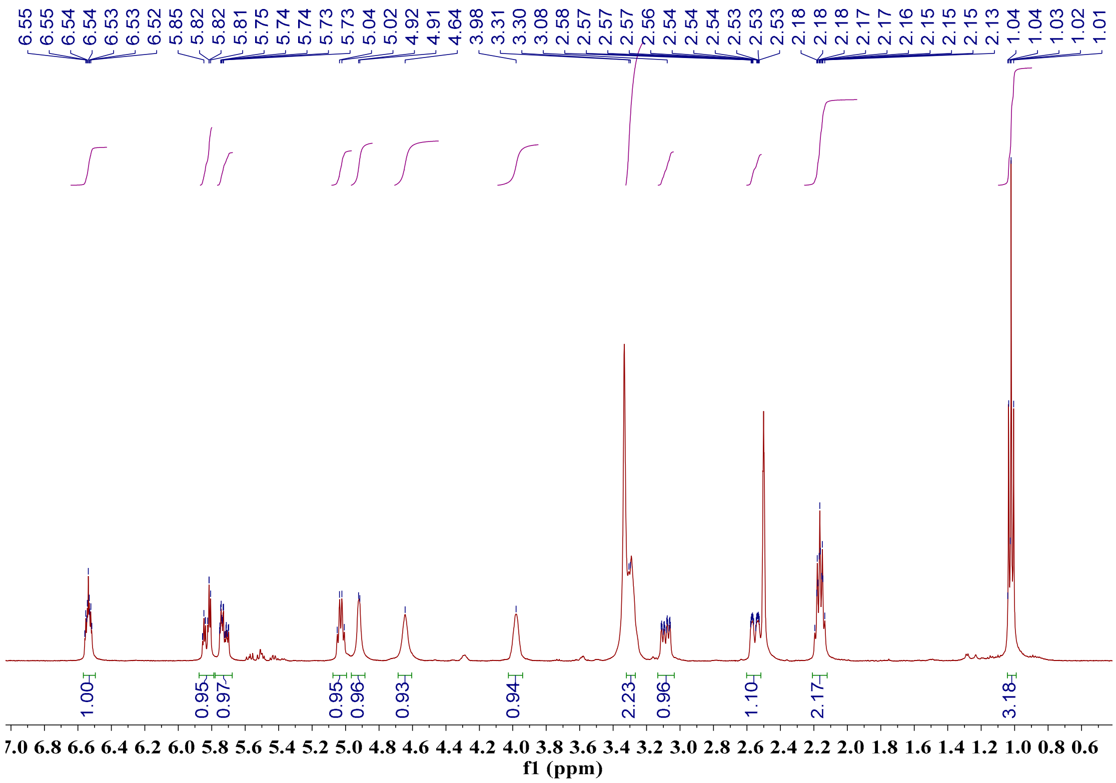

Figure S101. ^{1}H NMR spectrum (500 MHz, DMSO) of talarofurolactone A (**29**)

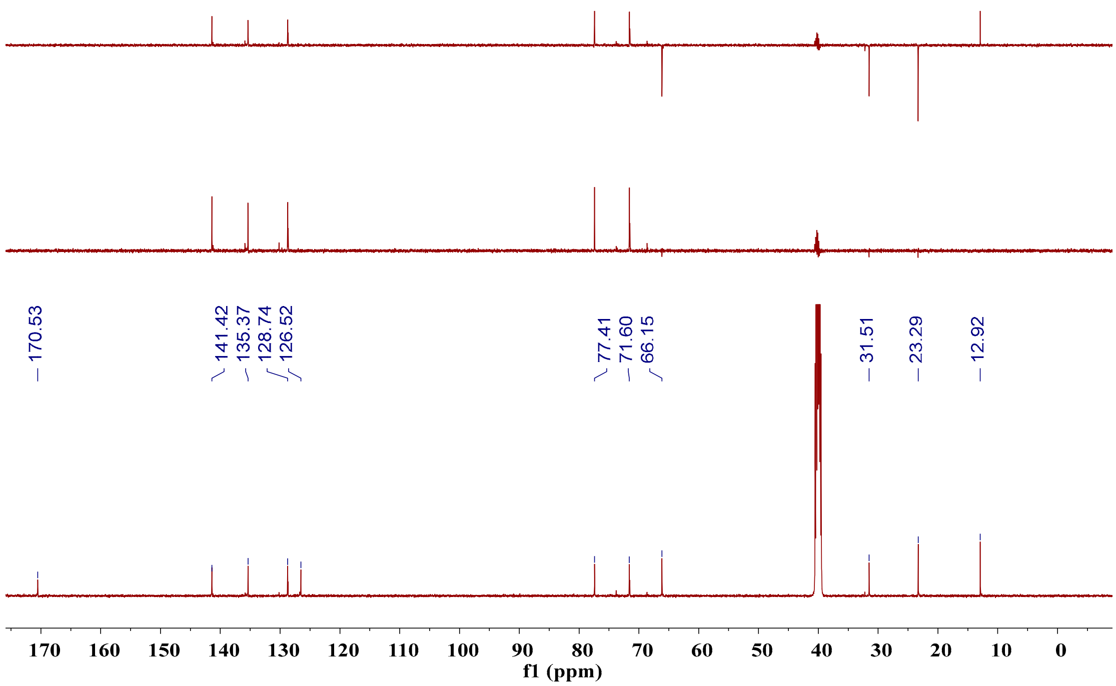

Figure S102. ^{13}C NMR spectrum (125 MHz, DMSO) of talarofurolactone A (**29**)

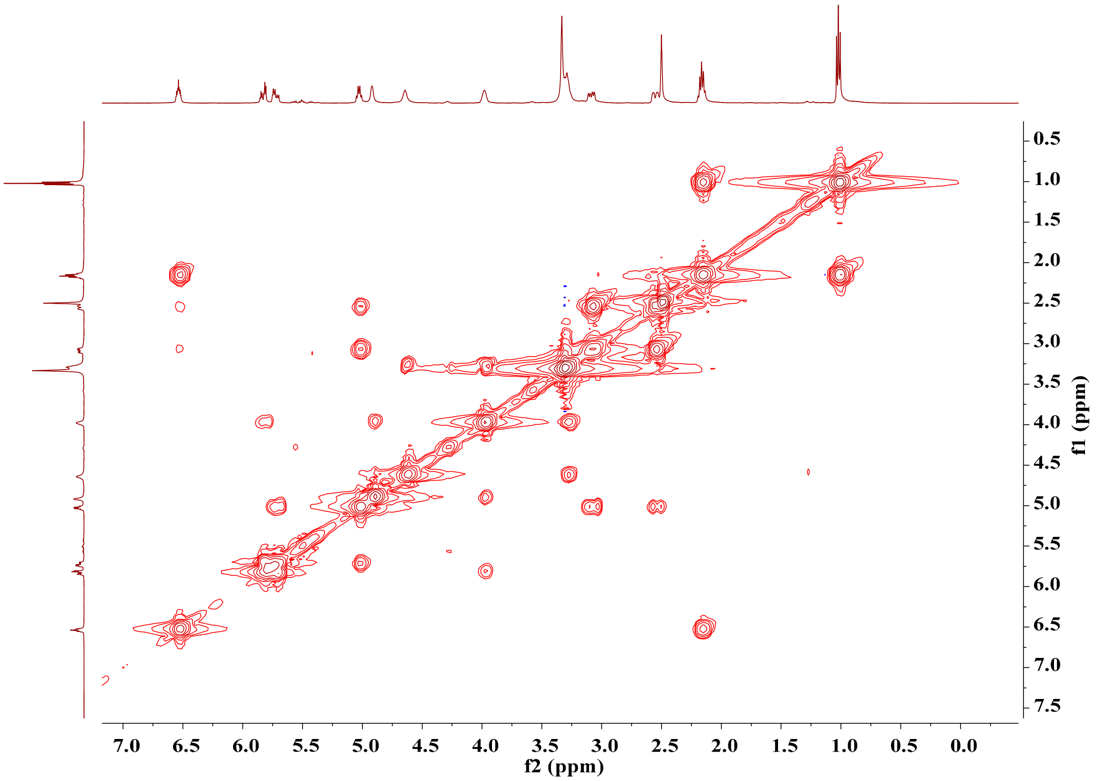

Figure S103. ^{1}H-^{1}H COSY spectrum of talarofurolactone A (**29**)

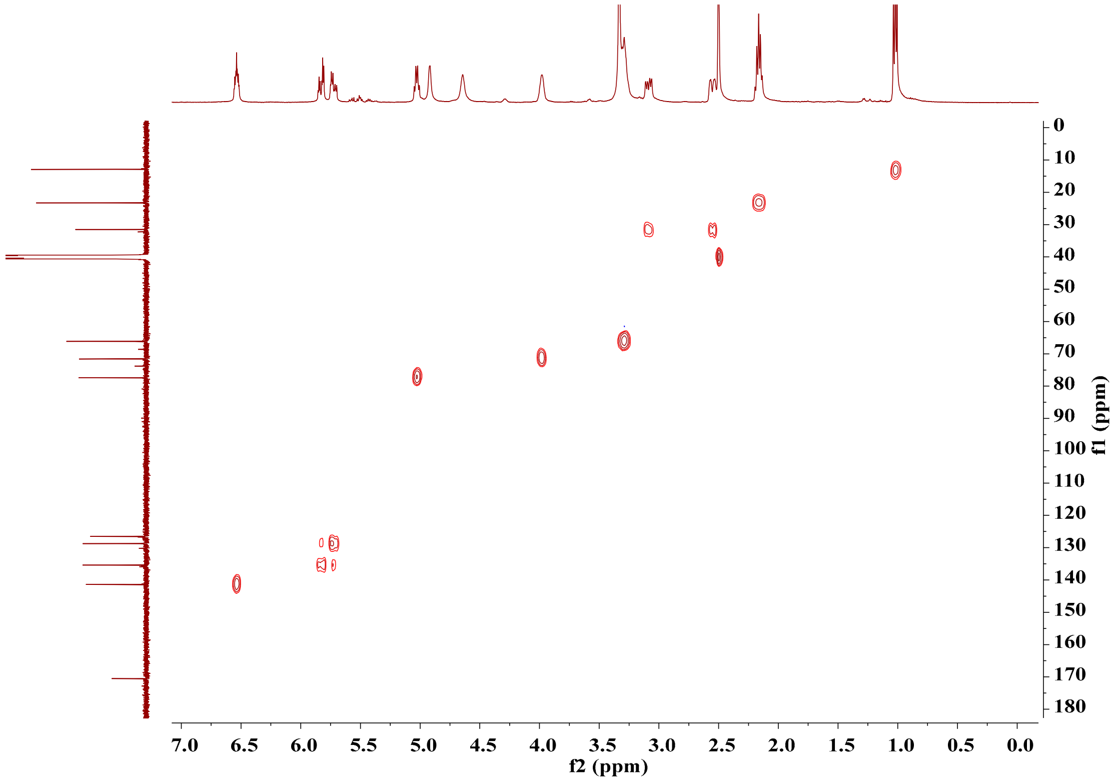

Figure S104. HSQC spectrum of talarofurolactone A (**29**)

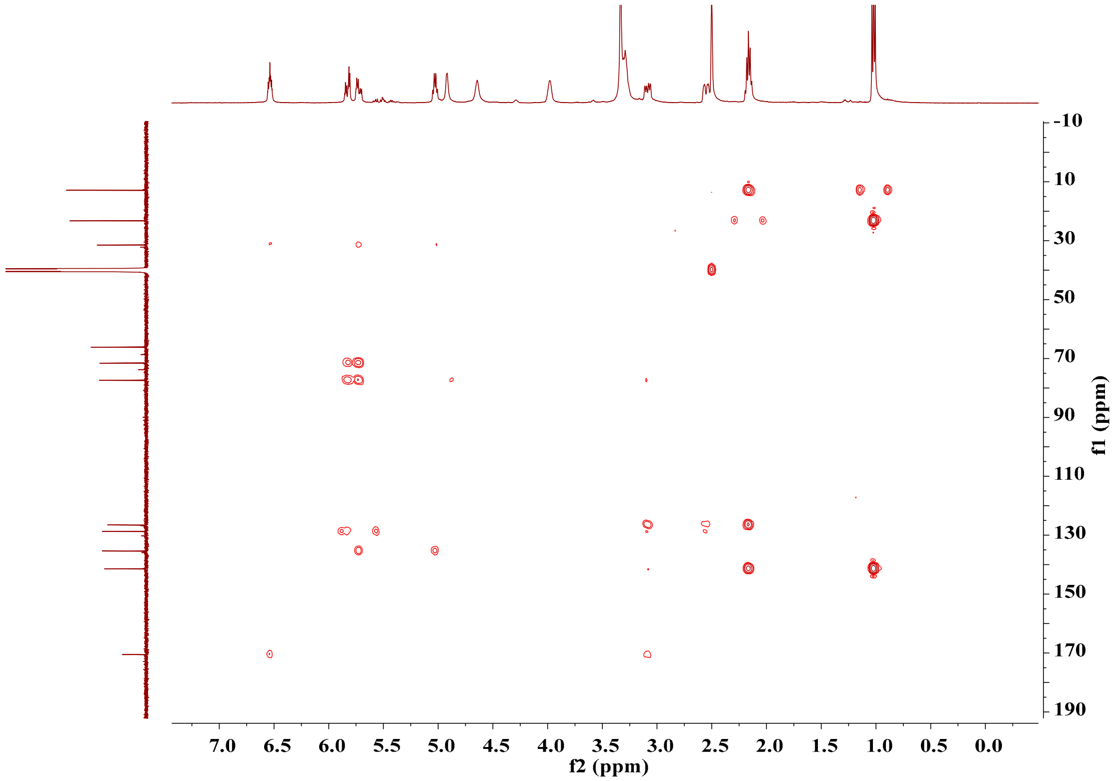

Figure S105. HMBC spectrum of talarofurolactone A (**20**)

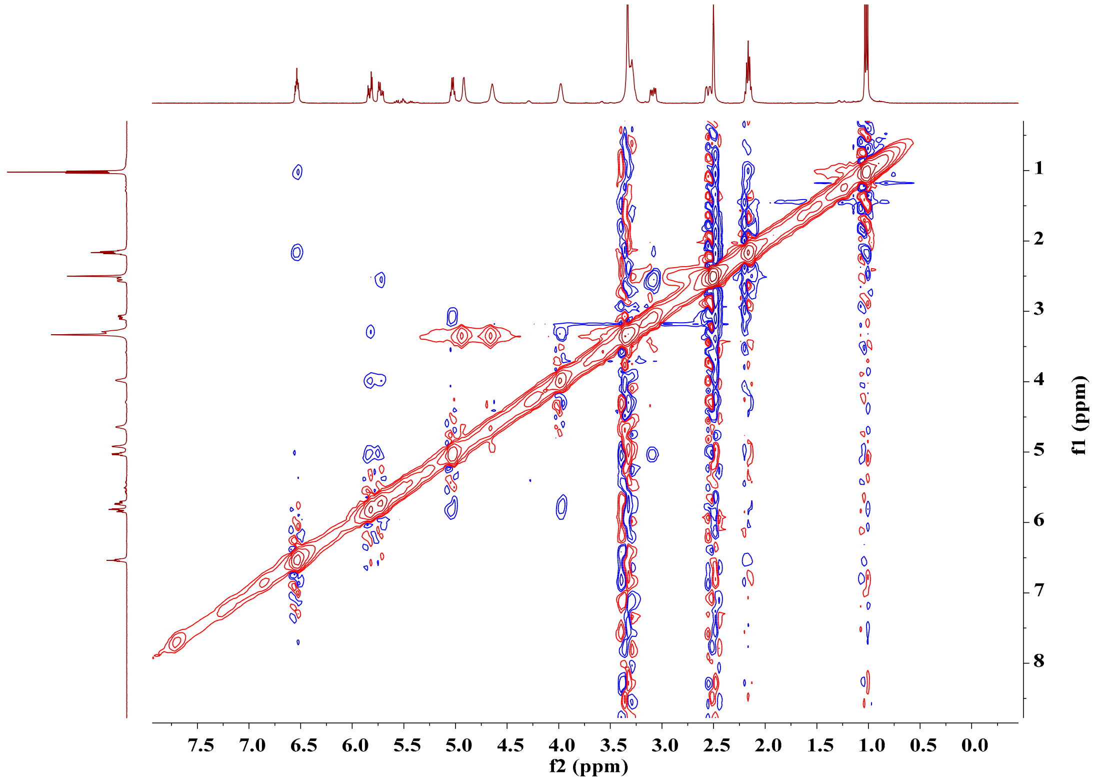

Figure S106. NOESY spectrum of talarofurolactone A (**29**)

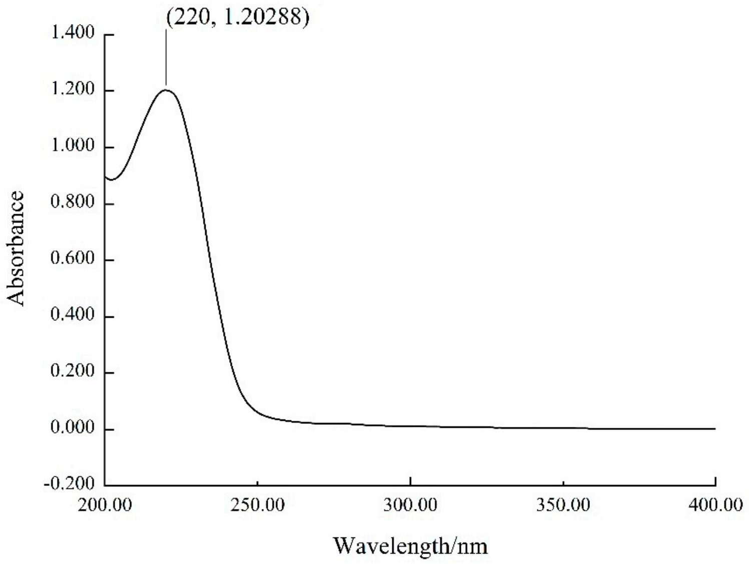

Figure S107. UV spectrum of talarofurolactone A (**29**)

Table S3. Energy analysis for the conformers of (3*R*,5'*R*)-5-hydroxytalaroflavone (**1**)

Compound	Conformations	G (Hartree)	G (Kcal/mol)	ΔG (Kcal/mol)	Boltzmann Dist (%)
	**1**-a	-952.6110	-597772.4335	0.1870	29.62%
**1**	**1**-b	-952.6113	-597772.6205	0.0000	40.63%
	**1**-c	-952.6110	-597772.4360	0.1845	29.75%

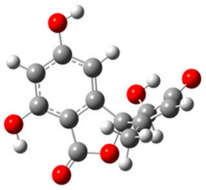

1-a

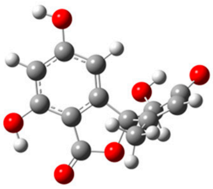

1-b

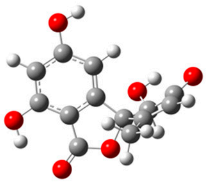

1-c

Figure S108. B3LYP/6-31G(d) optimized low-energy conformers of (3*R*,5'*R*)-5-hydroxytalaroflavone (**1**)

Table S4. Energy analysis for the conformers of (9a*S*)-talaroisochromenol A (**3**)

Compound	Conformations	G (Hartree)	G (Kcal/mol)	ΔG (Kcal/mol)	Boltzmann Dist (%)
	**3**-a	-952.5774	-597751.3366	5.4995	0.01%
**3**	**3**-b	-952.5861	-597756.8361	0.0000	56.32%
	**3**-c	-952.5859	-597756.6855	0.1506	43.67%

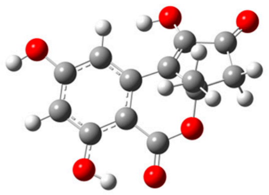

3-a

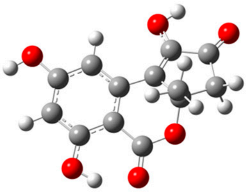

3-b

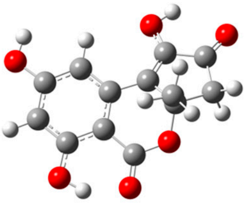

3-c

Figure S109. B3LYP/6-31G(d) optimized low-energy conformers of (9a*S*)-talaroisochromenol A (**3**)

Table S5**.** Energy analysis for the conformers of (7*R*,8*S*,9*R*)-talaroisochromenol B (**5**)

Compound	Conformations	G (Hartree)	G (Kcal/mol)	ΔG (Kcal/mol)	Boltzmann Dist (%)
	**5**-a	-953.7940	-598514.7841	0.0000	43.57%
	**5**-b	-953.7930	-598514.1610	0.6231	15.21%
	**5**-c	-953.7922	-598513.6697	1.1145	6.63%
	**5**-d	-953.7932	-598514.3242	0.4600	20.04%
**5**	**5**-e	-953.7921	-598513.6151	1.1691	6.05%
	**5**-f	-953.7910	-598512.9254	1.8587	1.89%
	**5**-g	-953.7914	-598513.1658	1.6183	2.83%
	**5**-h	-953.7914	-598513.1953	1.5889	2.98%
	**5**-i	-953.7902	-598512.4197	2.3645	0.80%

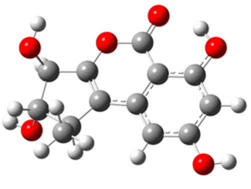

5-a

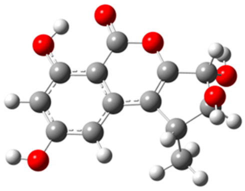

5-b

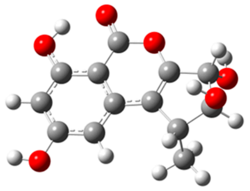

5-c

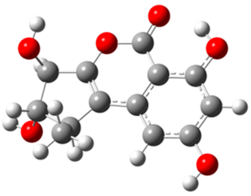

5-d

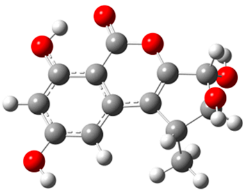

5-e

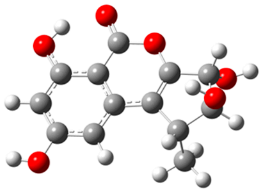

5-f

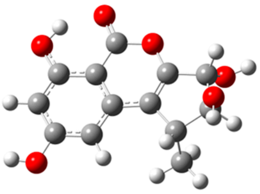

5-g

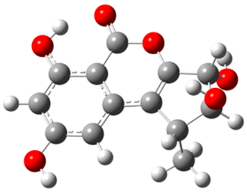

5-h

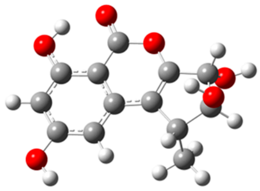

5-i

Figure S110. B3LYP/6-31G(d) optimized low-energy conformers of (7*R*,8*S*,9*R*)-talaroisochromenol B (**5**)

Table S6**.** Energy analysis for the conformers of (3a*S*, 9b*S*)-talaroisochromenol C (**11**)

Compound	Conformations	G (Hartree)	G (Kcal/mol)	ΔG (Kcal/mol)	Boltzmann Dist (%)
**11**	**11**-a	-914.5003	-573857.6549	1.5198	4.79%
	**11**-b	-914.5028	-573859.1747	0.0000	62.40%
	**11**-c	-914.5019	-573858.6137	0.5610	24.19%
	**11**-d	-914.5003	-573857.6555	1.5192	4.80%
	**11**-e	-914.5001	-573857.5200	1.6547	3.81%

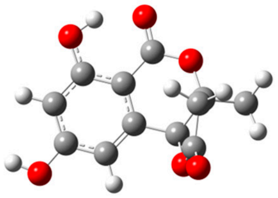

11-a

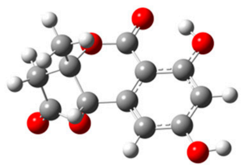

11-b

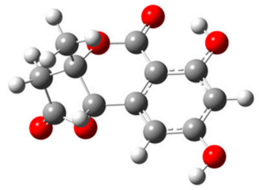

11-c

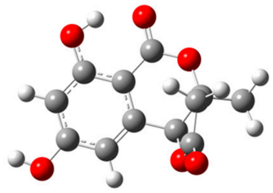

11-d

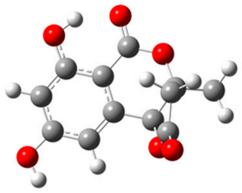

11-e

Figure S111. B3LYP/6-31G(d) optimized low-energy conformers of (3a*S*, 9b*S*)-talaroisochromenol C (**11**)

Table S7**.** Energy analysis for the conformers of (8*R*,9*R*,10a*R*)-5-hydroxyaltenuene (**13**)

Compound	Conformations	G (Hartree)	G (Kcal/mol)	ΔG (Kcal/mol)	Boltzmann Dist (%)
**13**	**13**-a	-993.0736	-623163.0944	1.8185	2.02%
	**13**-b	-993.0765	-623164.9129	0.0000	43.54%
	**13**-c	-993.0736	-623163.0944	1.8185	2.02%
	**13**-d	-993.0757	-623164.4322	0.4807	19.33%
	**13**-e	-993.0756	-623164.3902	0.5227	18.01%
	**13**-f	-993.0753	-623164.1724	0.7405	12.47%
	**13**-g	-993.0724	-623162.3639	2.5489	0.59%
	**13**-h	-993.0736	-623163.0962	1.8166	2.02%

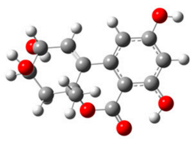 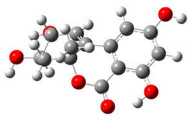 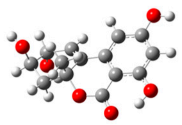 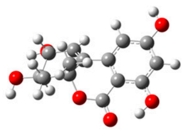

13-a **13-b** **13-c** **13-d**

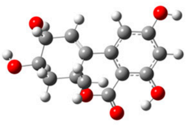 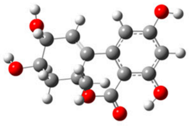 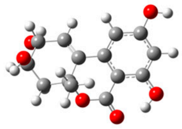 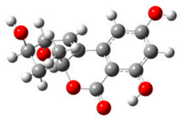

13-e **13-f** **13-g** **13-h**

Figure S112. B3LYP/6-31G(d) optimized low-energy conformers of (8*R*,9*R*,10a*R*)-5-hydroxyaltenuene (**13**)

Table S8**.** Energy analysis for the conformers of (8*S*,9*S*,10a*R*)-5-hydroxyaltenuene (*ent*-**14**)

Compound	Conformations	G (Hartree)	G (Kcal/mol)	ΔG (Kcal/mol)	Boltzmann Dist (%)
**14**	**14**-a	-993.0764	-623164.8984	0.3025	13.16%
	**14**-b	-993.0769	-623165.2009	0.0000	21.94%
	**14**-c	-993.0764	-623164.8972	0.3037	13.13%
	**14**-d	-993.0760	-623164.6380	0.5629	8.48%
	**14**-e	-993.0763	-623164.8012	0.3997	11.17%
	**14**-f	-993.0765	-623164.9631	0.2378	14.68%
	**14**-g	-993.0764	-623164.8997	0.3012	13.19%
	**14**-h	-993.0754	-623164.2289	0.9720	4.25%

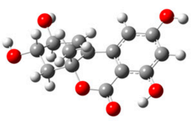 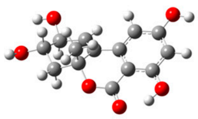 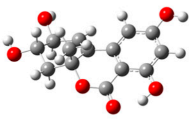 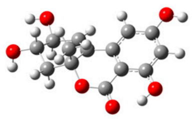

14-a **14-b** **14-c** **14-d**

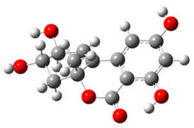 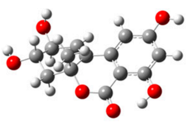 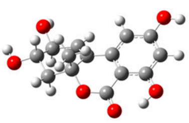 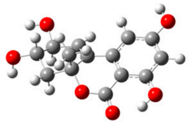

14-e **14-f** **14-g** **14-h**

Figure S113. B3LYP/6-31G(d) optimized low-energy conformers of (8*S*,9*S*,10a*R*)-5-hydroxyaltenuene (*ent*-**14**)

Table S9**.** Energy analysis for the conformers of (8*R*,9*S*,10a*R*)-5-hydroxyaltenuene (**15**)

Compound	Conformations	G (Hartree)	G (Kcal/mol)	ΔG (Kcal/mol)	Boltzmann Dist (%)
**15**	**15**-a	-993.0759	-623164.5746	0.5466	13.78%
	**15**-b	-993.0756	-623164.3782	0.7430	9.89%
	**15**-c	-993.0759	-623164.5684	0.5528	13.64%
	**15**-d	-993.0768	-623165.1212	0.0000	34.69%
	**15**-e	-993.0762	-623164.7403	0.3809	18.23%
	**15**-f	-993.0745	-623163.6698	1.4514	2.99%
	**15**-g	-993.0747	-623163.8122	1.3090	3.80%
	**15**-h	-993.0745	-623163.6660	1.4552	2.97%

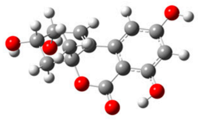
15-a
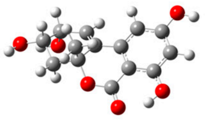
15-b
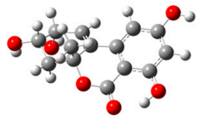
15-c
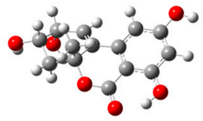
15-d
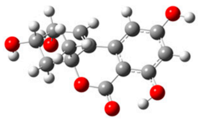
15-e
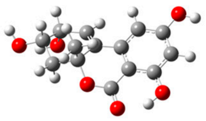
15-f
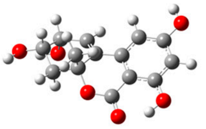
15-g
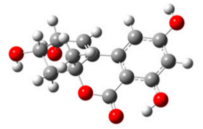
15-h

Figure S114. B3LYP/6-31G(d) optimized low-energy conformers of (8*R*,9*S*,10a*R*)-5-hydroxyaltenuene (**15**)

Table S10**.** Energy analysis for the conformers of (7*S*,8*R*,8a*R*,10*S*)-nemanecin D (**25a**)

Compound	Conformations	G (Hartree)	G (Kcal/mol)	ΔG (Kcal/mol)	Boltzmann Dist (%)
**25a**	**25a**-a	-882.0324	-553483.7003	12.1442	0.00%
	**25a**-b	-882.0517	-553495.8445	0.0000	34.80%
	**25a**-c	-882.0506	-553495.1379	0.7066	10.55%
	**25a**-d	-882.0514	-553495.6336	0.2108	24.37%
	**25a**-e	-882.0509	-553495.2935	0.5510	13.72%
	**25a**-f	-882.0497	-553494.5549	1.2895	3.94%
	**25a**-g	-882.0500	-553494.7432	1.1013	5.42%
	**25a**-h	-882.0497	-553494.5700	1.2745	4.04%
	**25a**-i	-882.0483	-553493.7047	2.1398	0.94%
	**25a**-j	-882.0491	-553494.2123	1.6322	2.21%

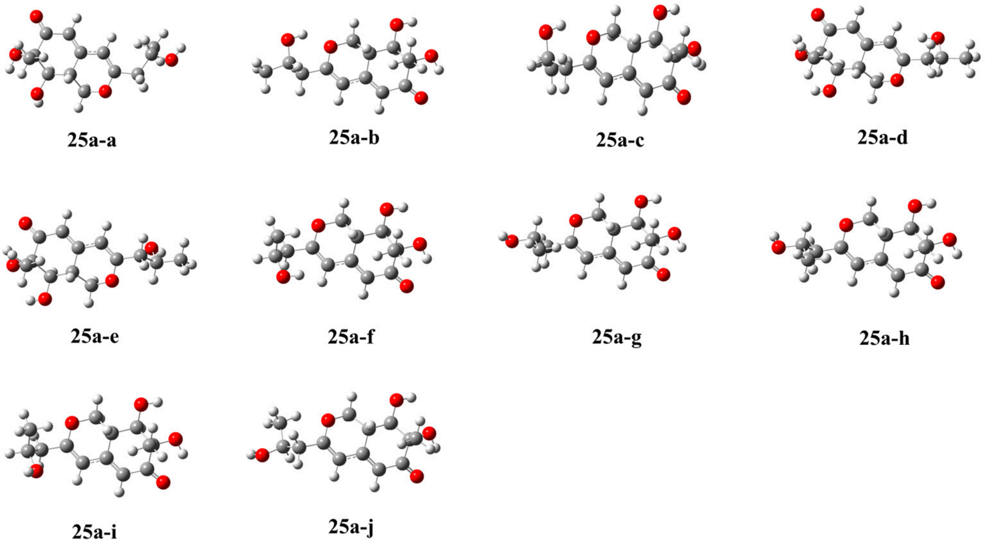

Figure S115. B3LYP/6-31G(d) optimized low-energy conformers of (7*S*,8*R*,8a*R*,10*S*)-nemanecin D (**25a**)

Table S11. Energy analysis for the conformers of (7*R*,8*S*,8a*S*,10*S*)-nemanecin D (**25b**)

Compound	Conformations	G (Hartree)	G (Kcal/mol)	ΔG (Kcal/mol)	Boltzmann Dist (%)
**25b**	**25b**-a	-882.0330	-553484.0818	11.8731	0.00%
	**25b**-b	-882.0519	-553495.9549	0.0000	41.61%
	**25b**-c	-882.0504	-553494.9929	0.9620	8.20%
	**25b**-d	-882.0515	-553495.6957	0.2592	26.86%
	**25b**-e	-882.0501	-553494.8430	1.1119	6.36%
	**25b**-f	-882.0496	-553494.4746	1.4803	3.42%
	**25b**-g	-882.0501	-553494.8103	1.1446	6.02%
	**25b**-h	-882.0498	-553494.6428	1.3121	4.54%
	**25b**-i	-882.0485	-553493.8094	2.1455	1.11%
	**25b**-j	-882.0490	-553494.1257	1.8292	1.89%

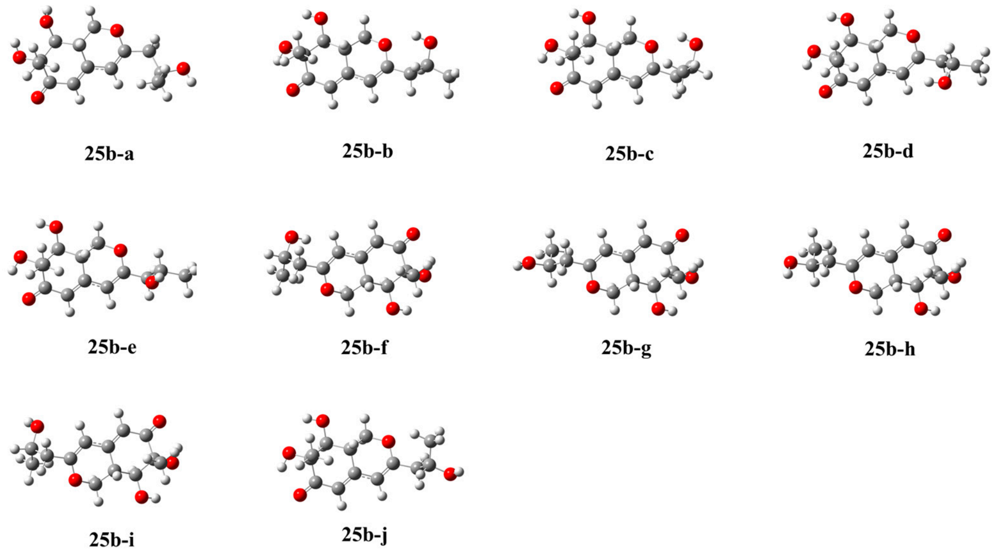

Figure S116. B3LYP/6-31G(d) optimized low-energy conformers of (7*R*,8*S*,8a*S*,10*S*)-nemanecin D (**25b**)

Table S12**.** Energy analysis for the conformers of (7*R*,8*S*,8a*S*,10*R*)-nemanecin E (**26a**)

Compound	Conformations	G (Hartree)	G (Kcal/mol)	ΔG (Kcal/mol)	Boltzmann Dist (%)
**26a**	**26a-**a	-882.0363	-553486.1287	9.7157	0.00%
	**26a-**b	-882.0517	-553495.8445	0.0000	34.80%
	**26a-**c	-882.0506	-553495.1379	0.7066	10.55%
	**26a-**d	-882.0514	-553495.6336	0.2108	24.37%
	**26a-**e	-882.0509	-553495.2935	0.5510	13.72%
	**26a-**f	-882.0497	-553494.5549	1.2895	3.94%
	**26a-**g	-882.0500	-553494.7432	1.1013	5.42%
	**26a-**h	-882.0497	-553494.5700	1.2745	4.04%
	**26a-**i	-882.0483	-553493.7040	2.1404	0.94%
	**26a-**j	-882.0491	-553494.2123	1.6322	2.21%

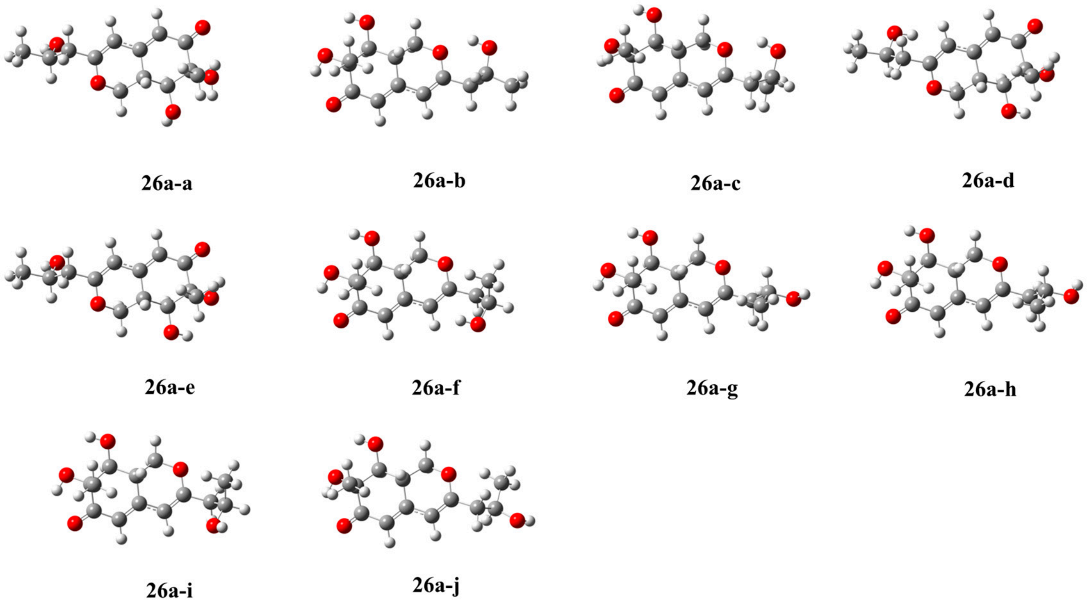

Figure S117. B3LYP/6-31G(d) optimized low-energy conformers of (7*R*,8*S*,8a*S*,10*R*)-nemanecin E (**26a**)

Table S13**.** Energy analysis for conformers of (7*S*,8*R*,8a*R*,10*R*)-nemanecin E (**26b**)

Compound	Conformations	G (Hartree)	G (Kcal/mol)	ΔG (Kcal/mol)	Boltzmann Dist (%)
**26b**	**26b-**a	-882.0330	-553484.0818	11.8731	0.00%
	**26b-**b	-882.0519	-553495.9549	0.0000	41.61%
	**26b-**c	-882.0504	-553494.9929	0.9620	8.20%
	**26b-**d	-882.0515	-553495.6957	0.2592	26.86%
	**26b-**e	-882.0501	-553494.8430	1.1119	6.36%
	**26b-**f	-882.0496	-553494.4746	1.4803	3.42%
	**26b-**g	-882.0501	-553494.8103	1.1446	6.02%
	**26b-**h	-882.0498	-553494.6428	1.3121	4.54%
	**26b-**i	-882.0485	-553493.8094	2.1455	1.11%
	**26b-**j	-882.0490	-553494.1257	1.8292	1.89%

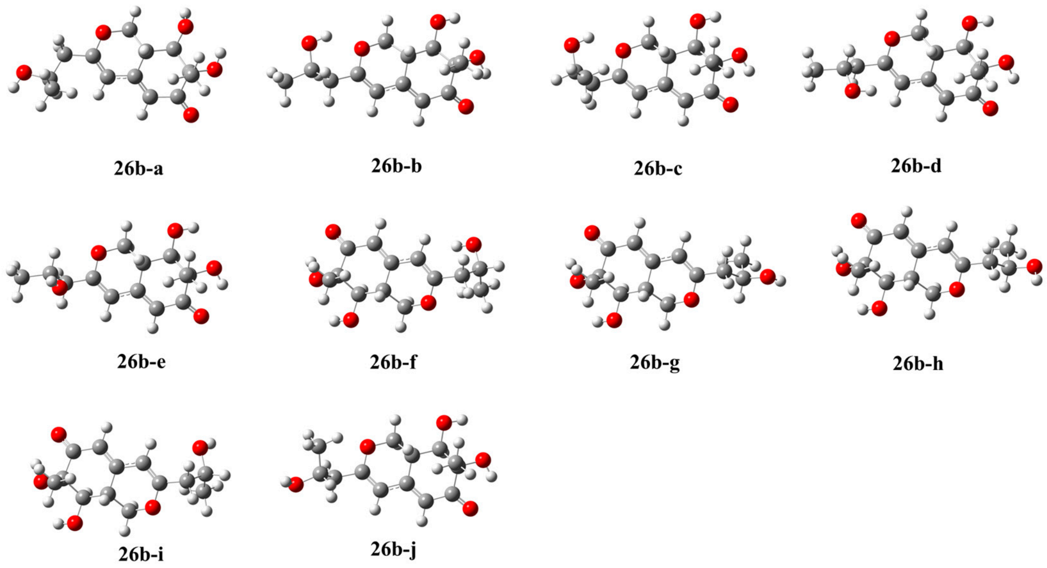

Figure S118. B3LYP/6-31G(d) optimized low-energy conformers of (7*S*,8*R*,8a*R*,10*R*)-nemanecin E (**26b**)

Table S14**.** Energy analysis for the conformers of (5*R*,3'*S*)-talarofurolactone A (**29a**)

compound	Conformations	G (Hartree)	G (Kcal/mol)	ΔG (Kcal/mol)	Boltzmann Dist (%)
**29a**	**29a**-a	-729.4334	-457726.3975	4.1585	0.02%
	**29a**-b	-729.4391	-457729.9448	0.6112	9.47%
	**29a**-c	-729.4370	-457728.6415	1.9145	1.05%
	**29a**-d	-729.4399	-457730.4701	0.0860	23.00%
	**29a**-e	-729.4394	-457730.1243	0.4317	12.83%
	**29a**-f	-729.4380	-457729.2847	1.2713	3.11%
	**29a**-g	-729.4388	-457729.7585	0.7976	6.91%
	**29a**-h	-729.4400	-457730.5560	0.0000	26.59%
	**29a**-i	-729.4373	-457728.8480	1.7081	1.49%
	**29a**-j	-729.4395	-457730.2379	0.3181	15.54%

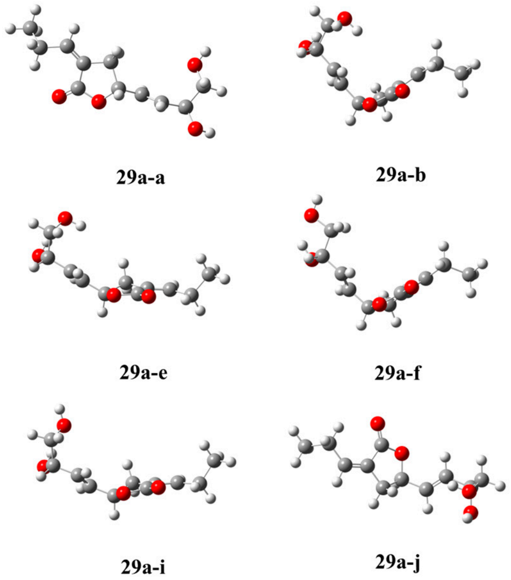

Figure S119. B3LYP/6-31G(d) optimized low-energy conformers of (5*R*,3'*S*)-talarofurolactone A (**29a**)

Table S15**.** Energy analysis for the conformers of (5*S*,3'*R*)-talarofurolactone A (**29b**)

Compound	Conformations	G (Hartree)	G (Kcal/mol)	ΔG (Kcal/mol)	Boltzmann Dist (%)
**29b**	**29b**-a	-729.4380	-457729.2935	1.2625	3.06%
	**29b**-b	-729.4391	-457729.9448	0.6112	9.20%
	**29b**-c	-729.4399	-457730.4701	0.0860	22.34%
	**29b**-d	-729.4394	-457730.1243	0.4317	12.46%
	**29b**-e	-729.4380	-457729.2847	1.2713	3.02%
	**29b**-f	-729.4388	-457729.7585	0.7976	6.72%
	**29b**-g	-729.4400	-457730.5560	0.0000	25.83%
	**29b**-h	-729.4373	-457728.8480	1.7081	1.44%
	**29b**-i	-729.4395	-457730.2379	0.3181	15.09%
	**29b**-j	-729.4368	-457728.5173	2.0388	0.83%

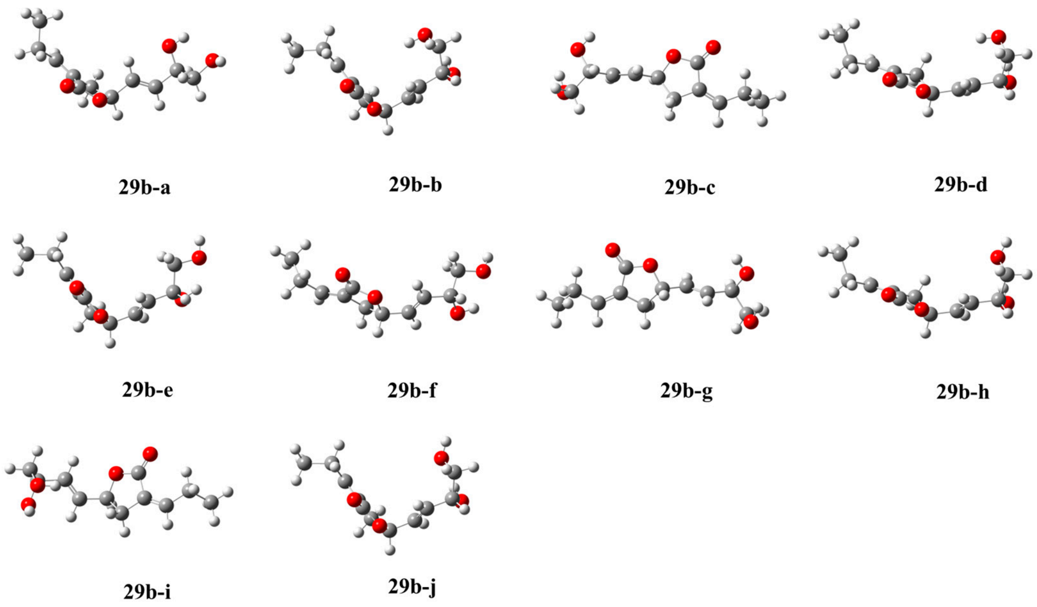

Figure S120. B3LYP/6-31G(d) optimized low-energy conformers of (5*S*,3'*R*)-talarofurolactone A (**29b**)

Table S16**.** DP4^{+} probability analysis of **15** (mPW1PW91/6-31+G (d, p) level).

mPW1PW91		PCM		6-31+G(d, p)		Shielding Tensors	
		DP4+	100. 00%	0. 00%	–	–	–
Nuclei	sp2?	xperimenta	Isomer 1	Isomer 2	Isomer 3	Isomer 4	Isomer 5
C		25. 8	170. 784105	170. 262018			
C		38. 4	155. 791038	152. 97446			
C		65. 4	128. 095174	122. 828631			
C		64. 9	127. 388112	119. 712112			
C	x	127. 8	68. 775087	66. 2734494			
C	x	167. 7	30. 5722995	30. 1955163			
C		81. 6	111. 564311	111. 291715			
C	x	133. 5	58. 2025766	62. 0206493			
C	x	163. 2	34. 3250061	34. 5506101			
C	x	98. 1	95. 8505882	95. 5230927			
C	x	138. 2	57. 6496827	57. 7168437			
C	x	103. 8	94. 7742967	95. 1237859			
C	x	165. 8	35. 7681723	35. 755644			
C	x	102. 7	95. 3472487	95. 4390424			
H		1. 43	30. 0562755	30. 00			
H		1. 9	29. 523834	29. 34			
H		2. 21	29. 3665741	29. 32			
H		3. 65	27. 6844334	27. 62			
H		4. 08	27. 1528416	27. 07			
H	x	6. 28	24. 7252154	25. 00			
H	x	6. 53	24. 7127335	24. 69			
H	x	6. 28	24. 9909073	25. 01			

Functional	Solvent?	Basis Set	Type of Data
mPW1PW91	PCM	6-31+G(d,p)	Shielding Tensors

	Isomer 1	Isomer 2	Isomer 3	Isomer 4	Isomer 5	Isomer 6
sDP4+ (H data)	55.79%	44.21%	–	–	–	–
sDP4+ (C data)	99.98%	0.02%	–	–	–	–
sDP4+ (all data)	99.98%	0.02%	–	–	–	–
uDP4+ (H data)	88.76%	11.24%	–	–	–	–
uDP4+ (C data)	100.00%	0.00%	–	–	–	–
uDP4+ (all data)	100.00%	0.00%	–	–	–	–
DP4+ (H data)	90.89%	9.11%	–	–	–	–
DP4+ (C data)	100.00%	0.00%	–	–	–	–
DP4+ (all data)	100.00%	0.00%	–	–	–	–

Table S17**.** The experimental and calculated chemical shifts (DP4+) of **15**

C	Exp. **15**	Calcd. **15-1** (TMS)	Calcd. **15-2** (TMS)	Calcd. **15-1** (DMSO)	Calcd. **15-2** (DMSO)
3	25.8	25.7	26.2	21.4	21.9
4	38.4	40.6	43.5	36.4	39.2
5	65.4	68.3	73.6	64.1	69.4
6	64.9	69.1	76.7	64.8	72.5
7	127.8	127.7	130.2	123.4	125.9
11	167.7	165.9	166.2	161.6	162.0
13	81.6	84.9	85.1	80.6	80.9
14	133.5	138.2	134.4	134.0	130.2
15	163.2	162.1	161.9	157.9	157.6
16	98.1	100.6	100.9	96.3	96.7
17	138.2	138.8	138.7	134.5	134.5
18	103.8	101.7	101.3	97.4	97.1
19	165.8	160.7	160.7	156.4	156.4
20	102.7	101.1	101.0	96.8	96.7
H	Exp. **15**	Calcd. **15-1** (TMS)	Calcd. **15-2** (TMS)	Calcd. **15-1** (DMSO)	Calcd. **15-2** (DMSO)
23	1.43	1.49	1.55	1.61	1.67
24	1.90	2.03	2.21	2.15	2.33
25	2.21	2.18	2.23	2.30	2.35
26	3.65	3.87	3.93	3.99	4.05
27	4.08	4.40	4.48	4.52	4.60
28	6.28	6.82	6.55	6.95	6.67
29	6.53	6.84	6.86	6.96	6.98
30	6.28	6.56	6.54	6.68	6.66
33	1.43	1.49	1.55	1.61	1.67
34	1.90	2.03	2.21	2.15	2.33

Table S18**.** The calculated shielding tensors of each conformer for isomer 1 (8*R**,9*S**,10a*R**-**15**)

C	**1-1**	**1-2**	**1-3**	**1-4**	**1-5**	**1-6**	**1-7**	**1-8**
3	170.5265	170.6822	170.5273	170.9025	171.0157	170.5216	170.7311	171.0255
4	155.477	156.0299	155.4725	155.8586	155.6086	157.838	156.0501	155.854
5	127.9892	126.9829	127.996	128.785	128.0568	126.7198	127.0507	127.6466
6	127.4034	128.4829	127.4101	127.2124	126.8724	127.9148	128.5075	126.825
7	68.1488	68.4411	68.1525	69.8517	68.1111	68.0359	68.4958	68.2548
11	30.5779	30.5781	30.5481	30.581	30.5142	30.5815	30.7212	30.6932
13	111.2764	111.7894	111.252	111.6897	111.5809	111.3791	111.9438	111.7188
14	58.9167	58.9979	58.9201	56.6299	59.1529	59.4114	59.1616	59.0363
15	34.3742	34.2923	34.3525	34.3292	34.315	34.3339	34.2063	34.2349
16	95.7541	95.9586	95.7453	95.8829	95.9501	95.9275	95.6301	95.6387
17	57.4412	57.4011	57.436	57.9622	57.5513	57.3596	57.5938	57.7433
18	94.9214	94.6695	94.9221	94.5944	94.7973	94.7141	95.17315	95.2719
19	35.8113	35.7315	35.8072	35.7642	35.7667	35.7728	35.6731	35.6835
20	95.5089	95.4933	95.5116	95.1342	95.4726	95.5268	95.17315	95.1173
H	**1-1**	**1-2**	**1-3**	**1-4**	**1-5**	**1-6**	**1-7**	**1-8**
23	29.8862	29.86	29.8859	29.894	29.8466	29.8297	29.8636	29.8483
24	30.1913	30.2379	30.1913	30.2252	30.2191	30.2157	30.2522	30.2338
25	30.0716	30.0989	30.0714	30.0733	30.0727	30.0651	30.1075	30.0772
26	29.51435	29.6538	29.5128	29.4832	29.5177	29.5316	29.6603	29.5154
27	29.1799	29.1236	29.1799	29.5886	29.4309	29.1904	29.1372	29.38195
28	27.6665	27.5165	27.6664	27.7349	27.7587	27.4784	27.526	27.7746
29	27.1322	27.0046	27.1318	27.2695	27.0757	27.198	27.0133	27.0831
30	24.73785	24.7369	24.73765	24.68095	24.7603	24.69605	24.79315	24.8146
33	24.73785	24.6691	24.73765	24.68095	24.693	24.69605	24.887	24.9131
34	25.015	25.0071	25.015	24.9925	25.0098	25.0094	24.79315	24.8146

Table S19**.** The calculated shielding tensors of each conformer for isomer 2 (8*S**,9*S**,10a*R**-**15**)

C	**2-1**	**2-2**	**2-3**	**2-4**	**2-5**	**2-6**	**2-7**	**2-8**
3	170.3594	170.1528	170.3686	170.0179	170.1921	170.4117	170.3583	170.0496
4	153.9054	151.6626	153.9136	152.6251	151.6858	153.6923	153.9084	152.6655
5	124.4264	121.6341	124.422	121.3244	121.673	122.4959	124.4229	121.3604
6	119.1458	120.9781	119.1494	119.4652	120.9482	118.617	119.1425	119.4652
7	65.465	66.7318	65.4768	66.9137	66.8225	66.7569	65.4697	66.9781
11	30.1662	30.1291	30.1429	30.1801	30.2777	30.3087	30.1585	30.3305
13	126.6705	125.5756	125.5761	125.6775	126.2629	126.5617	125.7933	125.7309
14	111.2262	111.4585	111.2025	110.8877	111.6157	111.3132	111.2141	111.0306

15	62.2087	62.3947	62.2066	62.4264	62.4601	60.2542	62.2091	62.4856
16	34.5814	34.5232	34.5616	34.5679	34.451	34.6121	34.5762	34.4982
17	95.51025	95.6375	95.51345	95.667	95.293	95.5322	95.509	95.3319
18	57.6742	57.6706	57.6754	57.5905	57.7908	57.9264	57.6682	57.7005
19	95.1145	95.0859	95.1196	94.9875	95.5186	94.8879	95.1121	95.4466
20	35.7811	35.737	35.7742	35.7373	35.6747	35.8174	35.7796	35.6773
H	**2-1**	**2-2**	**2-3**	**2-4**	**2-5**	**2-6**	**2-7**	**2-8**
23	30.07275	30.1095	30.0727	30.06745	30.0943	30.0655	30.07275	30.0776
24	29.8154	29.8129	29.8145	29.8133	29.8263	29.8164	30.07275	29.8283
25	30.07275	30.1095	30.0727	30.06745	30.1453	30.1252	29.8148	30.0776
26	29.3114	29.4065	29.3114	29.2759	29.4165	29.32005	29.3113	29.2849
27	29.3114	29.3163	29.3114	29.3796	29.3246	29.32005	29.3113	29.3871
28	27.5828	27.5412	27.5821	27.8456	27.5519	27.7126	27.5829	27.8591
29	27.0484	26.9712	27.0472	27.1169	26.981	27.299	27.0486	27.1288
30	25.0487	24.9424	25.0485	24.935	24.9879	25.02065	25.0485	24.9822
33	24.6732	24.6308	24.6734	24.6397	24.8486	24.6966	24.6733	24.8537
34	25.0487	25.0287	25.0485	25.0278	24.8486	25.02065	25.0485	24.8537

Table S20. DP4[+] probability analysis of **29** (mPW1PW91/6-31+G(d, p) level)

Functional		Solvent?		Basis Set		Type of Data	
mPW1PW91		PCM		6-31+G(d, p)		Shielding Tensors	
		DP4+	0.88%	99.12%	-	-	-
Nuclei	sp2?	xperimenta	Isomer 1	Isomer 2	Isomer 3	Isomer 4	Isomer 5
C		12.9	181.152198	181.278665			
C		23.3	171.333453	171.411157			
C	x	141.4	47.3621241	48.0505148			
C		77.4	119.166134	118.074918			
C	x	128.7	66.0134575	65.2677215			
C	x	135.4	68.9214161	65.8620611			
C		71.6	123.724365	122.407288			
C		66.2	129.414595	129.62552			
C	x	126.5	73.0484486	72.5192126			
C		31.5	156.728692	156.370926			
C	x	170.5	28.7441859	28.672947			
H		5.03	26.4634068	26.5521303			
H		1.02	30.5346643	30.5415828			
H		2.16	28.6276822	28.6404505			
H	x	6.54	24.7309198	24.72			
H	x	5.73	25.0434843	25.24			
H	x	5.83	25.3587452	25.13			
H		3.98	27.182378	27.24			
H		3.3	27.8327058	27.83			
H		3.09	28.4258835	28.54			
H		2.55	28.8915918	28.88			

Functional	Solvent?	Basis Set	Type of Data
mPW1PW91	PCM	6-31+G(d,p)	Shielding Tensors

	Isomer 1	Isomer 2	Isomer 3	Isomer 4	Isomer 5	Isomer 6
sDP4+ (H data)	66.90%	33.10%	–	–	–	–
sDP4+ (C data)	21.60%	78.40%	–	–	–	–
sDP4+ (all data)	35.77%	64.23%	–	–	–	–
uDP4+ (H data)	33.62%	66.38%	–	–	–	–
uDP4+ (C data)	3.05%	96.95%	–	–	–	–
uDP4+ (all data)	1.57%	98.43%	–	–	–	–
DP4+ (H data)	50.58%	49.42%	–	–	–	–
DP4+ (C data)	0.86%	99.14%	–	–	–	–
DP4+ (all data)	0.88%	99.12%	–	–	–	–

Table S21**.** The experimental and calculated chemical shifts (DP4+) of **29**

C	Exp. **29**	Calcd. **29-1** (TMS)	Calcd. **29-2** (TMS)	Calcd. **29-1** (DMSO)	Calcd. **29-2** (DMSO)
1	12.9	15.3	15.2	11.0	10.9
2	23.3	25.1	25.0	20.9	20.8
3	141.4	149.1	148.4	144.8	144.1
4	77.4	77.3	78.4	73.0	74.1
5	128.7	130.4	131.2	126.2	126.9
6	135.4	127.5	130.6	123.3	126.3
7	71.6	72.7	74.0	68.5	69.8
8	66.2	67.0	66.8	62.8	62.6
11	126.5	123.4	123.9	119.1	119.7
12	31.5	39.7	40.1	35.5	35.8
13	170.5	167.7	167.8	163.4	163.5
H	Exp. **29**	Calcd. **29-1** (TMS)	Calcd. **29-2** (TMS)	Calcd. **29-1** (DMSO)	Calcd. **29-2** (DMSO)
16	5.03	5.09	5.00	5.21	5.12
17	1.02	1.02	1.01	1.14	1.13
18	2.16	2.92	2.91	3.04	3.03
19	6.54	6.82	6.83	6.94	6.95
20	5.73	6.51	6.31	6.63	6.43
21	5.83	6.19	6.42	6.31	6.54
22	3.98	4.37	4.31	4.49	4.43
23	3.3	3.72	3.72	3.84	3.84
24	3.09	3.12	3.01	3.24	3.13
25	2.55	2.66	2.67	2.78	2.79
26	5.03	5.09	5.00	5.21	5.12
27	1.02	1.02	1.01	1.14	1.13
30	2.16	2.92	2.91	3.04	3.03
31	6.54	6.82	6.83	6.94	6.95

Table S22**.** The calculated shielding tensors of each conformer for isomer 1 (5*R**,3'*R**-**29**)

C	**1-1**	**1-2**	**1-3**	**1-4**	**1-5**	**1-6**	**1-7**	**1-8**	**1-9**	**1-10**
1	181.0276	181.2932	181.0596	181.4149	180.9688	181.5909	181.3231	181.0277	181.5178	181.0891
2	171.4732	171.2888	171.2832	171.2456	171.4301	171.6217	171.3215	171.382	171.4984	171.2253
3	48.5347	47.8019	47.5257	47.4208	46.4017	47.046	46.7916	47.36	46.5838	47.6685
4	116.4522	119.1457	119.016	119.6525	119.8337	119.6707	119.787	119.5621	119.7224	118.7777
5	69.8303	65.9151	65.8848	64.5943	63.9054	64.2199	64.2069	64.771	67.3371	69.6221
6	62.4416	68.9068	69.2045	66.8686	68.4591	68.4792	68.4217	66.6055	71.4449	71.9127
7	124.2979	123.9577	123.9101	120.2756	124.0614	120.5039	120.2438	120.2498	124.4691	124.3591
8	127.5954	129.9061	130.0747	129.8569	129.8784	130.0707	130.0793	130.0038	127.1213	127.0206
11	72.4294	72.3753	72.4553	72.0912	74.567	73.9702	73.7581	73.579	74.6119	72.6972
12	155.7729	155.7628	155.7871	158.9978	158.0566	162.7512	162.4982	158.7554	158.1073	155.5418
13	28.383	28.9453	28.9052	29.3114	28.2342	29.052	29.2893	29.0985	28.1143	28.8504
H	**1-1**	**1-2**	**1-3**	**1-4**	**1-5**	**1-6**	**1-7**	**1-8**	**1-9**	**1-10**
16	26.6832	26.4951	26.4525	26.5241	26.3719	26.4305	26.4137	26.5	26.4025	26.5094
17	30.3476	30.7713	30.7762	30.7559	30.4968	30.4617	30.4708	30.7712	30.4952	30.3434
18	30.7915	30.3132	30.4683	30.28345	30.8463	30.8037	30.8307	30.4377	30.3334	30.7683
19	30.4611	30.4741	30.3464	30.4716	30.3722	30.35065	30.35885	30.3353	30.7716	30.4592
20	29.6376	27.6494	29.6201	27.6469	27.5755	29.6598	27.6252	29.6203	29.6787	29.6186
21	27.65735	29.6234	27.6187	29.6057	29.6654	27.684	29.6548	27.6505	27.5836	27.6173
22	24.7837	24.7039	24.7675	24.665	24.7047	24.634	24.7074	24.7051	24.7127	24.7886
23	25.1293	24.9709	24.9797	25.03555	25.1561	25.3404	25.3248	25.0172	25.1683	24.9791
24	25.4131	25.3337	25.3254	25.03555	25.56	24.9991	25.0129	25.0172	25.5728	25.289
25	27.028	27.1795	27.1941	27.2946	27.2587	27.3842	27.3573	27.3042	27.0824	27.0059
26	28.3263	27.7159	27.7072	28.0762	27.7278	28.0624	28.0416	28.0607	28.3368	28.3018
27	27.65735	27.8806	27.8777	27.4507	27.9195	27.483	27.4517	27.4461	27.643	27.6173
30	28.7005	28.4604	28.4621	28.5033	28.2633	28.3906	28.3784	28.5221	28.2778	28.4838
31	28.7716	28.9656	28.9489	28.6563	28.8764	28.4409	28.4559	28.6506	28.867	28.9298

Table S23**.** The calculated shielding tensors of each conformer for isomer 2 (5*R**,3'*S**-**29**).

C	**2-1**	**2-2**	**2-3**	**2-4**	**2-5**	**2-6**	**2-7**	**2-8**	**2-9**	**2-10**
1	181.3439	181.5935	180.5913	181.4445	181.3749	181.4621	181.5072	181.005	181.3498	181.1313
2	171.5188	171.4653	171.3786	171.3467	171.3995	171.5897	171.3813	171.4472	171.4898	171.3936
3	48.243	46.8962	46.8216	48.1325	48.3108	46.8528	47.7871	48.405	48.6467	48.1936
4	116.5088	119.5396	118.9214	117.112	119.1235	119.6469	118.9552	116.8806	118.898	118.9463
5	65.9702	63.923	63.8991	64.4781	65.5768	67.3905	69.4684	64.6206	69.0215	65.5473
6	61.0911	68.2132	66.7402	62.6983	68.5423	71.0448	71.74	62.7193	66.3861	68.5239
7	121.2154	124.0035	124.1083	120.6495	124.0156	124.4312	124.523	120.7035	124.2215	123.9918
8	128.7534	130.0374	130.0938	130.1021	129.7635	127.127	127.0911	129.9143	128.8604	129.7311

11	72.7159	74.1601	74.0516	72.6545	71.936	74.2296	72.753	72.6371	71.6121	71.1354
12	155.9606	157.7631	156.0252	156.3829	155.7932	158.05	155.7765	156.5335	155.4009	155.7487
13	28.158	28.2794	28.0316	28.4803	29.2069	28.0698	29.068	28.356	29.0329	29.2541
H	**2-1**	**2-2**	**2-3**	**2-4**	**2-5**	**2-6**	**2-7**	**2-8**	**2-9**	**2-10**
16	26.7156	26.3196	26.2834	26.7358	26.4207	26.3324	26.4299	26.7015	26.4641	26.3995
17	30.3263	30.346	30.9236	30.4931	30.3298	30.809	30.7878	30.3453	30.494	30.3418
18	30.4731	30.822	30.3617	30.7854	30.5001	30.5089	30.331	30.7979	30.7892	30.8131
19	30.7561	30.5056	30.5166	30.3152	30.8057	30.3528	30.479	30.4732	30.3375	30.4827
20	27.6243	29.6836	27.8072	27.647	27.68485	29.6918	27.6325	29.6339	27.62387	29.6192
21	29.6448	27.6473	29.6294	29.6272	29.6284	27.6176	29.6226	27.6303	29.6363	27.6403
22	24.7511	24.7141	24.7715	24.7434	24.6978	24.7207	24.7668	24.7151	24.7244	24.7037
23	25.4857	25.1835	25.2205	25.4312	24.97	25.1965	25.0324	25.435	25.0752	24.9722
24	25.4857	25.5247	25.554	24.8608	25.3344	25.5076	25.3071	24.8798	25.1298	25.362
25	27.208	27.1761	27.1241	27.3447	27.1505	27.0201	27.0033	27.3635	27.1776	27.1438
26	27.8361	27.8571	27.8807	28.0934	27.68485	28.4451	28.3237	28.0942	27.62387	27.731
27	28.1483	27.955	27.9755	27.4678	27.8996	27.7106	27.6325	27.5003	27.62387	27.9055
30	28.6651	28.2802	28.3588	28.6611	28.4525	28.2653	28.4919	28.6693	28.4711	28.4357
31	28.8133	28.9059	29.0185	28.7941	28.9382	28.899	28.9826	28.8396	28.9478	28.9663
